# Cytonuclear Interactions in the Evolution of Animal Mitochondrial tRNA Metabolism

**DOI:** 10.1093/gbe/evv124

**Published:** 2015-06-27

**Authors:** Walker Pett, Dennis V. Lavrov

**Affiliations:** ^1^Department of Ecology, Evolution and Organismal Biology, Iowa State University; ^2^Present address: Laboratoire de Biométrie et Biologie Évolutive CNRS UMR 5558, Université Lyon 1, Villeurbanne, France

**Keywords:** transfer RNA, aminoacyl-tRNA synthetase, mitochondria, nonbilaterian animals

## Abstract

The evolution of mitochondrial information processing pathways, including replication, transcription and translation, is characterized by the gradual replacement of mitochondrial-encoded proteins with nuclear-encoded counterparts of diverse evolutionary origins. Although the ancestral enzymes involved in mitochondrial transcription and replication have been replaced early in eukaryotic evolution, mitochondrial translation is still carried out by an apparatus largely inherited from the α-proteobacterial ancestor. However, variation in the complement of mitochondrial-encoded molecules involved in translation, including transfer RNAs (tRNAs), provides evidence for the ongoing evolution of mitochondrial protein synthesis. Here, we investigate the evolution of the mitochondrial translational machinery using recent genomic and transcriptomic data from animals that have experienced the loss of mt-tRNAs, including phyla Cnidaria and Ctenophora, as well as some representatives of all four classes of Porifera. We focus on four sets of mitochondrial enzymes that directly interact with tRNAs: Aminoacyl-tRNA synthetases, glutamyl-tRNA amidotransferase, tRNA^Ile^ lysidine synthetase, and RNase P. Our results support the observation that the fate of nuclear-encoded mitochondrial proteins is influenced by the evolution of molecules encoded in mitochondrial DNA, but in a more complex manner than appreciated previously. The data also suggest that relaxed selection on mitochondrial translation rather than coevolution between mitochondrial and nuclear subunits is responsible for elevated rates of evolution in mitochondrial translational proteins.

## Introduction

All energy-producing mitochondria possess mitochondrial DNA (mtDNA), which is maintained and expressed by its own replication, transcription, and translation machinery. According to endosymbiotic theory, this machinery was inherited from the α-proteobacterial ancestor that gave rise to mitochondria, and should therefore resemble modern-day bacterial counterparts. However, in the more than 2 billion years since the origin of mitochondria, many of the proteins involved in mtDNA replication and transcription have been replaced by nonmitochondrial components (reviewed in Huynen et al. 2013). For example, mtDNA polymerase in opisthokonts (fungi and animals) resembles the DNA polymerase found in T-odd bacteriophages rather than eubacteria (Shutt and Gray 2006), suggesting a lateral gene transfer event from viruses (Filée et al. 2002). Similarly, mitochondrial transcription is performed by a phage-like single-subunit RNA polymerase (Bestwick and Shadel 2013), except in jakobid protists, which have retained the bacterial-like RNA polymerase (Burger et al. 2013).

In contrast to DNA replication and transcription, mitochondrial translation is still largely carried out by the molecular apparatus inherited from the α-proteobacterial ancestor of mitochondria (Adams 2003). One possible reason for this is that the key ribosomal RNA (rRNA) and transfer RNA (tRNA) components involved in mitochondrial translation are either exclusively (rRNA) or predominantly (tRNA) encoded by mtDNA. Nevertheless, extensive evolution in the composition of ribosomal protein genes has been reported (Smits et al. 2007; Scheel and Hausdorf 2014). Interestingly, multiple independent losses of mitochondrial tRNA (mt-tRNA) genes beyond the minimal number that is required for mitochondrial translation have also been observed (Schneider 2011). This loss of mt-tRNAs not only necessitates the import of nuclear-encoded tRNAs into mitochondria but also removes some evolutionary constraints on proteins formerly associated with mt-tRNAs. Thus, the effects of the replacement of mt-tRNAs can also extend to the nuclear-encoded components of mt-tRNA processing pathways.

In our previous studies (Haen et al. 2010; Pett et al. 2011), we investigated the impact that the loss of mt-tRNAs can have on the evolution of mitochondrial aminoacyl-tRNA synthetases (aaRS), which catalyze the attachment of tRNAs to their cognate amino acids. In nonphotosynthetic eukaryotes, there are usually three sets of aaRS, all of which are encoded in nuclear DNA: aaRS functioning in the mitochondrion (mt-aaRS), in the cytosol (cy-aaRS), or in both compartments (bifunctional aaRS). In two animal phyla, Cnidaria and Ctenophora, whose mt-genomes encode no more than two mt-tRNAs (Pett et al. 2011; Kayal et al. 2012), we identified only two mt-aaRS, despite locating homologs for all cy-aaRS (Haen et al. 2010). In contrast, we were able to identify all expected mt-aaRS in human and yeast, which posses a complete set of mt-tRNA genes. These results suggest that the loss of mt-tRNAs can potentially precipitate the loss of mt-aaRS. However, it is not clear whether the patterns observed in Cnidaria and Ctenophora can be extended to other groups that have also experienced a loss of mt-tRNA genes, including several lineages of sponges (Wang and Lavrov 2008).

Apart from tRNA aminoacylation, there are several additional tRNA processing pathways that are specific to the bacterial-like translation system inherited by mitochondria, and could therefore be subject to loss following the replacement of mt-tRNAs. For example, in opisthokonts, mitochondrial Asn-tRNA^Asn^ and Gln-tRNA^Gln^ are synthesized through an indirect aminoacylation pathway mediated by a multisubunit glutamyl-tRNA amidotransferase (Gat) complex that is not used in cytosolic translation (Frechin et al. 2009; Nagao et al. 2009). As part of this pathway, a nondiscriminating AspRS or GluRS forms a mischarged intermediate Asp-tRNA^Asn^ or Glu-tRNA^Gln^ (Curnow et al. 1997). These intermediates are then converted into the proper aminoacyl-tRNAs by Gat. In eukaryotes, Gat is composed of two catalytic subunits, GatA (also known as Qrsl1) and GatB (also known as PET112), both of which are required for amidotransferase activity (Nagao et al. 2009). A third subunit, GatC, acts as a linker between GatA and GatB, and is poorly conserved in eukaryotes, though it has been identified in both animals and plants (Pujol et al. 2008; Nagao et al. 2009).

Another tRNA processing pathway inherited by mitochondria from bacteria involves the use of a modified tRNA^Ile^(CAU) for the translation of the AUA codon as isoleucine. A specific enzyme, tRNA^Ile^-lysidine synthetase (TilS), catalyzes the conversion of cytosine in the wobble position of the anticodon loop to a lysidine (2-lysyl-cytidine), producing tRNA^Ile ^(LAU), which can then form a Watson–Crick base pair with AUA (Suzuki and Miyauchi 2010). This modification also prevents misaminoacylation of tRNA^Ile^ (LAU) by MetRS, and thus enables discrimination of AUA and AUG codons as isoleucine and methionine, respectively (Nakanishi et al. 2009). In contrast, discrimination of these codons is achieved by the use of tRNA^Ile^(ΨAU) in cytosolic translation, with the first nucleotide in the anticodon modified as pseudouridine (Senger et al. 1997). The gene encoding tRNA^Ile^ (CAU), *trnI(cau)*, is present in the mtDNA of many opisthokont species, and a putative TilS homolog has been identified in several species of fungi (Xavier et al. 2012). Among animals, however, *trnI(cau)* has been found only in sponges and placozoans, whereas it has been lost in bilaterians in parallel with a change in the specificity of the ATA codon from isoleucine to methionine (Lavrov 2007).

Finally, in both prokaryotes and eukaryotes, mature tRNAs are generated through the removal of extra 5′ nucleotides by a ribonucleoprotein known as RNase P, consisting of a ribozyme having catalytic activity associated with one or more proteins (Hall and Brown 2002; Kazantsev and Pace 2006). However, in the mitochondria of many eukaryotes, including plants and animals, the ancestral prokaryotic ribozyme has been replaced by a protein complex that is not homologous to the RNase P ribozyme utilized for the maturation of cytosolic small RNAs (Holzmann et al. 2008; Lai et al. 2010).

Here, we survey nuclear genomes from major groups of nonbilaterian animals as well as unicellular relatives of animals for the presence of major components of mt-tRNA processing pathways. Our study encompasses several representatives of the phylum Porifera that are known to have experienced mt-tRNA gene loss, including members of the homoscleromorph family Plakinidae, the demosponge subclass Keratosa*,* and a representative of the haplosclerid family Niphatidae, *Amphimedon queenslandica* (Wang and Lavrov 2008; Erpenbeck et al. 2009; Gazave et al. 2010). By comparing the results of this survey with our previous data from Cnidaria and Ctenophora, we show that the genomic background in which the loss of mt-tRNA genes occurs can have a unique impact on the evolution of nuclear-encoded genes.

## Materials and Methods

### Nuclear Genomic and Transcriptomic Data Acquisition

Raw or assembled nuclear genomic and/or transcriptomic data were retrieved from public databases or generated in our laboratory (table 1). Transcriptome assemblies for the sponge species *Aphrocallistes vastus, Chondrilla nucula, Corticium candelabrum, Crella elegans, Ircinia fasciculata, Petrosia ficiformis, Spongilla lacustris,* and *Sycon coactum* were downloaded from the Harvard Dataverse Network, doi:10.7910/DVN/24737 and doi:10.7910/DVN/24737 (Riesgo et al. 2012, 2014). Transcriptome assemblies for *Oscarella carmela* and *Ephydatia muelleri* were obtained from the Compagen database (Hemmrich and Bosch 2008); those for the octocoral *Gorgonia ventalina* (Burge et al. 2013) were acquired from FigShare (Burge et al. 2012). Transcriptome data for nine species of ctenophores (Moroz et al. 2014) were downloaded from the Pleurobrachia Neurobase Database (neurobase.rc.ufl.edu/pleurobrachia/browse, last accessed June 2014). Additional RNA-Seq libraries were obtained for the filisterean *Ministeria vibrans,* and the mesomycetozoan *Creolimax fragrantissima* (accessible on the NCBI [National Center for Biotechnology Information] Sequence Read Archive, SRR1029670 and SRR343051 respectively)*,* with assemblies courtesy of Iñaki Ruiz-Trillo at the Institut de Biologia Evolutiva (WP personal communication, April 2014).

**Table 1:** Nuclear Data Sets Used for Phylogenetic Profiling of mt-tRNA Processing Enzymes

Species	Data Type			Source
	P	T	G	
Icthyosporea				
*Creolimax fragrantissima*		x		a
*Sphaeroforma arctica*	x			b
Filasterea				
*Capsaspora owczarzaki*	x			b
*Ministeria vibrans*		x		a
Choanoflagellata				
*Monosiga brevicollis*	x			b
*Salpingoeca rosetta*	x			b
Porifera				
Hexactinellida				
*Aphrocallistes vastus*		x		c
Demospongiae				
Haploscleromorpha				
*Amphimedon queenslandica*	x			e
*Petrosia ficiformis*		x		c
Heteroscleromorpha				
*Crella elegans*		x		c
*Ephydatia muelleri*		x		f
*Spongilla lacustris*		x		c
Keratosa				
*Ircinia fasciculata*		x		c
Myxospongiae				
*Chondrilla nucula*		x		c
Homoscleromorpha				
Plakinidae				
*Corticium candelabrum*		x	**x**	c,d
Oscarellidae				
*Oscarella carmela*	x	x		f
Calcarea				
*Clathrina* sp.			x	g
*Sycon coactum*		x		c
Ctenophora				
*Beroe abyssicola*		x		h
*Bolinopsis infundibulum*		x		h
*Coeloplana astericola*		x		h
*Dryodora glandiformis*		x		h
*Euplokamis dunlapae*		x		h
Mertensiidae sp.		x		h
*Mnemiopsis leidyi*	x	x		h,i
*Pleurobrachia bachei*	x	x		h
*Vallicula multiformis*		x		h
Placozoa				
*Trichoplax adhaerens*	x			j
Cnidaria				
Anthozoa				
Hexacorallia				
*Nematostella vectensis*	x			j
Octocorallia				
*Gorgonia ventalina*		x		k
Medusozoa				
*Aurelia aurita*		x		f
*Hydra magnapapillata*	x			j

Note.—P, predicted proteome; G, genome assembly; T, transcriptome assembly. a, NCBI Sequence Read Archive (SRR102967, SRR34305); b, Broad Institute origins of Multicellularity Database; c, Harvard Dataverse Network, doi:10.7910/DVN/25071, doi:10.7910/DVN/24737; d, FigShare, doi: 10.6084/m9.figshare.141268; e, EnsemblMetazoa 25; f, Compagen Database; g, Sars International Center for Marine Molecular Biology (DL personal communication); h, *Pleurobrachia* Neurobase (neurobase.rc.ufl.edu/pleurobrachia/browse); i, NIH *Mnemiopsis* Genome Portal; j, Joint Genome Institute; k, FigShare, doi: 10.6084/m9.figshare.9432.

We downloaded predicted proteomes from the complete genomes of *Amphimedon queenslandica* (Srivastava et al. 2010)*, Mnemiopsis leidyi* (Ryan et al. 2013), *Pleurobrachia bachei* (Moroz et al. 2014), *Trichoplax adhaerens* (Srivastava et al. 2008), *Nematostella vectensis* (Putnam et al. 2007), from their respective databases (table 1), and proteomes for *Monosiga brevicolllis, Salpingoeca rosetta, Capsaspora owczarzaki, Sphaeroforma arctica* from the Origins of Multicellularity Database (Ruiz-Trillo et al. 2007). In addition, a whole-genome assembly from a Calcinean calcareous sponge *Clathrina* sp. was shared with us by Maja Adamska and Marcin Adamski at the Sars International Centre for Marine Biology (DL personal communication, April 2014).

Finally, we used Illumina DNAseq data for *C. candelabrum* generated in our lab. This data set was generated as described in Haen et al. (2014) from a specimen collected at the Endoume marine station in Marseille, France. A total of 59,853,912 paired-end reads were assembled using SOAPdenovo2 (Luo et al. 2012) with a k-mer size of 63, and the raw reads have been placed in the NCBI Sequence Read Archive under accession SRR2021567. The assembled data set has been placed on FigShare (doi: 10.6084/m9.figshare.1412681).

### Mitochondrial Sequence Data Acquisition

**Fig. 1.—:**
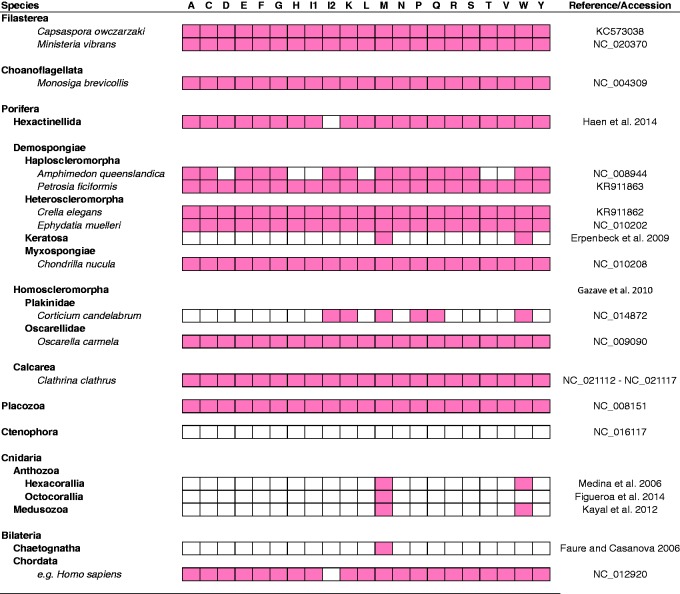
Mitochondrial-encoded tRNA gene content in animals and unicellular relatives. For each group or species, filled squares indicate the presence of an mt-tRNA gene of the amino acid identity indicated at the top of each column. Gene content was determined using previously published annotated sequences or by surveying relevant literature (Reference/Accession column). The labels I1 and I2 indicate genes for mt-tRNA^Ile^(GAU) and mt-tRNA^Ile^(CAU), respectively.

mt-tRNA content was determined based on previously published/annotated mt-genomes (fig. 1). Individual mt-tRNA sequences were extracted from mitochondrial genomes of the protozoans *Ca. owczarzaki* KC573038, and *Mo. brevicollis* NC_020370; the homoscleromorph *O. carmela* NC_009090; demosponges *Amphimedon compressa* NC_010201, *A. queenslandica* NC_008944, *Aplysina cauliformis* NC_016949, *Axinella corrugata* NC_006894, *Chondrilla nucula* NC_010208, *E. muelleri* NC_010202, *Geodia neptuni* NC_006990, *Igernella notabilis* NC_010216, *Negombata magnifica* NC_010171, *Suberites domuncula* NC_010496, *Tethya actinia* NC_006991, and *Xestospongia muta* NC_010211; the glass sponges *Ap. vastus* NC_010769, *Hertwigia falcifera* KM580071, *Iphiteon panacea* EF537576, *Sympagella nux* EF537577, *Tabachnickia* sp. KM580074, *Vazella pourtalesi* KM580075; the placozoan *T. adhaerens* NC_008151; the cnidarian *Nematostella* sp. NC_008164; and the bilaterians *Anopheles gambiae* NC_002084, *Caenorhabditis elegans* NC_009885, *Strongylocentrotus purpuratus* NC_001453, and *Homo sapiens* NC_012920.

Complete mtDNA of *P. ficiformis* was amplified in two fragments by LA-Taq PCR (polymerase chain reaction) (Takara), which were pooled with other samples and submitted to the Iowa State University DNA Facility for library preparation and sequencing. The DNA library was prepared using the TruSeq kit and sequenced on the Illumina HiSeq 2500 platform. The mtDNA sequences were assembled with a modified PCAP package (Huang et al. 2003; DL personal communication). Assembled mtDNA sequence was compared and found nearly identical with partial assemblies from *P. ficiformis* RNA-Seq data (Riesgo et al. 2014). The complete mtDNA from *Cr. elegans* was also assembled using a modified PCAP packaged from RNA-Seq data obtained at the NCBI Sequence Read Archive, accessions SRR648558, SRR648671, and SRR648683. The complete mtDNA sequences from *Cr. elegans* and *P. ficiformis* have been deposited in GenBank under the accessions KR911862 and KR911863, respectively.

### Phylogenetic Profiling for Target Genes

Eukaryotic sequences for each type of aaRS and the subunits of GatCAB were obtained from the Homologene database and multiple sequence alignments of these sequences were constructed using MAFFT 6 with the –globalpair option (Katoh and Toh 2008). We constructed Hidden Markov Models (HMMs) using HMMER 3 for these alignments (Finn et al. 2011), which were then used to search the genome or transcriptome of each target organism with an *e*-value cutoff of 1e-5. BLASTP searches were conducted against the NCBI RefSeq database using the resulting hits to check for homology with the target gene. Regions from the resulting set of sequences that aligned with the eukaryotic HMMs were saved for phylogenetic analysis.

When no genomic sequences were available to refine transcriptomic data, HMM searches often produced a very large number of fragmentary hits, which aligned only partially to the query profile, or which showed high similarity with bacterial sequences. Thus, a phylogenetic profiling step was performed to identify homologous eukaryotic fragments and filter out prokaryotic contaminants. First, prokaryotic alignments were constructed based on reviewed amino acid sequences from reference proteome sets contained in the UniProt database. These alignments were combined with the previously constructed eukaryotic alignments and used to estimate phylogenetic trees with PhyML 3.0 (see phylogenetic analysis) (Guindon et al. 2010). The identities of partial transcripts obtained in the HMM profiling step were determined using pplacer (Matsen et al. 2010), by calculating the maximum-likelihood branching point for each fragment on the corresponding phylogenetic tree, conditioned on the alignment used to construct the tree and previous maximum-likelihood estimates of the branch lengths and other model parameters. Sequences that were placed inside of a eukaryotic lineage were retained for further analysis. Following this initial round of phylogenetic profiling, new HMM profiles were constructed from subalignments containing only eukaryotic sequences. These were then used to conduct a repeated round of phylogenetic profiling to verify the absence of some more poorly conserved genes that may have been represented only by short fragments that do not share a high degree of similarity with sequences in the Homologene database. Candidate homologs identified by this procedure were checked for structural homology with the target sequences using the Phyre^2^ protein fold prediction server (Kelley and Sternberg 2009).

To identify TilS homologs, fungal TilS sequences were obtained based on the accession numbers listed in Xavier et al. (2012). These sequences were used to construct an HMM profile, which was then utilized to query each target genome and/or transcriptome. The HMM profiles, sequence alignments used to generate them and all sequence alignments, trees and PhyML statistics files used during the pplacer profiling step have been made available on FigShare (doi: 10.6084/m9.figshare.1412684).

### Phylogenetic Analysis

We utilized PROTTEST (Darriba et al. 2011) to estimate the best-fitting model of amino acid replacement for each alignment used in the profiling step. In each case, WAG (Whelan and Goldman 2001) was identified as the best-fitting model. Trees were then constructed for the profiling step using PhyML 3.0 with the WAG+Γ model (supplementary fig. S1, Supplementary Material online). Additional Bayesian phylogenetic analyses were conducted with PhyloBayes MPI, using the WAG+Γ or CAT+WAG+Γ model (Lartillot et al. 2013) (supplementary figs. S2 and S3, Supplementary Material online). Phylogenetic analysis of mt-tRNA^Met^ and mt-tRNA^Ile^ sequences was conducted in MrBayes 3.2, using a reversible-jump mixture of all possible nucleotide substitution models (Ronquist et al. 2012) (supplementary fig. S4, Supplementary Material online).

### Estimation of Substitution Rates in mt-aaRS and mt-tRNAs

Substitution rates in aaRS sequences were estimated based on mean maximum-likelihood WAG distances between aaRS from *Ca. owczarzaki* and sequences from ingroup choanozoan species, computed with the R package phangorn (Schliep 2011). mt-tRNA sequences from *Ca. owczarzaki, Mo. brevicollis, O. carmela, C. nucula, E. muelleri, Suberites domuncula, A. queenslandica, T. adhaerens, Hydra magnapapillata, Anopheles gambiae, Caenorhabditis elegans, Strongylocentrotus purpuratus,* and *H. sapiens* were aligned using the TRNA2 model in the COVE package (Eddy and Durbin 1994). Rates of substitution were estimated using mean maximum-likelihood pairwise Kimura 2 Parameter (K80) distances between mt-tRNAs from *Ca. owczarzaki* and each other species, also estimated using phangorn.

### Testing for Compensatory Substitutions in mt-aaRS

In order to test for a contribution from compensatory substitutions on mt-aaRS substitution rates, we assessed the fit of the following linear model describing the relationship between *Y_i_*, the substitution rate in aaRS *i*, and *X_i_*, the substitution rate in mt-tRNA *i* of the same amino acid specificity:

$$Y_{i}=\beta_{0}+\beta_{\text{1}}\delta_{\text{mt}}+\beta_{\text{2}}X_{i}+\beta_{\text{3}}X_{i}\delta_{\text{mt}}+\varepsilon_{i},$$

where *δ_mt_* is an indicator function equal to 1 if aaRS *i* is inferred to be mitochondrial, *β_1_* represents the increase in substitution rates in mt-aaRS resulting from relaxed selection on mitochondrial translation, *β_2_* represents the relationship between substitution rates in aaRS and mt-tRNAs resulting from shared amino acid specificity, and *β_3_* represents the interaction between substitution rates in mt-tRNAs and mt-aaRS (i.e., the contribution from compensatory substitutions). We also assessed the relative fit of two nested cases in which *β_3_* = 0, and *β_3_* = *β_2_* = 0, respectively.

## Results and Discussion

### Mitochondrial aaRS Present before the Emergence of Metazoa

**Fig. 2.—:**
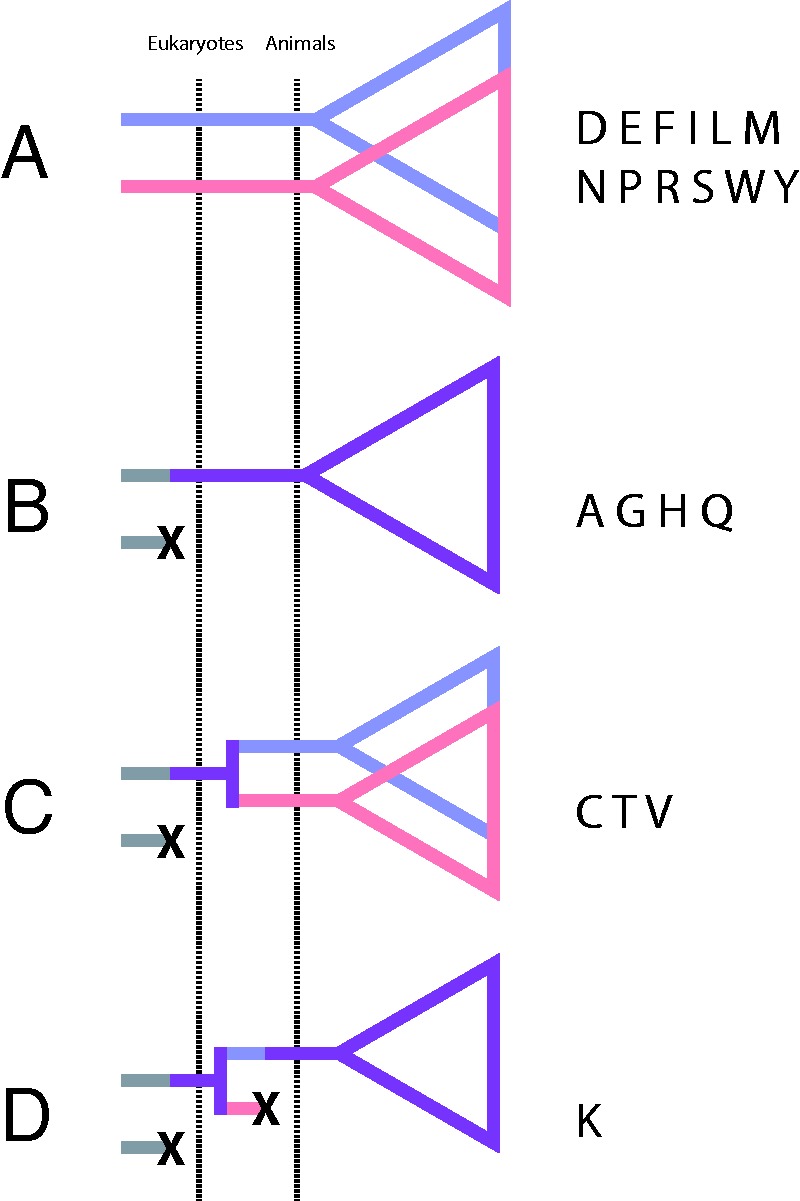
Mitochondrial aaRS families originating before the divergence of animals. Schematic representation of different types of aaRS families according to the time of origin or loss of a distinct mitochondrial lineage. Blue and red lineages correspond to mitochondrial and cytosolic aaRS families, respectively. Purple lineages signify aaRS families represented by a single eukaryotic lineage, and gray lineages indicate families of unknown localization. Dashed lines indicate the time of origin of eukaryotes and animals. (*A*) Families with distinct mitochondrial and cytosolic lineages before and after the origin of animals. (*B*) Families in which either the mitochondrial or cytosolic variant was lost before the divergence of eukaryotes. (*C*) Families in which either the mitochondrial or cytosolic lineage was lost prior to the divergence of eukaryotes, but was subsequently reestablished through duplication before the divergence of animals. (*D*) LysRS family in which either the mitochondrial or cytosolic lineage was lost prior to the divergence of eukaryotes, reestablished before to the emergence of Choanozoa, then lost again before the divergence of animals.

As a first step in elucidating the patterns of replacement of mt-tRNA processing activities in animals, we conducted phylogenetic analyses of individual aaRS genes families to identify lineages present before the divergence of Metazoa (fig. 2supplementary fig. S1, Supplementary Material online). In agreement with previous studies (Brindefalk et al. 2007; Brandão and Silva-Filho 2011), we identified eight aaRS genes where either the mitochondrial or cytosolic lineage was lost early in eukaryotic evolution (AlaRS, CysRS, GlyRS, HisRS, LysRS, GlnRS, ThrRS, and ValRS) (fig. 2*B*–*D* and supplementary fig. S1*A*, *B*, *G*, *H*, *J*, *Q*, and *R*, Supplementary Material online). Four of these genes (CysRS, LysRS, ThrRS, and ValRS) have undergone duplication prior to the divergence of Choanozoa (animals and choanoflagellates) from other opisthokonts (fig. 2*C* and *D* and supplementary fig. S2, Supplementary Material online), restoring separate mitochondrial and cytosolic aaRS lineages. Subsequently, mt-LysRS appears to have been lost again in the lineage leading to animals, leaving animal LysRS sequences more closely related to cy-LysRS rather than mt-LysRS sequences from choanoflagellates (fig. 2*D* and supplementary fig. S2*B*, Supplementary Material online). The remaining 12 aaRS families have maintained distinct mitochondrial and cytosolic lineages both before and after the divergence of animals (fig. 2*A*), resulting in a total of 15 specialized mt-aaRS genes that are inferred to have been present in the common ancestor of Metazoa.

### Elevated Substitution Rates in mt-aaRS Are the Result of Relaxed Purifying Selection

**Fig. 3.—:**
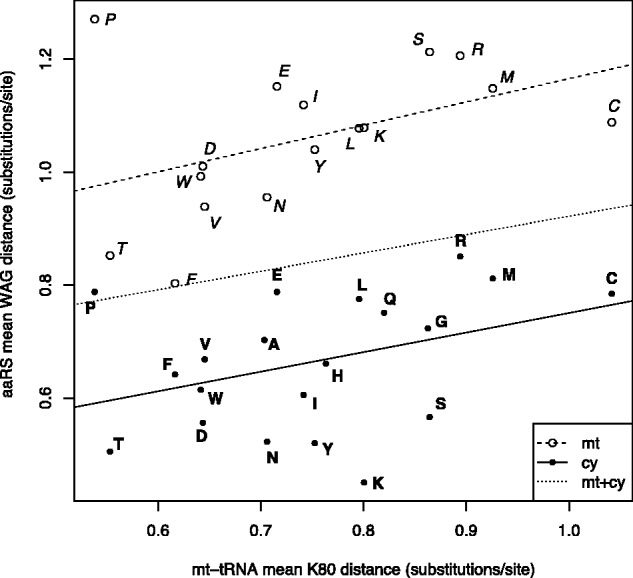
Elevated substitution rates in mt-aaRS due to relaxed selection. Mean pairwise distances between aaRS and mt-tRNA sequences from *Ca. owczarzaki* and members of Choanozoa are shown. Maximum-likelihood distances were estimated between aaRS sequences using the WAG model, and between mt-tRNA sequences using the Kimura 2-Parameter (K80) model (mt, mitochondrial; cy, cytosolic/bifunctional). Letters in italics with open circles indicate mt-aaRS, and letters in boldface with filled circles indicate cytosolic or bifunctional aaRS. Dashed and solid lines represent linear regression estimates of the relationship between substitution rates in mt-tRNAs and mt-aaRS or cy-aaRS, respectively, and the finely dotted line represents the combined relationship between mt-tRNAs and both types of aaRS. The most parsimonious linear model showed that substitution rates in mt-aaRS and cy-aaRS were significantly different (line intercepts, *P* < 0.0001), and positively correlated with substitution rates in mt-tRNAs (combined slope > 0, *P* = 0.015), but there was no significant difference between aaRS in their relationship with substitution rates in mt-tRNAs (line slopes, *P* = 0.83).

For every family of aaRS with both mitochondrial and cytosolic counterparts, we observed significantly higher substitution rates in mt-aaRS than in corresponding cy-aaRS (*β_1_* = 0.39, *P* < 0.0001) (fig. 3). The accelerated rate of substitution observed in many nuclear-encoded mitochondrial proteins is often attributed to compensatory adaptive evolution occurring between nuclear and mitochondrial genomes (Kern and Kondrashov 2004; Osada and Akashi 2012). To test whether compensatory evolution can explain substitution patterns in aaRS, we compared substitution rates in mt-aaRS, cy-aaRS, and mt-tRNAs. Indeed, we found that substitution rates in mt-aaRS were weakly correlated with substitution rates in mt-tRNAs (*β_2_* = 0.38, *P* = 0.015). Surprisingly, we found a similar correlation between substitution rates in mt-tRNAs and cy-aaRS, with no significant difference between the two groups of aaRS (*β_3_* = 0.07, *P* = 0.83). We also found a positive correlation between substitution rates in mt-aaRS and cy-aaRS of the same amino acid specificity (*r* = 0.5, *P* < 0.05). We hypothesize that the observed correlations are not the result of an increased rate of compensatory changes in mt-aaRS in response to higher rates of evolution in mt-tRNAs, but are instead due to some shared constraints on aaRS and their cognate tRNAs. One possibility is the number of specific tRNA identity elements recognized by aaRS, which constraints the evolution of both molecules. Then, elevated substitution rates in mt-aaRS are expected merely as an outcome of relaxed selection pressure on mitochondrial translational proteins due to the small size of the mitochondrial-encoded proteome. We suspect that this pattern may also help explain the high rate of substitution observed in other nuclear-encoded mitochondrial proteins that interact with mitochondrial-encoded RNAs including plant ribosomal proteins, which has been argued to be the result of compensatory evolution (see also Sloan et al. 2014).

### Parallel Losses of mt-tRNAs and mt-aaRS in Nonbilaterian Animals

**Fig. 4.—:**
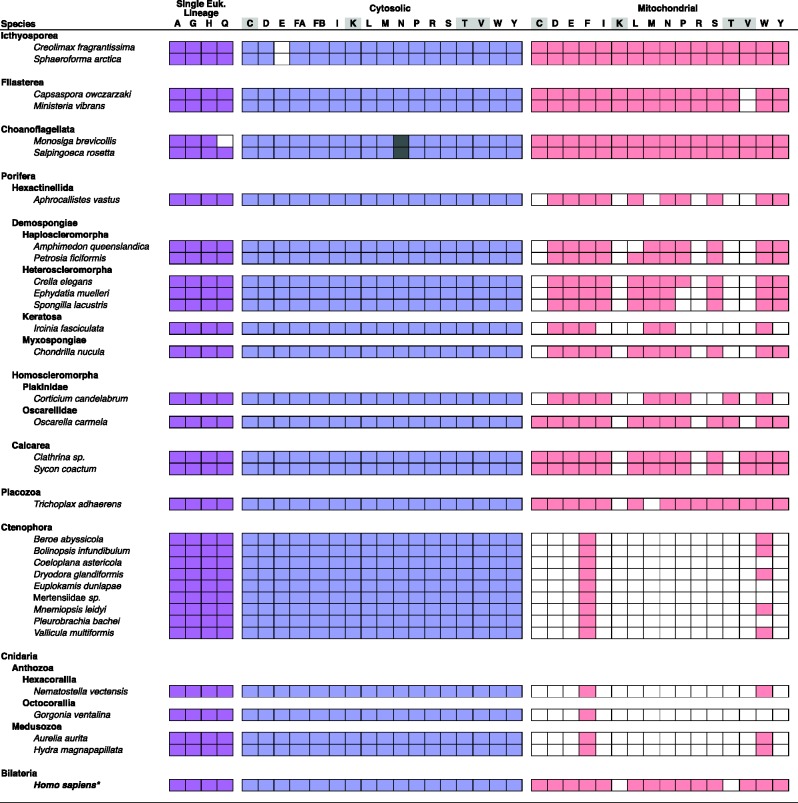
Phylogenetic profiling for nuclear-encoded aaRS homologs. For each species, filled squares signify the successful identification of aaRS homologs using a maximum-likelihood phylogenetic profiling pipeline (see Materials and Methods). Blue and red squares indicate the presence of aaRS homologs from either a cytosolic and mitochondrial lineage, respectively (fig. 2). Purple cells correspond to aaRS from a single eukaryotic lineage (fig. 2*B*). Columns marked by amino acids shaded in gray indicate aaRS lineages that emerged through duplication of a single eukaryotic ancestor (fig. 2*C* and *D*). Dark gray boxes correspond to the choanoflagellate AsnRS that group with sequences from plants (supplementary fig. S4*M*, Supplementary Material online). The * by *H. sapiens* indicates results that were obtained from previously annotated sequences in the Homologene database (accession numbers can be found in supplementary material, Supplementary Material online).

In all animal lineages that have experienced losses of mt-tRNA genes, we observed parallel losses of many corresponding mt-aaRS, suggesting that the retention of these enzymes depends primarily on the presence of corresponding mt-tRNA substrates (figs. 4^5^). Previously we have shown that in both Cnidaria and Ctenophora, whose mtDNA encodes no more than two mt-tRNAs, only two mt-aaRS have been retained (Haen et al. 2010; Pett et al. 2011). One of them—mt-TrpRS—appears to be required for the aminoacylation of mt-tRNA^Trp^, which recognizes the TGA codon translated as tryptophan in animal mitochondria, in addition to the standard TGG codon.

**Fig. 5.—:**
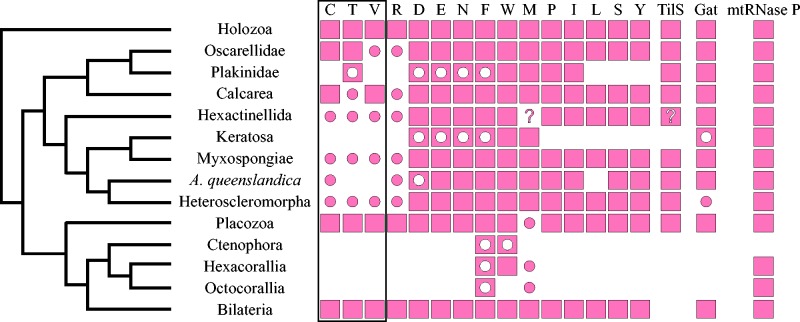
Distribution of mitochondrial-encoded tRNAs and nuclear-encoded mt-tRNA processing enzymes in Metazoa. Filled squares denote the presence of a nuclear-encoded mt-tRNA processing activity and corresponding mitochondrial-encoded tRNA substrates in the common ancestor of the indicated taxon. Filled circles on an empty background signify the presence of an mt-tRNA substrate for which the corresponding processing enzyme is absent. Empty circles within filled squares indicate the presence of the enzyme in the absence of a tRNA substrate. Empty cells indicate the absence of both the enzyme and the substrate. Question marks refer to the ambiguous substrate affinity of a single mt-tRNAMet encoded in the mt-genomes of Hexactinellida. Single letters at the top of the columns signify the amino acid specificity of mt-aaRS and corresponding tRNAs. Only aaRS inferred to have a separate mt-aaRS lineage in the common ancestor of animals are shown, with the boxed columns indicating those inferred to have originated through a gene duplication after the divergence of eukaryotes. The tree on the left is a combination of those in Philippe et al. (2009), Gazave et al. (2010), and Cárdenas et al. (2012).

Interestingly, both octocoral cnidarians and ctenophores have lost the gene encoding mt-tRNA^Trp^(UCA) from their mtDNA. In addition, octocorals use few, if any, TGA codons in their mitochondrial coding sequences (Pont-Kingdon et al. 1998; Brugler and France 2008). Consistent with these observations, our search of the transcriptome of the octocoral *G. ventalina* failed to identify a homolog of mt-TrpRS (fig. 4). Thus, we posit that at least some octocoral cnidarians use cytosolic tRNA^Trp^ in mitochondrial translation, and have reverted to the standard genetic code in mitochondrial protein synthesis*.* Similarly, we did not find mt-TrpRS in *Pl. bachei,* where the mitochondrial TGA codon appears to have been recruited for an amino acid other than tryptophan (Kohn et al. 2012). Comparative sequence analysis of the mt-genome from *Pl. bachei* (NC_016697) using GenDecoder (Abascal et al. 2006) showed that 38% of TGA codons in highly conserved positions (entropy < 1.0, gaps < 20%) correspond to serine in other animals, suggesting a novel reassignment of TGA to serine in the mtDNA of this species. In contrast, we successfully identified mt-TrpRS in *M. leidyi* where no TGG codons are present in mtDNA and thus TGA is still translated as tryptophan (Pett et al. 2011). Thus, in both Cnidaria and Ctenophora, the loss of mt-tRNA^Trp^ and replacement of mt-TrpRS appear to coincide with modifications to the mitochondrial genetic code.

Several parallel losses of mt-tRNAs and mt-aaRS have also occurred in sponges (fig. 4^5^). First, *I. fasciculata,* which encodes only two tRNA genes in its mitochondrial genome, has lost nine mt-aaRS, while retaining those corresponding to the two remaining mt-tRNAs (Met, Trp). Second, *C. candelabrum* encodes only six tRNAs in mtDNA (Ile, Lys, Met, Pro, Gln, Trp), and appears to have lost both mt-tRNAs and mt-aaRS corresponding to Cys, Leu, Arg, Ser, Val, and Tyr. Finally, *A. queenslandica* has lost both mt-tRNAs and mt-aaRS corresponding to Leu, Thr, and Val.

### Retention of mt-aaRS But the Loss of mt-tRNAs

Although the loss of most mt-aaRS seems to be limited primarily by the presence of mt-tRNAs, that of some mt-aaRS appear to be constrained by additional factors, such as the efficiency of mitochondrial import of cytosolic aaRS and/or involvement of mt-aaRS in additional biochemical pathways. Perhaps, the best example of the first type of constraint is mt-PheRS. This enzyme is unusual in that the mitochondrial variant consists of a single monomeric subunit, whereas the cytosolic variant is a heterodimer composed of an α and β subunits (Roy et al. 2005). The heterodimeric structure of cy-PhrRS makes the mitochondrial import of a functional enzyme highly unlikely (Haen et al. 2010). Consequently, mt-PheRS has never been replaced in animals, despite at least three independent losses of mt-tRNA^Phe^ (figs. 1^5^).

In addition, we were able to identify mt-GluRS, mt-AspRS, and mt-AsnRS in every lineage of sponges we examined, including species that do not encode corresponding mt-tRNAs, indicating that these enzymes may carry out functions distinct from the direct aminoacylation of mt-tRNAs (fig. 4^5^). In particular we found homologs of each of these enzymes in *A. queenslandica* whose mt-DNA does not encode mt-tRNA^Asp^, as well as *I. fasciculata* and *C. candelabrum*, whose mt-DNAs do not encode mt-tRNA^Glu^, mt-tRNA^Asp^, or mt-tRNA^Asn^. Furthermore, alignments with structurally characterized aaRS sequences show that the mt-GluRS sequence we identified from *I. fasciculata* and the mt-AspRS sequences from *A. queenslandica* and *C. candelabrum* contain conserved anticodon binding domains, suggesting that the encoded enzymes have retained tRNA processing functions (data not shown).

### Losses of mt-aaRS in the Presence of mt-tRNAs

Finally, the replacement of some mt-aaRS does not appear to be strongly limited by either the presence of a corresponding tRNA substrate or additional functional constraints. First, we found no distinct mt-ArgRS sequence in any sponge data set we examined, suggesting that this gene was lost before the divergence of Porifera (fig. 4). Outside of animals, independent losses of mt-ArgRS have likely occurred within plants and fungi (supplementary fig. S1*O*, Supplementary Material online). Second, we found no mt-CysRS homolog in any demosponge or glass sponge data set, suggesting that this gene was lost before the divergence of siliceous sponges (fig. 4figs. S1*B* and S2*A*, Supplementary Material online). Third, we could not identify mt-ThrRS in either siliceous or calcareous sponges, but found it in both homoscleromorph data sets, indicating at least two independent losses of this enzyme in Porifera (fig. 4supplementary fig. S2*C*, Supplementary Material online). As has been noted previously (Haen et al. 2010), mt-ThrRS is also absent in Ctenophora, Cnidaria and bilaterian animals except vertebrates, where it was restored through a duplication of cy-ThrRS (supplementary fig. S1*Q*, Supplementary Material online). Fourth, we found that among sponges, mt-ValRS is present only in calcareous sponges (Class Calcarea), and therefore has most likely been lost independently in both homoscleromorphs and siliceous sponges (figs. 4^5^supplementary figs. S1*R* and S2*D*, Supplementary Material online). Finally, we found no mt-ProRS in representatives of the demosponge order Spongillida, which includes freshwater sponges *S. lacustris* and *E. muelleri* (fig. 4supplementary fig. S1*N*, Supplementary Material online).

Assuming the loss of the aforelisted mt-aaRS lineages, the reason for the retention of the corresponding mt-tRNA genes in mtDNA is unclear. It is possible that the import of cytosolic tRNAs is not directly linked to the import of cy-aaRS in these cases, and would require additional pathways. Another possibility is that these mt-tRNAs have been recruited for additional functions beyond aminoacylation, for example as structural RNA components of the mitochondrial ribosome, as has been observed in mammalian mitochondria (Brown et al. 2014; Greber et al. 2014).

### A Complete GatCAB Is Present in Porifera, Placozoa and Bilateria, but Absent from Cnidaria and Ctenophora

**Fig. 6.—:**
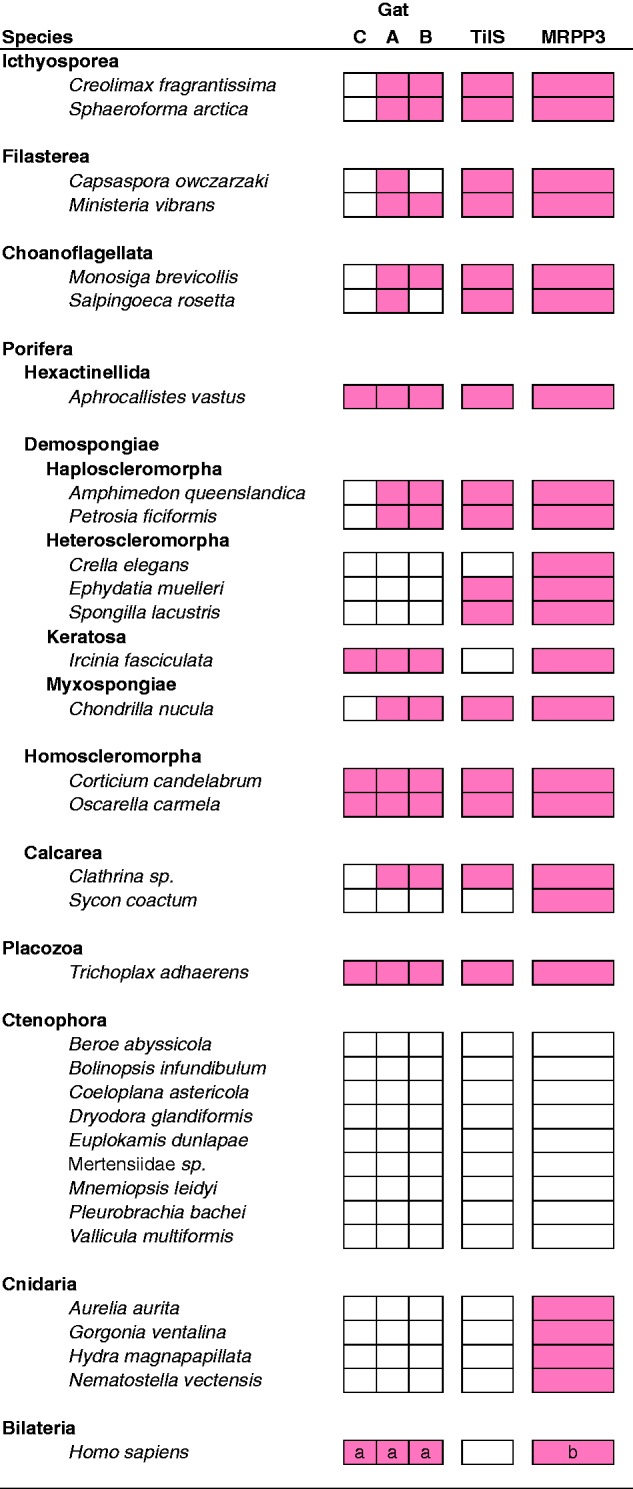
Phylogenetic profiling for nuclear-encoded GatCAB, TilS, and MRPP3 homologs. For each species, filled squares indicate the successful identification of a mt-tRNA processing enzyme. Letters in *H. sapiens* indicate results that were obtained in previous studies (a, Nagao et al. 2009; b, Reinhard et al. 2015). The absence of TilS in human was verified by our profiling procedure using version GRCh38 of the Ensembl human proteome assembly.

We searched for and were able to locate homologs of Gat subunits A and B in many of the data sets we examined (fig. 6). GatA homologs fell into one of two main clades, one containing animal sequences including the known GatA from human (Nagao et al. 2009), and one consisting of putatively paralogous, GatA-related proteins (supplementary figs. S3*A* and S4*U*, Supplementary Material online). We considered GatA sequences as orthologous to the human mitochondrial protein only if they belonged to the former clade. In contrast, all GatB homologs, including known mitochondrial sequences, formed a monophyletic clade in agreement with previous results (Sheppard and Söll 2008) (supplementary figs. S3*B* and S4*V*, Supplementary Material online). We were able to identify homologs of mitochondrial GatA and GatB in Placozoa, and in representatives from each class of sponges, though we failed to identify related sequences in many demosponges, including those in the group known as Heteroscleromorpha (Cárdenas et al. 2012), as well as the Calcaronean sponge *Sy. coactum*, whose mtDNA has not been determined. The small and poorly conserved GatC subunit was more difficult to identify, though we were able to locate homologs in some sponges, including *I. fasciculata* and *C. candelabrum*. In contrast to sponges, we failed to identify homologs of GatA, GatB or GatC in Cnidaria or Ctenophora, suggesting that mt-tRNA amidotransferase activity is not present in the mitochondria of these species.

Given the universal conservation of mt-GluRS and mt-AspRS in sponges (fig. 4), the finding of GatCAB homologs in some species that have lost corresponding mt-tRNA genes is a strong indication that these mt-aaRS may have been retained for the production of mis-charged tRNA intermediates, which are later modified through an indirect aminoacyl-tRNA transamidation pathway. Indeed, the retention of these mt-aaRS would be required for the mis-aminoacylation of imported cy-tRNAs if the corresponding cy-aaRS is not also imported into the mitochondrion, as is the case for GlnRS in human (Nagao et al. 2009). Interestingly, however, mt-AspRS and mt-GluRS are both retained in a group of sponges (Keratosa), where mt-tRNA substrates of both the direct and indirect pathways have been lost, implying additional functions for these enzymes. One possibility is that, prior to the loss of mt-tRNAs, these enzymes were recruited for the import of nuclear-encoded tRNAs into the mitochondrion using a mechanism similar to that described for the coimport of mt-LysRS and tRNA^Lys^ in yeast (Entelis and Kieffer 1998).

### Replacement of mt-tRNA^Ile^ Lysidine Synthetase

We identified a homolog of tRNA^Ile^ lysidine synthetase (TilS) in the placozoan *T. adhaerens,* representatives of all classes of sponges, as well as all unicellular relatives of animals used in this study (fig. 6supplementary fig. S1*W*, Supplementary Material online). In contrast, found no TilS homolog in any representatives of Cnidaria or Ctenophora, two animal phyla that have lost *trnI(cau),* the mitochondrial gene encoding the tRNA^Ile^(CAU) substrate for TilS. In addition, TilS homologs were absent in data sets from three sponges: The demosponges *I. fasciculata* and *Cr. elegans,* and the calcaronean sponge *Sy. coactum*. The lack of TilS in *I. fasciculata* is consistent with the lack of *trnI(cau)* in keratose sponges (Erpenbeck et al. 2009, fig. 1), and likely represents another example of the loss of this pathway in animals. In contrast, the lack of TilS in *Cr. elegans* is puzzling, considering that we identified a full set of tRNA genes in its mtDNA and that analysis using GenDecoder (Abascal et al. 2006) confirmed the isoleucine identity of AUA codons (AUA → Ile 80%, entropy < 1.0, gaps < 20%). Unless the result is a technical artifact, one possibility is that translation of AUA as isoleucine is achieved using tRNA^Ile^(ΨAU) imported from the cytosol, whereas mt-tRNA^Ile^(CAU) is retained for other functions or recruited for translation of methionine codons (e.g., Lavrov and Lang 2005). Finally, we could not interpret the lack of TilS in *Sy. coactum* because of a lack of mtDNA data from this species.

Surprisingly, we found a TilS homolog in *Ap. vastus*, a glass sponge that encodes a single mt-tRNA gene with the CAU anticodon that was previously identified as *trnM(cau)* (Haen et al. 2007). Although, this sequence lacks the R11–Y24 base pair that is typical of initiator tRNAs in other organisms, it groups with other sponge mt-tRNA^Met^ rather than mt-tRNA^Ile^ sequences (supplementary fig. S4, Supplementary Material online). The presence of TilS in *Ap. vastus* may thus suggest that mt-tRNA^Met^ in this species was recruited for the translation of AUA codons as isoleucine, a process previously observed in some demosponges (e.g., Lavrov and Lang 2005), whereas the gene encoding nuclear-encoded tRNA^Met^ is imported into the mitochondrion for the translation of methionine codons. Additional support for this hypothesis comes from the lack of a recognizable mt-MetRS homolog in this species, suggesting that cytosolic MetRS is imported for use in mitochondrial protein synthesis.

### Loss of mtRNaseP in Ctenophora

In humans, mtRNaseP consists of three protein subunits, two of which, MRPP1 and MRPP2, are members of large and diverse protein families, whereas the third, MRPP3, is thought to be a single copy gene in animals (Holzmann et al. 2008). In plant mitochondria, a single protein homologous to MRPP3 (PRORP1) has been shown to be responsible for organellar RNaseP activity (Gobert et al. 2010; Reinhard et al. 2015). Therefore MRPP3 can be considered a signature protein for this mt-tRNA processing activity. We identified homologs of MRPP3 in nearly all animal data sets we examined, plus unicellular relatives of animals, supporting the previous finding that this enzyme originated before the divergence of Holozoa (fig. 6). The only exception was Ctenophora, where no homologs of MRPP3 were identified in any of the data sets from the nine species that we examined (two complete genomes, nine transcriptomes). Interestingly, Ctenophora is the only group of animals where all mt-tRNAs have been lost (Pett et al. 2011; a report of two tRNA genes in *Pl. bachei* [Kohn et al. 2012] is likely due to misannotation [DL unpublished data]). We therefore conclude that the loss of mt-tRNAs in ctenophores rendered mtRNaseP activity superfluous, leading to its loss.

## Conclusions

The evolution of mitochondrial information processing pathways is characterized by recurrent replacement of mitochondrial components by their nuclear-encoded analogs. Although the major components of mitochondrial replication and transcription appear to have been substituted early in eukaryotic history, mitochondrial protein synthesis has continued to undergo a process of gradual replacement. Our results show that this process is in large part directed by the mt-tRNA complement of the mitochondrial genome, which varies substantially among eukaryotes, including animals. Although our previous studies in Cnidaria and Ctenophora suggested a fairly simple correlation between the presence of mt-tRNA genes and their associated processing activities, our expanded results from other nonbilaterian taxa revealed a more nuanced relationship. More data from underrepresented animal lineages that have lost mt-tRNAs, including the bilaterian phylum Chaetognatha (Faure and Casanova 2006), and some additional groups of sponges will help to shed more light on the dynamic evolution of mitochondrial translation.

## Supplementary Material

Supplementary figures S1–S4 are available at *Genome Biology and Evolution* online (http://www.gbe.oxfordjournals.org/).

Supplementary Data
