# Supplementary material for: Cytonuclear Interactions in the Evolution of Animal Mitochondrial tRNA Metabolism
Source: Genome Biol Evol. 2015 Jun 27;7(8):2089–101. doi: 10.1093/gbe/evv124 (PMC4558845; doi:10.1093/gbe/evv124)
Supplement: Supplementary Data [file supp_7_8_2089__index.html]

Cytonuclear Interactions in the Evolution of Animal Mitochondrial tRNA Metabolism — Supplementary Data 

# Cytonuclear Interactions in the Evolution of Animal Mitochondrial tRNA Metabolism

## Supplementary Data

files

- Supplementary Data - zip file
