# Supplementary material for: Cytonuclear Interactions in the Evolution of Animal Mitochondrial tRNA Metabolism
Source: Genome Biol Evol. 2015 Jun 27;7(8):2089–101. doi: 10.1093/gbe/evv124 (PMC4558845; doi:10.1093/gbe/evv124)

Supplementary Figure S1. Maximum likelihood phylogenetic trees used in profiling step
Trees were estimated using PhyML 3.0 with the WAG+Γ amino acid substitution model, and using both NNI and SPR rearrangements. Colored lineages were used by pplacer for the purpose of identifying putatively eukaryotic sequences during the profiling step. Sequences placed on blue lineages were inferred to be cytosolic in origin, sequenced placed on red lineages were inferred to be mitochondrial in origin, and sequence placed on purple lineages were inferred to be of dual-functional or unknown origin. **A.** AlaRS, **B.** CysRS, **C.** AspRS, **D.** Glu/GluPro/GlnRS, **E.** PheRS-α/mt-PheRS, **F.** PheRS-β/mt-PheRS, **G.** GlyRS, **H.** HisRS, **I.** IleRS, **J.** LysRS, **K.** LeuRS, **L.** MetRS, **M.** AsnRS, **N.** Pro/GluProRS, **O.** ArgRS, **P.** SerRS, **Q.** ThrRS, **R.** ValRS, **S.** TrpRS, **T.** TyrRS, **U.** GatA, **V.** GatB, **W.** TilS

A. AlaRS

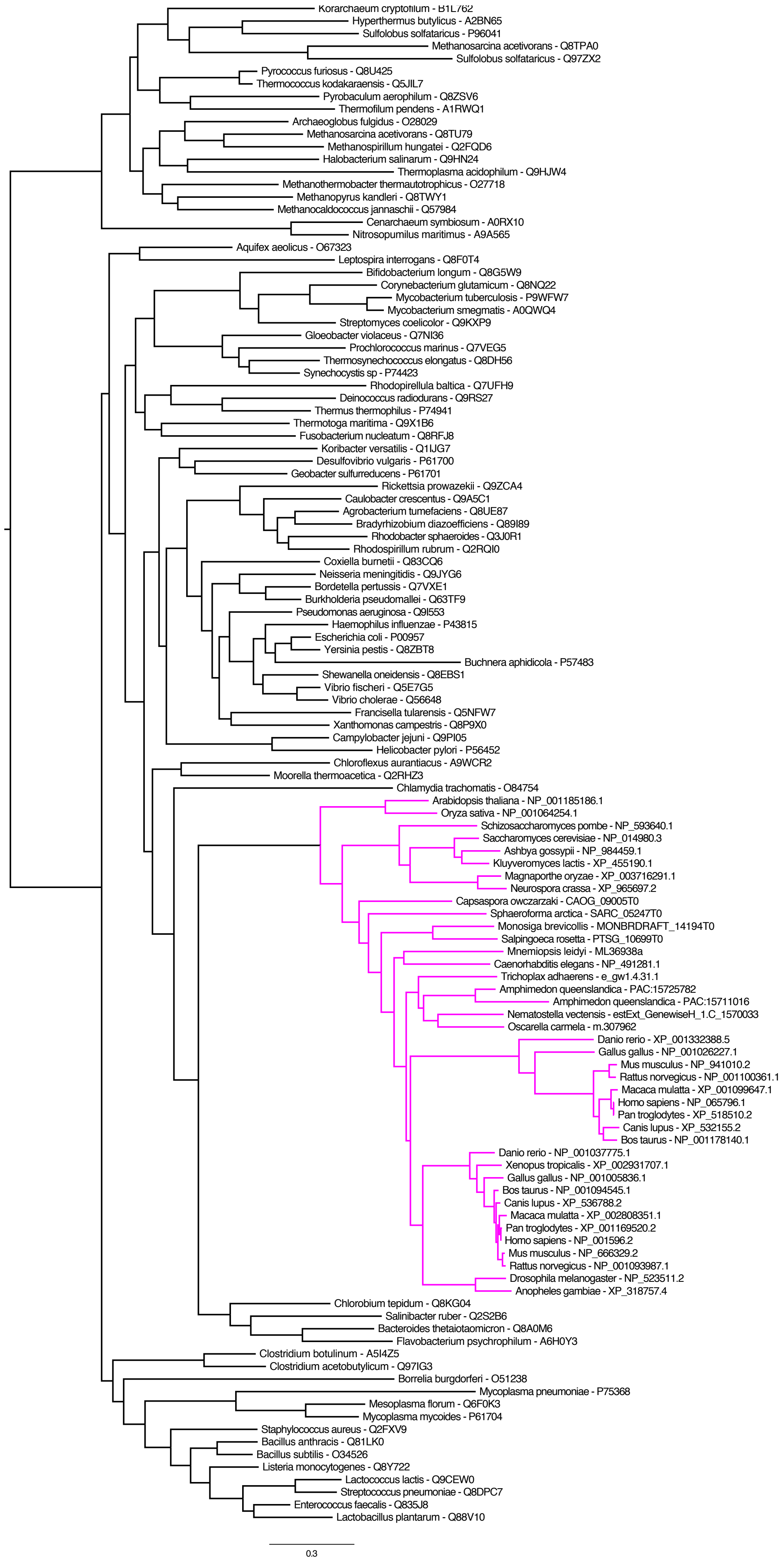

Korarchaeum cryptofilum - B1L762
Hyperthermus butylicus - A2BN65
Sulfolobus solfataricus - P96041
Methanosarcina acetivorans - Q8TPA0
Sulfolobus solfataricus - Q97ZX2
Pyrococcus furiosus - Q8U425
Thermococcus kodakaraensis - Q5JIL7
Pyrobaculum aerophilum - Q8ZSV6
Thermofilum pendens - A1RWQ1
Archaeoglobus fulgidus - O28029
Methanosarcina acetivorans - Q8TU79
Methanospirillum hungatei - Q2FQD6
Halobacterium salinarum - Q9HN24
Thermoplasma acidophilum - Q9HJW4
Methanothermobacter thermautotrophicus - O27718
Methanopyrus kandleri - Q8TWY1
Methanocaldococcus jannaschii - Q57984
Cenarchaeum symbiosum - A0RX10
Nitrosopumilus maritimus - A9A565
Aquifex aeolicus - O67323
Leptospira interrogans - Q8F0T4
Bifidobacterium longum - Q8G5W9
Corynebacterium glutamicum - Q8NQ22
Mycobacterium tuberculosis - P9WFW7
Mycobacterium smegmatis - A0QWQ4
Streptomyces coelicolor - Q9KXP9
Gloeobacter violaceus - Q7NI36
Prochlorococcus marinus - Q7VEG5
Thermosynechococcus elongatus - Q8DH56
Synechocystis sp - P74423
Rhodopirellula baltica - Q7UFH9
Deinococcus radiodurans - Q9RS27
Thermus thermophilus - P74941
Thermotoga maritima - Q9X1B6
Fusobacterium nucleatum - Q8RFJ8
Koribacter versatilis - Q1IJG7
Desulfovibrio vulgaris - P61700
Geobacter sulfurreducens - P61701
Rickettsia prowazekii - Q9ZCA4
Caulobacter crescentus - Q9A5C1
Agrobacterium tumefaciens - Q8UE87
Bradyrhizobium diazoefficiens - Q89I89
Rhodobacter sphaeroides - Q3J0R1
Rhodospirillum rubrum - Q2RQI0
Coxiella burnetii - Q83CQ6
Neisseria meningitidis - Q9JYG6
Bordetella pertussis - Q7VXE1
Burkholderia pseudomallei - Q63TF9
Pseudomonas aeruginosa - Q9I553
Haemophilus influenzae - P43815
Escherichia coli - P00957
Yersinia pestis - Q8ZBT8
Buchnera aphidicola - P57483
Shewanella oneidensis - Q8EBS1
Vibrio fischeri - Q5E7G5
Vibrio cholerae - Q56648
Francisella tularensis - Q5NFW7
Xanthomonas campestris - Q8P9X0
Campylobacter jejuni - Q9PI05
Helicobacter pylori - P56452
Chloroflexus aurantiacus - A9WCR2
Moorella thermoacetica - Q2RHZ3
Chlamydia trachomatis - O84754
Arabidopsis thaliana - NP_001185186.1
Oryza sativa - NP_001064254.1
Schizosaccharomyces pombe - NP_593640.1
Saccharomyces cerevisiae - NP_014980.3
Ashbya gossypii - NP_984459.1
Kluyveromyces lactis - XP_455190.1
Magnaporthe oryzae - XP_003716291.1
Neurospora crassa - XP_965697.2
Capsaspora owczarzaki - CAOG_09005T0
Sphaeroforma arctica - SARC_05247T0
Monosiga brevicollis - MONBRDRAFT_14194T0
Salpingoeca rosetta - PTSG_10699T0
Mnemiopsis leidyi - ML36938a
Caenorhabditis elegans - NP_491281.1
Trichoplax adhaerens - e_gw1.4.31.1
Amphimedon queenslandica - PAC:15725782
Amphimedon queenslandica - PAC:15711016
Nematostella vectensis - estExt_GenewiseH_1.C_1570033
Oscarella carmela - m.307962
Danio rerio - XP_001332388.5
Gallus gallus - NP_001026227.1
Mus musculus - NP_941010.2
Rattus norvegicus - NP_001100361.1
Macaca mulatta - XP_001099647.1
Homo sapiens - NP_065796.1
Pan troglodytes - XP_518510.2
Canis lupus - XP_532155.2
Bos taurus - NP_001178140.1
Danio rerio - NP_001037775.1
Xenopus tropicalis - XP_002931707.1
Gallus gallus - NP_001005836.1
Bos taurus - NP_001094545.1
Canis lupus - XP_536788.2
Macaca mulatta - XP_002808351.1
Pan troglodytes - XP_001169520.2
Homo sapiens - NP_001596.2
Mus musculus - NP_666329.2
Rattus norvegicus - NP_001093987.1
Drosophila melanogaster - NP_523511.2
Anopheles gambiae - XP_318757.4
Chlorobium tepidum - Q8KG04
Salinibacter ruber - Q2S2B6
Bacteroides thetaiotaomicron - Q8A0M6
Flavobacterium psychrophilum - A6H0Y3
Clostridium botulinum - A5I4Z5
Clostridium acetobutylicum - Q97IG3
Borrelia burgdorferi - O51238
Mycoplasma pneumoniae - P75368
Mesoplasma florum - Q6F0K3
Mycoplasma mycoides - P61704
Staphylococcus aureus - Q2FXV9
Bacillus anthracis - Q81LK0
Bacillus subtilis - O34526
Listeria monocytogenes - Q8Y722
Lactococcus lactis - Q9CEW0
Streptococcus pneumoniae - Q8DPC7
Enterococcus faecalis - Q835J8
Lactobacillus plantarum - Q88V10
0.3

B. CysRS

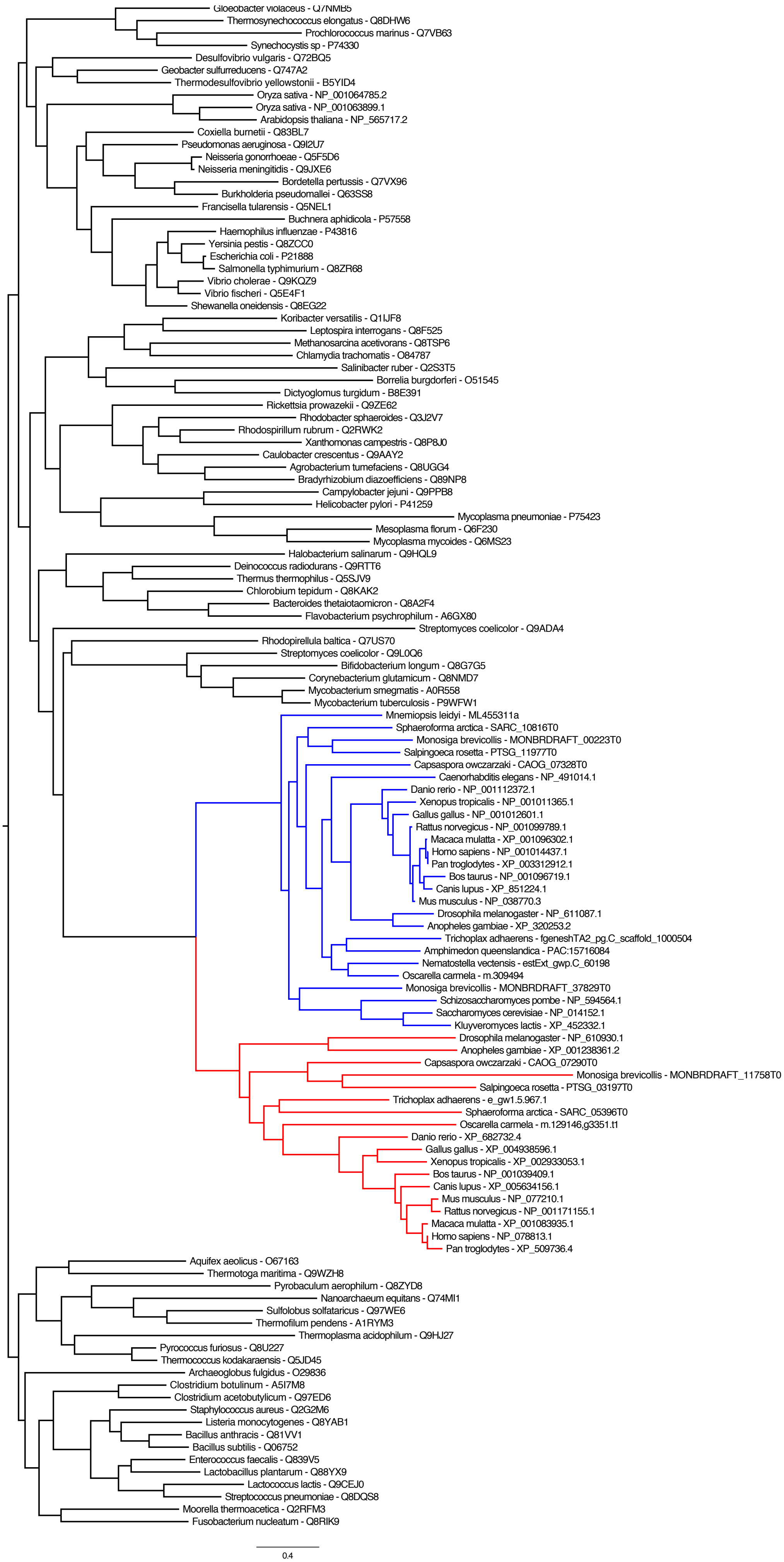

Gloeobacter violaceus - Q7NMB5
Thermosynechococcus elongatus - Q8DHW6
Prochlorococcus marinus - Q7VB63
Synechocystis sp - P74330
Desulfovibrio vulgaris - Q72BQ5
Geobacter sulfurreducens - Q747A2
Thermodesulfovibrio yellowstonii - B5YID4
Oryza sativa - NP_001064785.2
Oryza sativa - NP_001063899.1
Arabidopsis thaliana - NP_565717.2
Coxiella burnetii - Q83BL7
Pseudomonas aeruginosa - Q9I2U7
Neisseria gonorrhoeae - Q5F5D6
Neisseria meningitidis - Q9JXE6
Bordetella pertussis - Q7VX96
Burkholderia pseudomallei - Q63SS8
Francisella tularensis - Q5NEL1
Buchnera aphidicola - P57558
Haemophilus influenzae - P43816
Yersinia pestis - Q8ZCC0
Escherichia coli - P21888
Salmonella typhimurium - Q8ZR68
Vibrio cholerae - Q9KQZ9
Vibrio fischeri - Q5E4F1
Shewanella oneidensis - Q8EG22
Koribacter versatilis - Q1IJF8
Leptospira interrogans - Q8F525
Methanosarcina acetivorans - Q8TSP6
Chlamydia trachomatis - O84787
Salinibacter ruber - Q2S3T5
Borrelia burgdorferi - O51545
Dictyoglomus turgidum - B8E391
Rickettsia prowazekii - Q9ZE62
Rhodobacter sphaeroides - Q3J2V7
Rhodospirillum rubrum - Q2RWK2
Xanthomonas campestris - Q8P8J0
Caulobacter crescentus - Q9AAY2
Agrobacterium tumefaciens - Q8UGG4
Bradyrhizobium diazoefficiens - Q89NP8
Campylobacter jejuni - Q9PPB8
Helicobacter pylori - P41259
Mycoplasma pneumoniae - P75423
Mesoplasma florum - Q6F230
Mycoplasma mycoides - Q6MS23
Halobacterium salinarum - Q9HQL9
Deinococcus radiodurans - Q9RTT6
Thermus thermophilus - Q5SJV9
Chlorobium tepidum - Q8KAK2
Bacteroides thetaiotaomicron - Q8A2F4
Flavobacterium psychrophilum - A6GX80
Streptomyces coelicolor - Q9ADA4
Rhodopirellula baltica - Q7US70
Streptomyces coelicolor - Q9L0Q6
Bifidobacterium longum - Q8G7G5
Corynebacterium glutamicum - Q8NMD7
Mycobacterium smegmatis - A0R558
Mycobacterium tuberculosis - P9WFW1
Mnemiopsis leidyi - ML455311a
Sphaeroforma arctica - SARC_10816T0
Monosiga brevicollis - MONBRDRAFT_00223T0
Salpingoeca rosetta - PTSG_11977T0
Capsaspora owczarzaki - CAOG_07328T0
Caenorhabditis elegans - NP_491014.1
Danio rerio - NP_001112372.1
Xenopus tropicalis - NP_001011365.1
Gallus gallus - NP_001012601.1
Rattus norvegicus - NP_001099789.1
Macaca mulatta - XP_001096302.1
Homo sapiens - NP_001014437.1
Pan troglodytes - XP_003312912.1
Bos taurus - NP_001096719.1
Canis lupus - XP_851224.1
Mus musculus - NP_038770.3
Drosophila melanogaster - NP_611087.1
Anopheles gambiae - XP_320253.2
Trichoplax adhaerens - fgeneshTA2_pg.C_scaffold_1000504
Amphimedon queenslandica - PAC:15716084
Nematostella vectensis - estExt_gwp.C_60198
Oscarella carmela - m.309494
Monosiga brevicollis - MONBRDRAFT_37829T0
Schizosaccharomyces pombe - NP_594564.1
Saccharomyces cerevisiae - NP_014152.1
Kluyveromyces lactis - XP_452332.1
Drosophila melanogaster - NP_610930.1
Anopheles gambiae - XP_001238361.2
Capsaspora owczarzaki - CAOG_07290T0
Monosiga brevicollis - MONBRDRAFT_11758T0
Salpingoeca rosetta - PTSG_03197T0
Trichoplax adhaerens - e_gw1.5.967.1
Sphaeroforma arctica - SARC_05396T0
Oscarella carmela - m.129146,g3351.t1
Danio rerio - XP_682732.4
Gallus gallus - XP_004938596.1
Xenopus tropicalis - XP_002933053.1
Bos taurus - NP_001039409.1
Canis lupus - XP_005634156.1
Mus musculus - NP_077210.1
Rattus norvegicus - NP_001171155.1
Macaca mulatta - XP_001083935.1
Homo sapiens - NP_078813.1
Pan troglodytes - XP_509736.4
Aquifex aeolicus - O67163
Thermotoga maritima - Q9WZH8
Pyrobaculum aerophilum - Q8ZYD8
Nanoarchaeum equitans - Q74MI1
Sulfolobus solfataricus - Q97WE6
Thermofilum pendens - A1RYM3
Thermoplasma acidophilum - Q9HJ27
Pyrococcus furiosus - Q8U227
Thermococcus kodakaraensis - Q5JD45
Archaeoglobus fulgidus - O29836
Clostridium botulinum - A5I7M8
Clostridium acetobutylicum - Q97ED6
Staphylococcus aureus - Q2G2M6
Listeria monocytogenes - Q8YAB1
Bacillus anthracis - Q81VV1
Bacillus subtilis - Q06752
Enterococcus faecalis - Q839V5
Lactobacillus plantarum - Q88YX9
Lactococcus lactis - Q9CEJ0
Streptococcus pneumoniae - Q8DQS8
Moorella thermoacetica - Q2RFM3
Fusobacterium nucleatum - Q8RIK9
0.4

C. AspRS

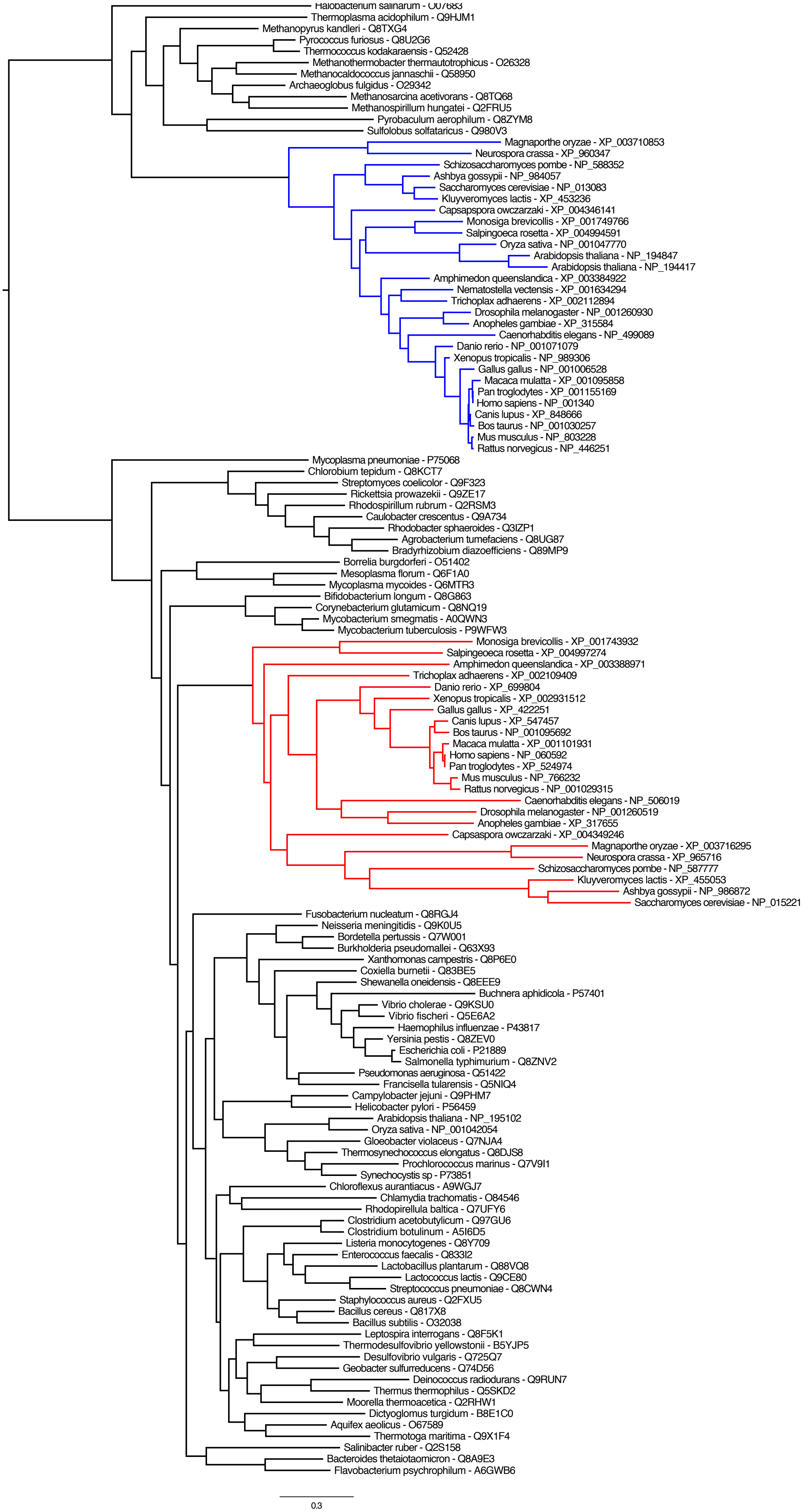

Halobacterium salinarum - O07683
Thermoplasma acidophilum - Q9HJM1
Methanopyrus kandleri - Q8TXG4
Pyrococcus furiosus - Q8U2G6
Thermococcus kodakaraensis - Q52428
Methanothermobacter thermautotrophicus - O26328
Methanocaldococcus jannaschii - Q58950
Archaeoglobus fulgidus - O29342
Methanosarcina acetivorans - Q8TQ68
Methanospirillum hungatei - Q2FRU5
Pyrobaculum aerophilum - Q8ZYM8
Sulfolobus solfataricus - Q980V3
Magnaporthe oryzae - XP_003710853
Neurospora crassa - XP_960347
Schizosaccharomyces pombe - NP_588352
Ashbya gossypii - NP_984057
Saccharomyces cerevisiae - NP_013083
Kluyveromyces lactis - XP_453236
Capsaspora owczarzaki - XP_004346141
Monosiga brevicollis - XP_001749766
Salpingoeca rosetta - XP_004994591
Oryza sativa - NP_001047770
Arabidopsis thaliana - NP_194847
Arabidopsis thaliana - NP_194417
Amphimedon queenslandica - XP_003384922
Nematostella vectensis - XP_001634294
Trichoplax adhaerens - XP_002112894
Drosophila melanogaster - NP_001260930
Anopheles gambiae - XP_315584
Caenorhabditis elegans - NP_499089
Danio rerio - NP_001071079
Xenopus tropicalis - NP_989306
Gallus gallus - NP_001006528
Macaca mulatta - XP_001095858
Pan troglodytes - XP_001155169
Homo sapiens - NP_001340
Canis lupus - XP_848666
Bos taurus - NP_001030257
Mus musculus - NP_803228
Rattus norvegicus - NP_446251
Mycoplasma pneumoniae - P75068
Chlorobium tepidum - Q8KCT7
Streptomyces coelicolor - Q9F323
Rickettsia prowazekii - Q9ZE17
Rhodospirillum rubrum - Q2RSM3
Caulobacter crescentus - Q9A734
Rhodobacter sphaeroides - Q3IZP1
Agrobacterium tumefaciens - Q8UG87
Bradyrhizobium diazoefficiens - Q89MP9
Borrelia burgdorferi - O51402
Mesoplasma florum - Q6F1A0
Mycoplasma mycoides - Q6MTR3
Bifidobacterium longum - Q8G863
Corynebacterium glutamicum - Q8NQ19
Mycobacterium smegmatis - A0QWN3
Mycobacterium tuberculosis - P9WFW3
Monosiga brevicollis - XP_001743932
Salpingeoca rosetta - XP_004997274
Amphimedon queenslandica - XP_003388971
Trichoplax adhaerens - XP_002109409
Danio rerio - XP_699804
Xenopus tropicalis - XP_002931512
Gallus gallus - XP_422251
Canis lupus - XP_547457
Bos taurus - NP_001095692
Macaca mulatta - XP_001101931
Homo sapiens - NP_060592
Pan troglodytes - XP_524974
Mus musculus - NP_766232
Rattus norvegicus - NP_001029315
Caenorhabditis elegans - NP_506019
Drosophila melanogaster - NP_001260519
Anopheles gambiae - XP_317655
Capsaspora owczarzaki - XP_004349246
Magnaporthe oryzae - XP_003716295
Neurospora crassa - XP_965716
Schizosaccharomyces pombe - NP_587777
Kluyveromyces lactis - XP_455053
Ashbya gossypii - NP_986872
Saccharomyces cerevisiae - NP_015221
Fusobacterium nucleatum - Q8RGJ4
Neisseria meningitidis - Q9K0U5
Bordetella pertussis - Q7W001
Burkholderia pseudomallei - Q63X93
Xanthomonas campestris - Q8P6E0
Coxiella burnetii - Q83BE5
Shewanella oneidensis - Q8EEE9
Buchnera aphidicola - P57401
Vibrio cholerae - Q9KSU0
Vibrio fischeri - Q5E6A2
Haemophilus influenzae - P43817
Yersinia pestis - Q8ZEV0
Escherichia coli - P21889
Salmonella typhimurium - Q8ZNV2
Pseudomonas aeruginosa - Q51422
Francisella tularensis - Q5NIQ4
Campylobacter jejuni - Q9PHM7
Helicobacter pylori - P56459
Arabidopsis thaliana - NP_195102
Oryza sativa - NP_001042054
Gloeobacter violaceus - Q7NJA4
Thermosynechococcus elongatus - Q8DJS8
Prochlorococcus marinus - Q7V9I1
Synechocystis sp - P73851
Chloroflexus aurantiacus - A9WGJ7
Chlamydia trachomatis - O84546
Rhodopirellula baltica - Q7UFY6
Clostridium acetobutylicum - Q97GU6
Clostridium botulinum - A5I6D5
Listeria monocytogenes - Q8Y709
Enterococcus faecalis - Q833I2
Lactobacillus plantarum - Q88VQ8
Lactococcus lactis - Q9CE80
Streptococcus pneumoniae - Q8CWN4
Staphylococcus aureus - Q2FXU5
Bacillus cereus - Q817X8
Bacillus subtilis - O32038
Leptospira interrogans - Q8F5K1
Thermodesulfovibrio yellowstonii - B5YJP5
Desulfovibrio vulgaris - Q725Q7
Geobacter sulfurreducens - Q74D56
Deinococcus radiodurans - Q9RUN7
Thermus thermophilus - Q5SKD2
Moorella thermoacetica - Q2RHW1
Dictyoglomus turgidum - B8E1C0
Aquifex aeolicus - O67589
Thermotoga maritima - Q9X1F4
Salinibacter ruber - Q2S158
Bacteroides thetaiotaomicron - Q8A9E3
Flavobacterium psychrophilum - A6GWB6
0.3

D. Glu/GluPro/GlnRS

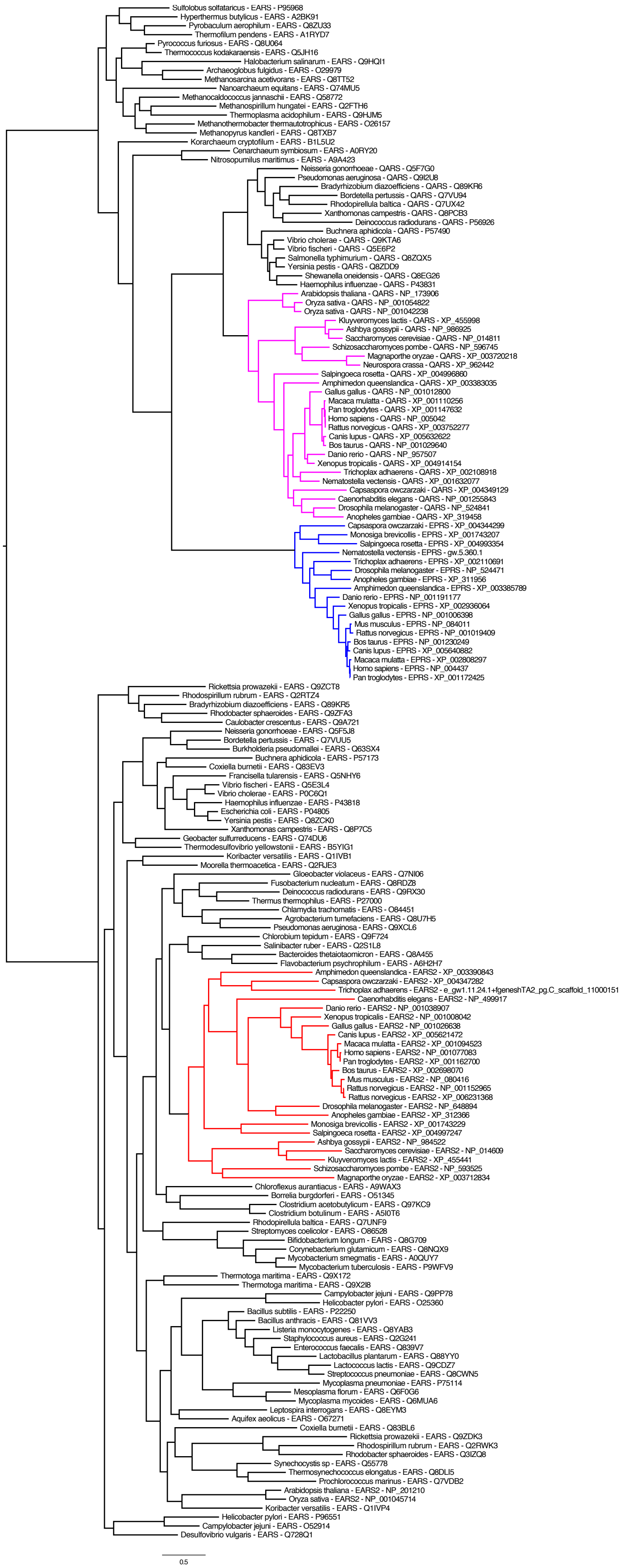

E. PheRS-α / mt-PheRS

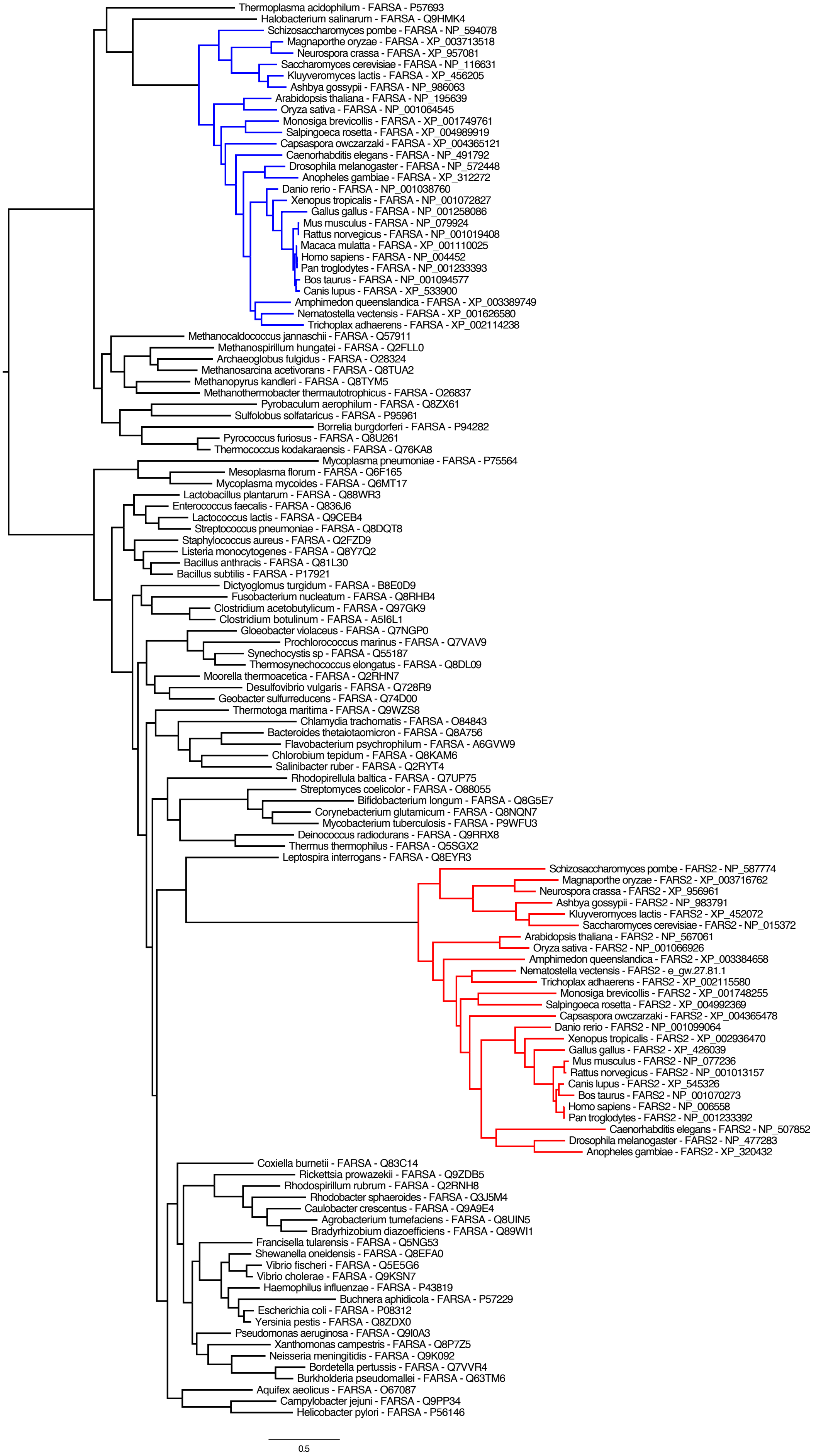

F. PheRS-β / mt-PheRS

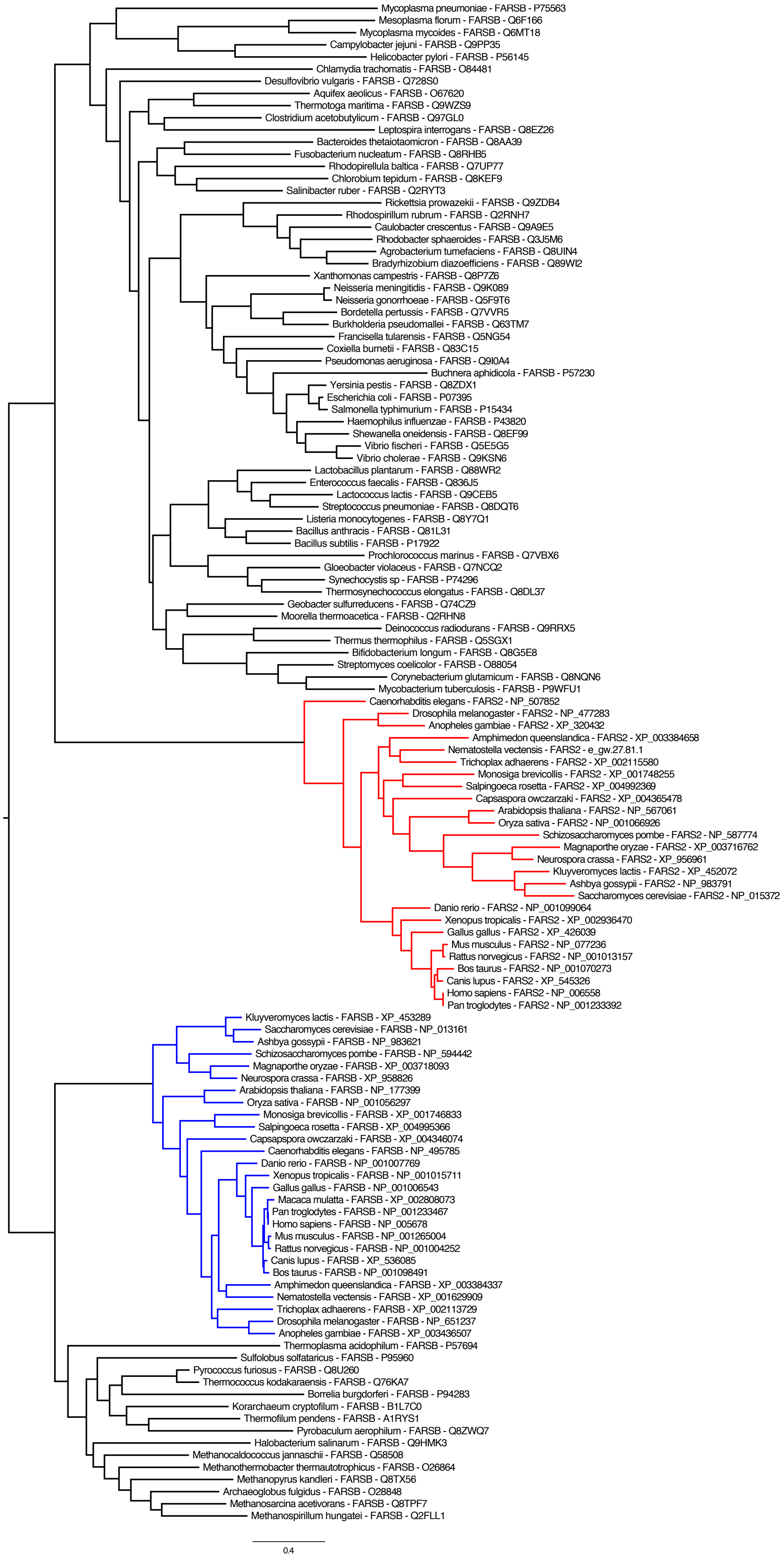

G. GlyRS

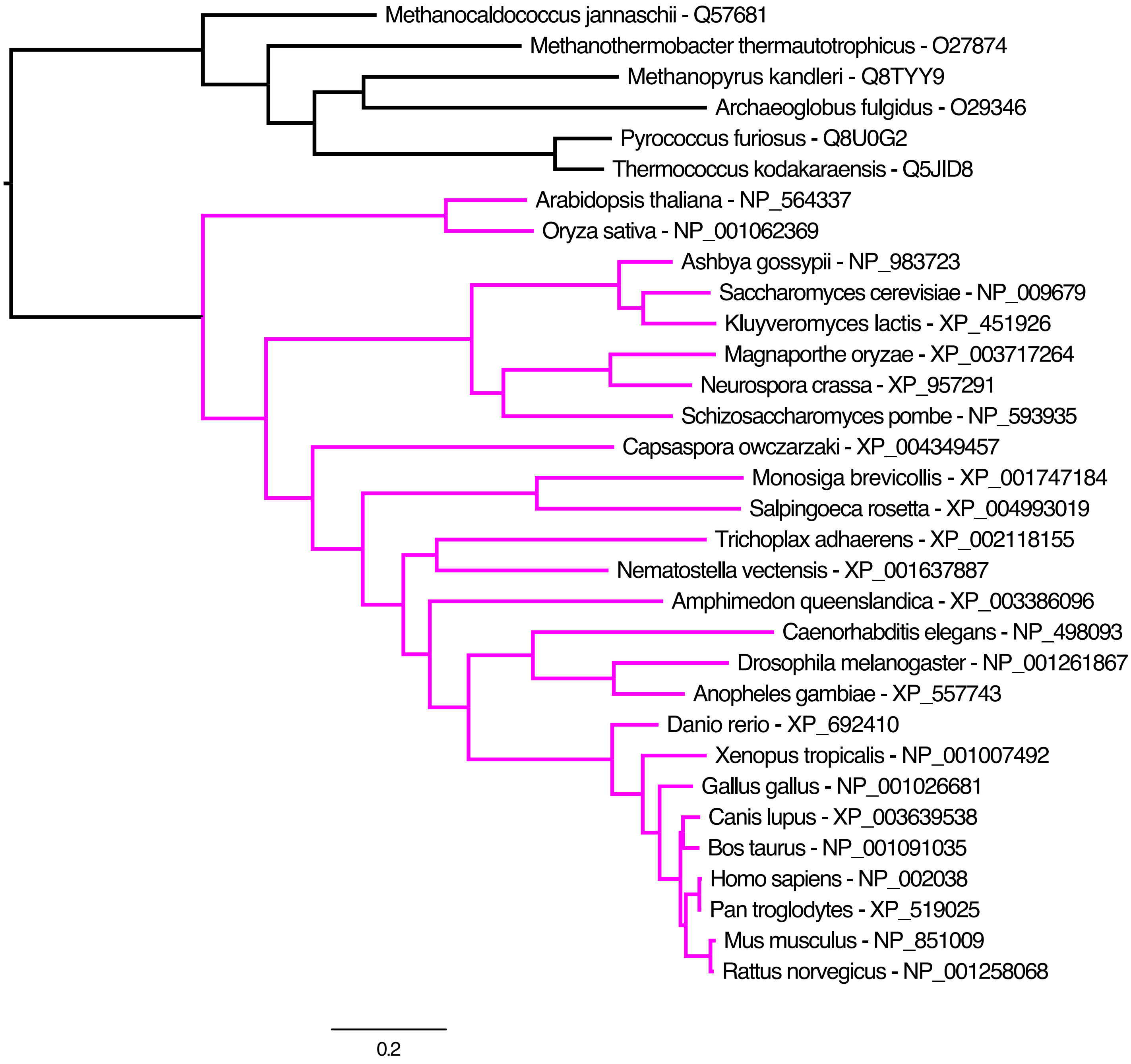

Methanocaldococcus jannaschii - Q57681
Methanothermobacter thermautotrophicus - O27874
Methanopyrus kandleri - Q8TYY9
Archaeoglobus fulgidus - O29346
Pyrococcus furiosus - Q8U0G2
Thermococcus kodakaraensis - Q5JID8
Arabidopsis thaliana - NP_564337
Oryza sativa - NP_001062369
Ashbya gossypii - NP_983723
Saccharomyces cerevisiae - NP_009679
Kluyveromyces lactis - XP_451926
Magnaporthe oryzae - XP_003717264
Neurospora crassa - XP_957291
Schizosaccharomyces pombe - NP_593935
Capsaspora owczarzaki - XP_004349457
Monosiga brevicollis - XP_001747184
Salpingoeca rosetta - XP_004993019
Trichoplax adhaerens - XP_002118155
Nematostella vectensis - XP_001637887
Amphimedon queenslandica - XP_003386096
Caenorhabditis elegans - NP_498093
Drosophila melanogaster - NP_001261867
Anopheles gambiae - XP_557743
Danio rerio - XP_692410
Xenopus tropicalis - NP_001007492
Gallus gallus - NP_001026681
Canis lupus - XP_003639538
Bos taurus - NP_001091035
Homo sapiens - NP_002038
Pan troglodytes - XP_519025
Mus musculus - NP_851009
Rattus norvegicus - NP_001258068
0.2

H. HisRS

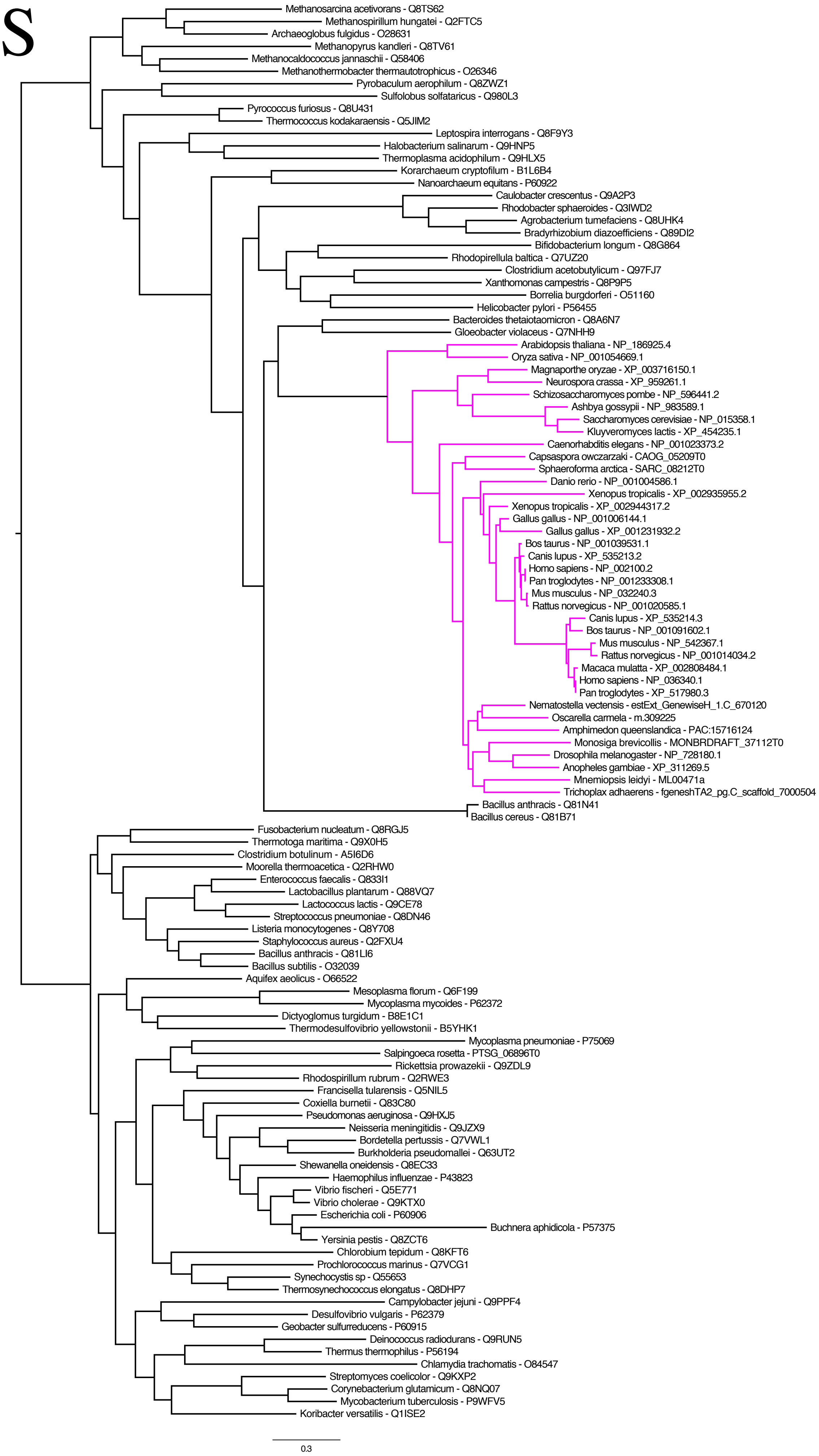

Methanosarcina acetivorans - Q8TS62
Methanospirillum hungatei - Q2FTC5
Archaeoglobus fulgidus - O28631
Methanopyrus kandleri - Q8TV61
Methanocaldococcus jannaschii - Q58406
Methanothermobacter thermautotrophicus - O26346
Pyrobaculum aerophilum - Q8ZWZ1
Sulfolobus solfataricus - Q980L3
Pyrococcus furiosus - Q8U431
Thermococcus kodakaraensis - Q5JIM2
Leptospira interrogans - Q8F9Y3
Halobacterium salinarum - Q9HNP5
Thermoplasma acidophilum - Q9HLX5
Korarchaeum cryptofilum - B1L6B4
Nanoarchaeum equitans - P60922
Caulobacter crescentus - Q9A2P3
Rhodobacter sphaeroides - Q3IWD2
Agrobacterium tumefaciens - Q8UHK4
Bradyrhizobium diazoefficiens - Q89DI2
Bifidobacterium longum - Q8G864
Rhodopirellula baltica - Q7UZ20
Clostridium acetobutylicum - Q97FJ7
Xanthomonas campestris - Q8P9P5
Borrelia burgdorferi - O51160
Helicobacter pylori - P56455
Bacteroides thetaiotaomicron - Q8A6N7
Gloeobacter violaceus - Q7NHH9
Arabidopsis thaliana - NP_186925.4
Oryza sativa - NP_001054669.1
Magnaporthe oryzae - XP_003716150.1
Neurospora crassa - XP_959261.1
Schizosaccharomyces pombe - NP_596441.2
Ashbya gossypii - NP_983589.1
Saccharomyces cerevisiae - NP_015358.1
Kluyveromyces lactis - XP_454235.1
Caenorhabditis elegans - NP_001023373.2
Capsaspora owczarzaki - CAOG_05209T0
Sphaeroforma arctica - SARC_08212T0
Danio rerio - NP_001004586.1
Xenopus tropicalis - XP_002935955.2
Xenopus tropicalis - XP_002944317.2
Gallus gallus - NP_001006144.1
Gallus gallus - XP_001231932.2
Bos taurus - NP_001039531.1
Canis lupus - XP_535213.2
Homo sapiens - NP_002100.2
Pan troglodytes - NP_001233308.1
Mus musculus - NP_032240.3
Rattus norvegicus - NP_001020585.1
Canis lupus - XP_535214.3
Bos taurus - NP_001091602.1
Mus musculus - NP_542367.1
Rattus norvegicus - NP_001014034.2
Macaca mulatta - XP_002808484.1
Homo sapiens - NP_036340.1
Pan troglodytes - XP_517980.3
Nematostella vectensis - estExt_GenewiseH_1.C_670120
Oscarella carmela - m.309225
Amphimedon queenslandica - PAC:15716124
Monosiga brevicollis - MONBRDRAFT_37112T0
Drosophila melanogaster - NP_728180.1
Anopheles gambiae - XP_311269.5
Mnemiopsis leidyi - ML00471a
Trichoplax adhaerens - fgeneshTA2_pg.C_scaffold_7000504
Bacillus anthracis - Q81N41
Bacillus cereus - Q81B71
Fusobacterium nucleatum - Q8RGJ5
Thermotoga maritima - Q9X0H5
Clostridium botulinum - A5I6D6
Moorella thermoacetica - Q2RHW0
Enterococcus faecalis - Q833I1
Lactobacillus plantarum - Q88VQ7
Lactococcus lactis - Q9CE78
Streptococcus pneumoniae - Q8DN46
Listeria monocytogenes - Q8Y708
Staphylococcus aureus - Q2FXU4
Bacillus anthracis - Q81LI6
Bacillus subtilis - O32039
Aquifex aeolicus - O66522
Mesoplasma florum - Q6F199
Mycoplasma mycoides - P62372
Dictyoglomus turgidum - B8E1C1
Thermodesulfovibrio yellowstonii - B5YHK1
Mycoplasma pneumoniae - P75069
Salpingoeca rosetta - PTSG_06896T0
Rickettsia prowazekii - Q9ZDL9
Rhodospirillum rubrum - Q2RWE3
Francisella tularensis - Q5NIL5
Coxiella burnetii - Q83C80
Pseudomonas aeruginosa - Q9HXJ5
Neisseria meningitidis - Q9JZX9
Bordetella pertussis - Q7VWL1
Burkholderia pseudomallei - Q63UT2
Shewanella oneidensis - Q8EC33
Haemophilus influenzae - P43823
Vibrio fischeri - Q5E771
Vibrio cholerae - Q9KTX0
Escherichia coli - P60906
Buchnera aphidicola - P57375
Yersinia pestis - Q8ZCT6
Chlorobium tepidum - Q8KFT6
Prochlorococcus marinus - Q7VCG1
Synechocystis sp - Q55653
Thermosynechococcus elongatus - Q8DHP7
Campylobacter jejuni - Q9PPF4
Desulfovibrio vulgaris - P62379
Geobacter sulfurreducens - P60915
Deinococcus radiodurans - Q9RUN5
Thermus thermophilus - P56194
Chlamydia trachomatis - O84547
Streptomyces coelicolor - Q9KXP2
Corynebacterium glutamicum - Q8NQ07
Mycobacterium tuberculosis - P9WFV5
Koribacter versatilis - Q1ISE2
0.3

I. IleRS

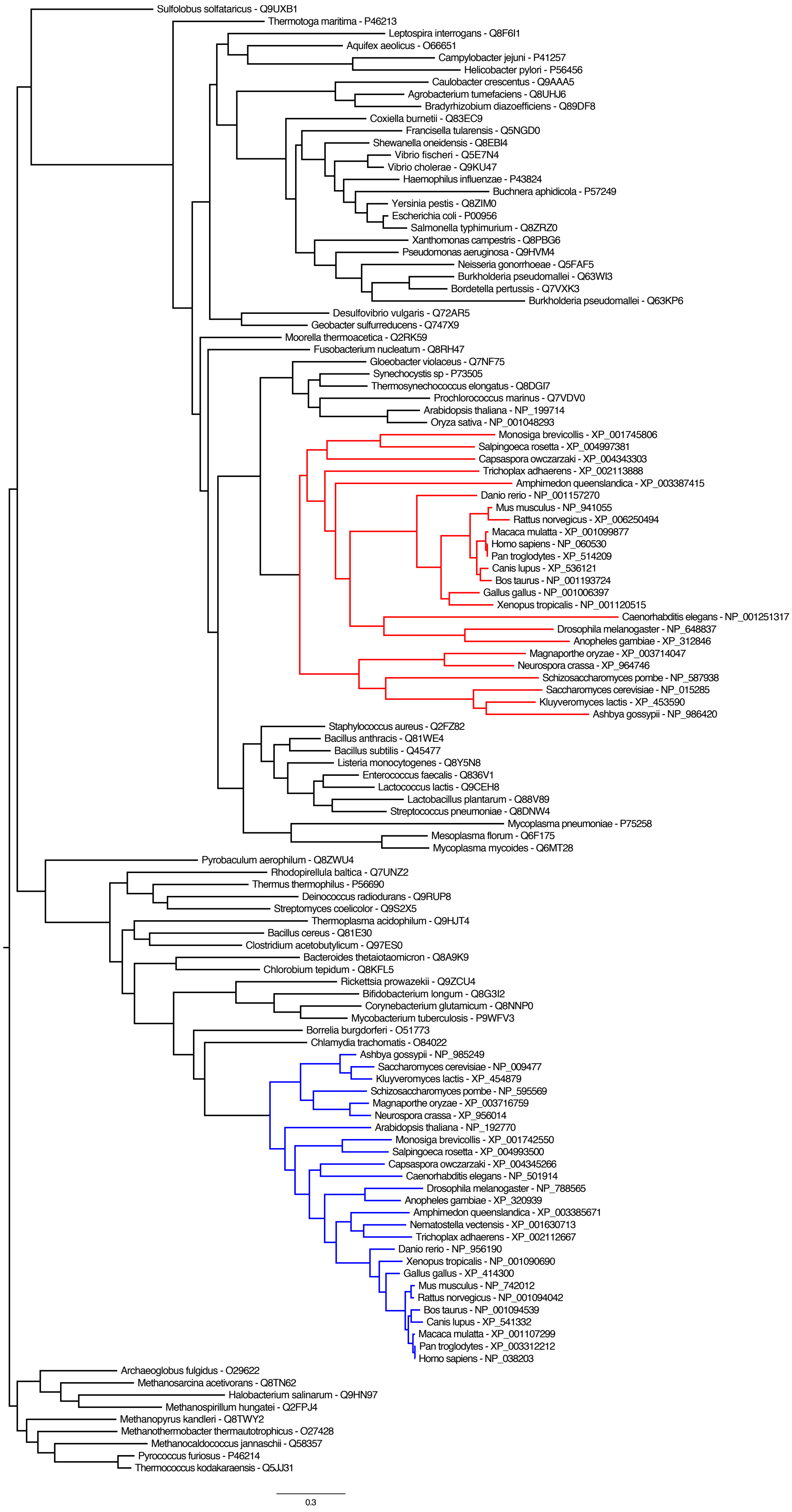

Sulfolobus solfataricus - Q9UXB1
Thermotoga maritima - P46213
Leptospira interrogans - Q8F6I1
Aquifex aeolicus - O66651
Campylobacter jejuni - P41257
Helicobacter pylori - P56456
Caulobacter crescentus - Q9AAA5
Agrobacterium tumefaciens - Q8UHJ6
Bradyrhizobium diazoefficiens - Q89DF8
Coxiella burnetii - Q83EC9
Francisella tularensis - Q5NGD0
Shewanella oneidensis - Q8EBI4
Vibrio fischeri - Q5E7N4
Vibrio cholerae - Q9KU47
Haemophilus influenzae - P43824
Buchnera aphidicola - P57249
Yersinia pestis - Q8ZIM0
Escherichia coli - P00956
Salmonella typhimurium - Q8ZRZ0
Xanthomonas campestris - Q8PBG6
Pseudomonas aeruginosa - Q9HVM4
Neisseria gonorrhoeae - Q5FAF5
Burkholderia pseudomallei - Q63WI3
Bordetella pertussis - Q7VXK3
Burkholderia pseudomallei - Q63KP6
Desulfovibrio vulgaris - Q72AR5
Geobacter sulfurreducens - Q747X9
Moorella thermoacetica - Q2RK59
Fusobacterium nucleatum - Q8RH47
Gloeobacter violaceus - Q7NF75
Synechocystis sp - P73505
Thermosynechococcus elongatus - Q8DGI7
Prochlorococcus marinus - Q7VDV0
Arabidopsis thaliana - NP_199714
Oryza sativa - NP_001048293
Monosiga brevicollis - XP_001745806
Salpingoeca rosetta - XP_004997381
Capsaspora owczarzaki - XP_004343303
Trichoplax adhaerens - XP_002113888
Amphimedon queenslandica - XP_003387415
Danio rerio - NP_001157270
Mus musculus - NP_941055
Rattus norvegicus - XP_006250494
Macaca mulatta - XP_001099877
Homo sapiens - NP_060530
Pan troglodytes - XP_514209
Canis lupus - XP_536121
Bos taurus - NP_001193724
Gallus gallus - NP_001006397
Xenopus tropicalis - NP_001120515
Caenorhabditis elegans - NP_001251317
Drosophila melanogaster - NP_648837
Anopheles gambiae - XP_312846
Magnaporthe oryzae - XP_003714047
Neurospora crassa - XP_964746
Schizosaccharomyces pombe - NP_587938
Saccharomyces cerevisiae - NP_015285
Kluyveromyces lactis - XP_453590
Ashbya gossypii - NP_986420
Staphylococcus aureus - Q2FZ82
Bacillus anthracis - Q81WE4
Bacillus subtilis - Q45477
Listeria monocytogenes - Q8Y5N8
Enterococcus faecalis - Q836V1
Lactococcus lactis - Q9CEH8
Lactobacillus plantarum - Q88V89
Streptococcus pneumoniae - Q8DNW4
Mycoplasma pneumoniae - P75258
Mesoplasma florum - Q6F175
Mycoplasma mycoides - Q6MT28
Pyrobaculum aerophilum - Q8ZWU4
Rhodopirellula baltica - Q7UNZ2
Thermus thermophilus - P56690
Deinococcus radiodurans - Q9RUP8
Streptomyces coelicolor - Q9S2X5
Thermoplasma acidophilum - Q9HJT4
Bacillus cereus - Q81E30
Clostridium acetobutylicum - Q97ES0
Bacteroides thetaiotaomicron - Q8A9K9
Chlorobium tepidum - Q8KFL5
Rickettsia prowazekii - Q9ZCU4
Bifidobacterium longum - Q8G3I2
Corynebacterium glutamicum - Q8NNP0
Mycobacterium tuberculosis - P9WFV3
Borrelia burgdorferi - O51773
Chlamydia trachomatis - O84022
Ashbya gossypii - NP_985249
Saccharomyces cerevisiae - NP_009477
Kluyveromyces lactis - XP_454879
Schizosaccharomyces pombe - NP_595569
Magnaporthe oryzae - XP_003716759
Neurospora crassa - XP_956014
Arabidopsis thaliana - NP_192770
Monosiga brevicollis - XP_001742550
Salpingoeca rosetta - XP_004993500
Capsaspora owczarzaki - XP_004345266
Caenorhabditis elegans - NP_501914
Drosophila melanogaster - NP_788565
Anopheles gambiae - XP_320939
Amphimedon queenslandica - XP_003385671
Nematostella vectensis - XP_001630713
Trichoplax adhaerens - XP_002112667
Danio rerio - NP_956190
Xenopus tropicalis - NP_001090690
Gallus gallus - XP_414300
Mus musculus - NP_742012
Rattus norvegicus - NP_001094042
Bos taurus - NP_001094539
Canis lupus - XP_541332
Macaca mulatta - XP_001107299
Pan troglodytes - XP_003312212
Homo sapiens - NP_038203
Archaeoglobus fulgidus - O29622
Methanosarcina acetivorans - Q8TN62
Halobacterium salinarum - Q9HN97
Methanospirillum hungatei - Q2FPJ4
Methanopyrus kandleri - Q8TWY2
Methanothermobacter thermautotrophicus - O27428
Methanocaldococcus jannaschii - Q58357
Pyrococcus furiosus - P46214
Thermococcus kodakaraensis - Q5JJ31
0.3

J. LysRS

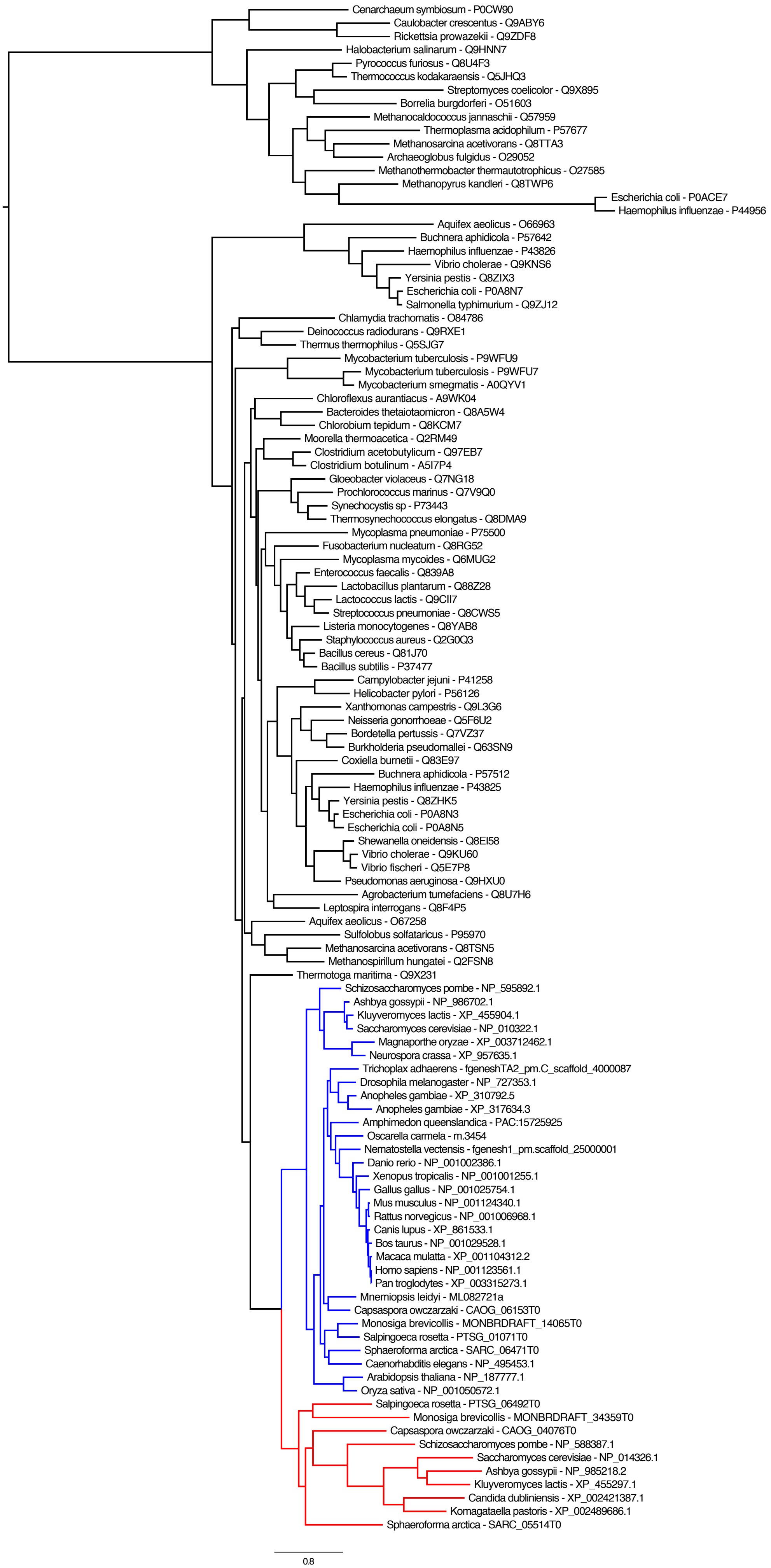

Cenarchaeum symbiosum - P0CW90
Caulobacter crescentus - Q9ABY6
Rickettsia prowazekii - Q9ZDF8
Halobacterium salinarum - Q9HNN7
Pyrococcus furiosus - Q8U4F3
Thermococcus kodakaraensis - Q5JHQ3
Streptomyces coelicolor - Q9X895
Borrelia burgdorferi - O51603
Methanocaldococcus jannaschii - Q57959
Thermoplasma acidophilum - P57677
Methanosarcina acetivorans - Q8TTA3
Archaeoglobus fulgidus - O29052
Methanothermobacter thermautotrophicus - O27585
Methanopyrus kandleri - Q8TWP6
Escherichia coli - P0ACE7
Haemophilus influenzae - P44956
Aquifex aeolicus - O66963
Buchnera aphidicola - P57642
Haemophilus influenzae - P43826
Vibrio cholerae - Q9KNS6
Yersinia pestis - Q8ZIX3
Escherichia coli - P0A8N7
Salmonella typhimurium - Q9ZJ12
Chlamydia trachomatis - O84786
Deinococcus radiodurans - Q9RXE1
Thermus thermophilus - Q5SJG7
Mycobacterium tuberculosis - P9WFU9
Mycobacterium tuberculosis - P9WFU7
Mycobacterium smegmatis - A0QYV1
Chloroflexus aurantiacus - A9WK04
Bacteroides thetaiotaomicron - Q8A5W4
Chlorobium tepidum - Q8KCM7
Moorella thermoacetica - Q2RM49
Clostridium acetobutylicum - Q97EB7
Clostridium botulinum - A5I7P4
Gloeobacter violaceus - Q7NG18
Prochlorococcus marinus - Q7V9Q0
Synechocystis sp - P73443
Thermosynechococcus elongatus - Q8DMA9
Mycoplasma pneumoniae - P75500
Fusobacterium nucleatum - Q8RG52
Mycoplasma mycoides - Q6MUG2
Enterococcus faecalis - Q839A8
Lactobacillus plantarum - Q88Z28
Lactococcus lactis - Q9CII7
Streptococcus pneumoniae - Q8CWS5
Listeria monocytogenes - Q8YAB8
Staphylococcus aureus - Q2G0Q3
Bacillus cereus - Q81J70
Bacillus subtilis - P37477
Campylobacter jejuni - P41258
Helicobacter pylori - P56126
Xanthomonas campestris - Q9L3G6
Neisseria gonorrhoeae - Q5F6U2
Bordetella pertussis - Q7VZ37
Burkholderia pseudomallei - Q63SN9
Coxiella burnetii - Q83E97
Buchnera aphidicola - P57512
Haemophilus influenzae - P43825
Yersinia pestis - Q8ZHK5
Escherichia coli - P0A8N3
Escherichia coli - P0A8N5
Shewanella oneidensis - Q8EI58
Vibrio cholerae - Q9KU60
Vibrio fischeri - Q5E7P8
Pseudomonas aeruginosa - Q9HXU0
Agrobacterium tumefaciens - Q8U7H6
Leptospira interrogans - Q8F4P5
Aquifex aeolicus - O67258
Sulfolobus solfataricus - P95970
Methanosarcina acetivorans - Q8TSN5
Methanospirillum hungatei - Q2FSN8
Thermotoga maritima - Q9X231
Schizosaccharomyces pombe - NP_595892.1
Ashbya gossypii - NP_986702.1
Kluyveromyces lactis - XP_455904.1
Saccharomyces cerevisiae - NP_010322.1
Magnaporthe oryzae - XP_003712462.1
Neurospora crassa - XP_957635.1
Trichoplax adhaerens - fgeneshTA2_pm.C_scaffold_4000087
Drosophila melanogaster - NP_727353.1
Anopheles gambiae - XP_310792.5
Anopheles gambiae - XP_317634.3
Amphimedon queenslandica - PAC:15725925
Oscarella carmela - m.3454
Nematostella vectensis - fgenesh1_pm.scaffold_25000001
Danio rerio - NP_001002386.1
Xenopus tropicalis - NP_001001255.1
Gallus gallus - NP_001025754.1
Mus musculus - NP_001124340.1
Rattus norvegicus - NP_001006968.1
Canis lupus - XP_861533.1
Bos taurus - NP_001029528.1
Macaca mulatta - XP_001104312.2
Homo sapiens - NP_001123561.1
Pan troglodytes - XP_003315273.1
Mnemiopsis leidyi - ML082721a
Capsaspora owczarzaki - CAOG_06153T0
Monosiga brevicollis - MONBRDRAFT_14065T0
Salpingoeca rosetta - PTSG_01071T0
Sphaeroforma arctica - SARC_06471T0
Caenorhabditis elegans - NP_495453.1
Arabidopsis thaliana - NP_187777.1
Oryza sativa - NP_001050572.1
Salpingoeca rosetta - PTSG_06492T0
Monosiga brevicollis - MONBRDRAFT_34359T0
Capsaspora owczarzaki - CAOG_04076T0
Schizosaccharomyces pombe - NP_588387.1
Saccharomyces cerevisiae - NP_014326.1
Ashbya gossypii - NP_985218.2
Kluyveromyces lactis - XP_455297.1
Candida dubliniensis - XP_002421387.1
Komagataella pastoris - XP_002489686.1
Sphaeroforma arctica - SARC_05514T0
0.8

K. LeuRS

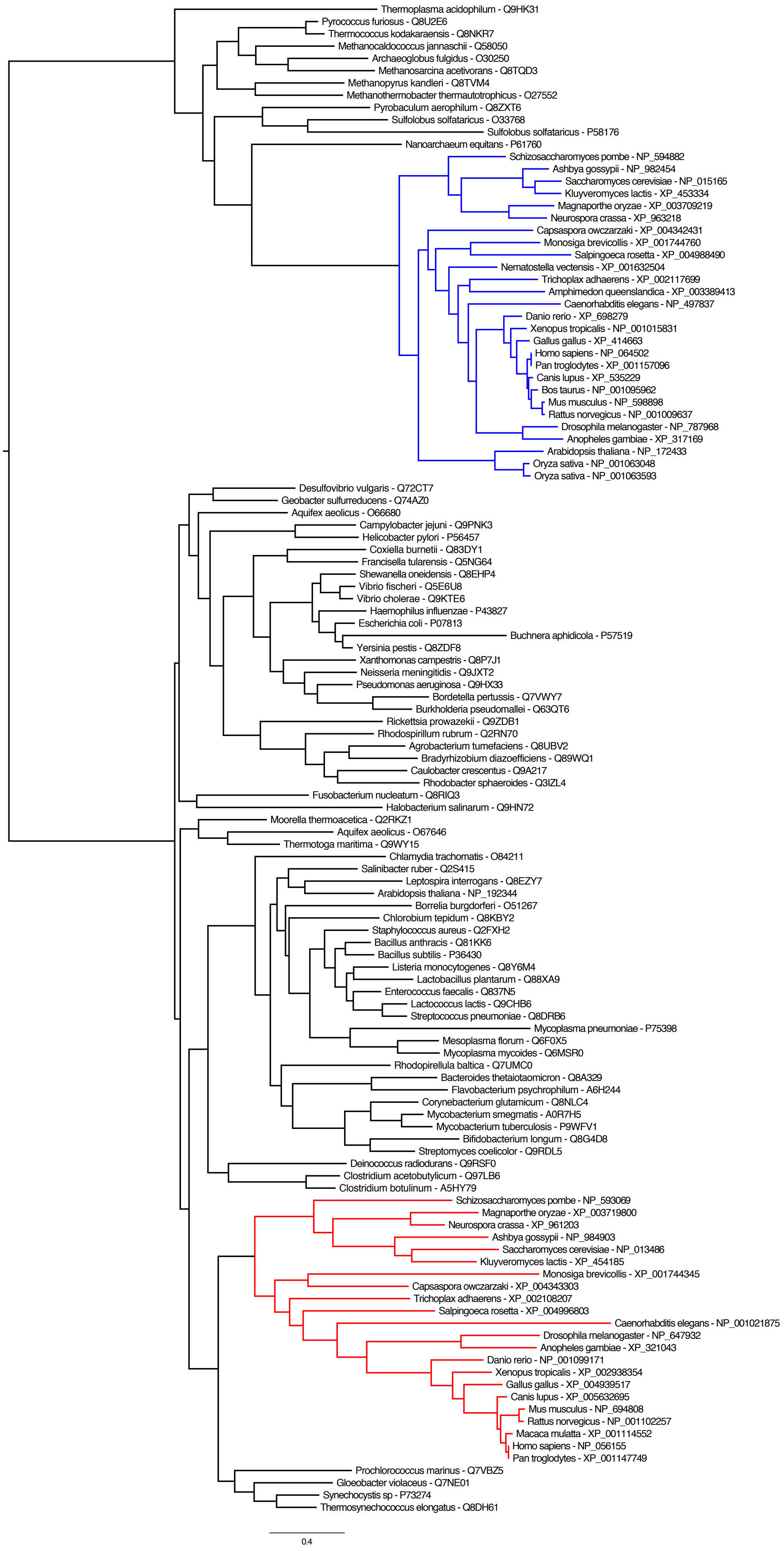
Thermoplasma acidophilum - Q9HK31
Pyrococcus furiosus - Q8U2E6
Thermococcus kodakaraensis - Q8NKR7
Methanocaldococcus jannaschii - Q58050
Archaeoglobus fulgidus - O30250
Methanosarcina acetivorans - Q8TQD3
Methanopyrus kandleri - Q8TVM4
Methanothermobacter thermautotrophicus - O27552
Pyrobaculum aerophilum - Q8ZXT6
Sulfolobus solfataricus - O33768
Sulfolobus solfataricus - P58176
Nanoarchaeum equitans - P61760
Schizosaccharomyces pombe - NP_594882
Ashbya gossypii - NP_982454
Saccharomyces cerevisiae - NP_015165
Kluyveromyces lactis - XP_453334
Magnaporthe oryzae - XP_003709219
Neurospora crassa - XP_963218
Capsaspora owczarzaki - XP_004342431
Monosiga brevicollis - XP_001744760
Salpingoeca rosetta - XP_004988490
Nematostella vectensis - XP_001632504
Trichoplax adhaerens - XP_002117699
Amphimedon queenslandica - XP_003389413
Caenorhabditis elegans - NP_497837
Danio rerio - XP_698279
Xenopus tropicalis - NP_001015831
Gallus gallus - XP_414663
Homo sapiens - NP_064502
Pan troglodytes - XP_001157096
Canis lupus - XP_535229
Bos taurus - NP_001095962
Mus musculus - NP_598898
Rattus norvegicus - NP_001009637
Drosophila melanogaster - NP_787968
Anopheles gambiae - XP_317169
Arabidopsis thaliana - NP_172433
Oryza sativa - NP_001063048
Oryza sativa - NP_001063593
Desulfovibrio vulgaris - Q72CT7
Geobacter sulfurreducens - Q74AZ0
Aquifex aeolicus - O66680
Campylobacter jejuni - Q9PNK3
Helicobacter pylori - P56457
Coxiella burnetii - Q83DY1
Francisella tularensis - Q5NG64
Shewanella oneidensis - Q8EHP4
Vibrio fischeri - Q5E6U8
Vibrio cholerae - Q9KTE6
Haemophilus influenzae - P43827
Escherichia coli - P07813
Buchnera aphidicola - P57519
Yersinia pestis - Q8ZDF8
Xanthomonas campestris - Q8P7J1
Neisseria meningitidis - Q9JXT2
Pseudomonas aeruginosa - Q9HX33
Bordetella pertussis - Q7VWY7
Burkholderia pseudomallei - Q63QT6
Rickettsia prowazekii - Q9ZDB1
Rhodospirillum rubrum - Q2RN70
Agrobacterium tumefaciens - Q8UBV2
Bradyrhizobium diazoefficiens - Q89WQ1
Caulobacter crescentus - Q9A217
Rhodobacter sphaeroides - Q3IZL4
Fusobacterium nucleatum - Q8RIQ3
Halobacterium salinarum - Q9HN72
Moorella thermoacetica - Q2RKZ1
Aquifex aeolicus - O67646
Thermotoga maritima - Q9WY15
Chlamydia trachomatis - O84211
Salinibacter ruber - Q2S415
Leptospira interrogans - Q8EZY7
Arabidopsis thaliana - NP_192344
Borrelia burgdorferi - O51267
Chlorobium tepidum - Q8KBY2
Staphylococcus aureus - Q2FXH2
Bacillus anthracis - Q81KK6
Bacillus subtilis - P36430
Listeria monocytogenes - Q8Y6M4
Lactobacillus plantarum - Q88XA9
Enterococcus faecalis - Q837N5
Lactococcus lactis - Q9CHB6
Streptococcus pneumoniae - Q8DRB6
Mycoplasma pneumoniae - P75398
Mesoplasma florum - Q6F0X5
Mycoplasma mycoides - Q6MSR0
Rhodopirellula baltica - Q7UMC0
Bacteroides thetaiotaomicron - Q8A329
Flavobacterium psychrophilum - A6H244
Corynebacterium glutamicum - Q8NLC4
Mycobacterium smegmatis - A0R7H5
Mycobacterium tuberculosis - P9WFV1
Bifidobacterium longum - Q8G4D8
Streptomyces coelicolor - Q9RDL5
Deinococcus radiodurans - Q9RSF0
Clostridium acetobutylicum - Q97LB6
Clostridium botulinum - A5HY79
Schizosaccharomyces pombe - NP_593069
Magnaporthe oryzae - XP_003719800
Neurospora crassa - XP_961203
Ashbya gossypii - NP_984903
Saccharomyces cerevisiae - NP_013486
Kluyveromyces lactis - XP_454185
Monosiga brevicollis - XP_001744345
Capsaspora owczarzaki - XP_004343303
Trichoplax adhaerens - XP_002108207
Salpingoeca rosetta - XP_004996803
Caenorhabditis elegans - NP_001021875
Drosophila melanogaster - NP_647932
Anopheles gambiae - XP_321043
Danio rerio - NP_001099171
Xenopus tropicalis - XP_002938354
Gallus gallus - XP_004939517
Canis lupus - XP_005632695
Mus musculus - NP_694808
Rattus norvegicus - NP_001102257
Macaca mulatta - XP_001114552
Homo sapiens - NP_056155
Pan troglodytes - XP_001147749
Prochlorococcus marinus - Q7VBZ5
Gloeobacter violaceus - Q7NE01
Synechocystis sp - P73274
Thermosynechococcus elongatus - Q8DH61
0.4

L. MetRS

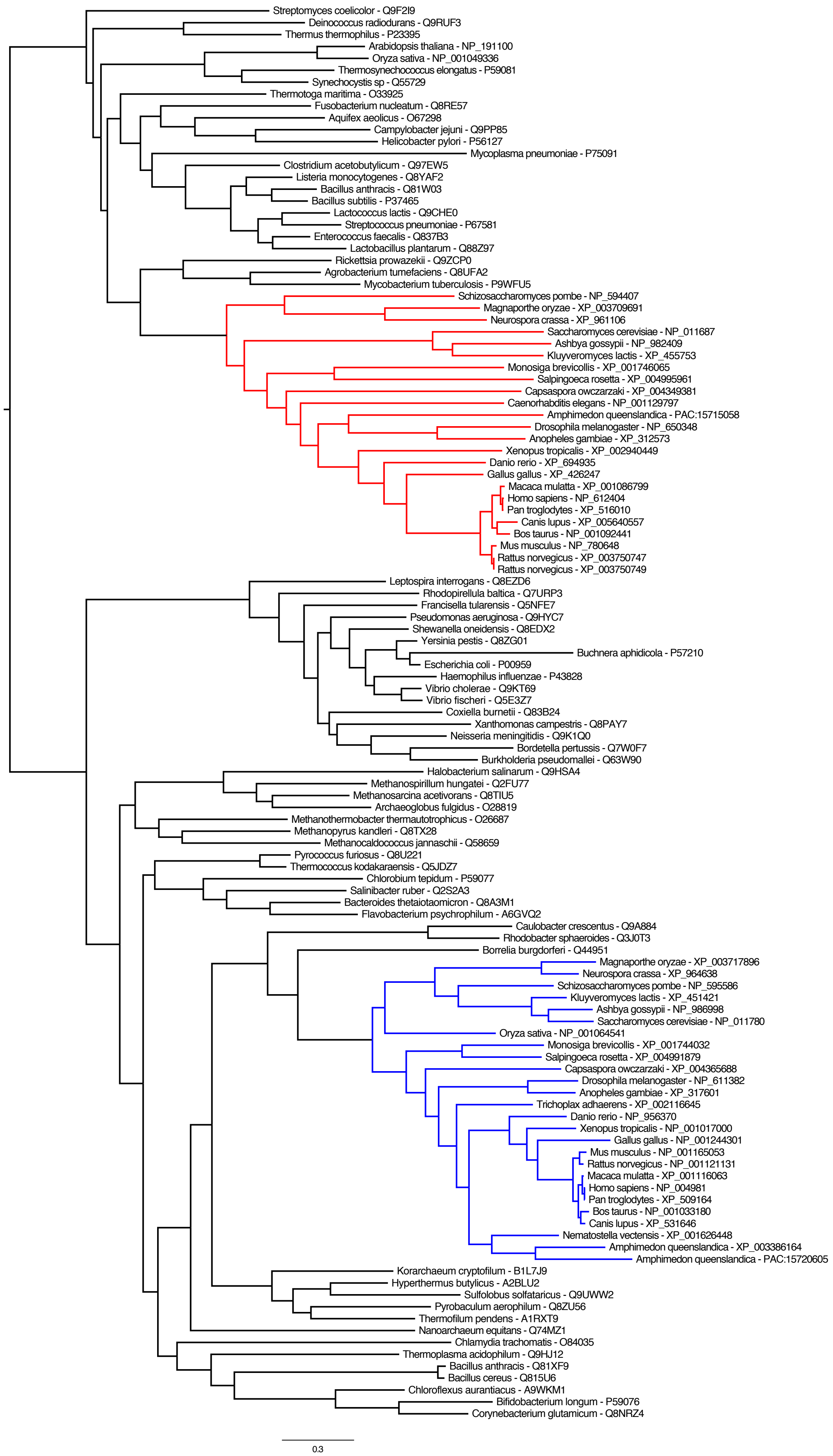

Streptomyces coelicolor - Q9F2I9
Deinococcus radiodurans - Q9RUF3
Thermus thermophilus - P23395
Arabidopsis thaliana - NP_191100
Oryza sativa - NP_001049336
Thermosynechococcus elongatus - P59081
Synechocystis sp - Q55729
Thermotoga maritima - O33925
Fusobacterium nucleatum - Q8RE57
Aquifex aeolicus - O67298
Campylobacter jejuni - Q9PP85
Helicobacter pylori - P56127
Mycoplasma pneumoniae - P75091
Clostridium acetobutylicum - Q97EW5
Listeria monocytogenes - Q8YAF2
Bacillus anthracis - Q81W03
Bacillus subtilis - P37465
Lactococcus lactis - Q9CHE0
Streptococcus pneumoniae - P67581
Enterococcus faecalis - Q837B3
Lactobacillus plantarum - Q88Z97
Rickettsia prowazekii - Q9ZCP0
Agrobacterium tumefaciens - Q8UFA2
Mycobacterium tuberculosis - P9WFU5
Schizosaccharomyces pombe - NP_594407
Magnaporthe oryzae - XP_003709691
Neurospora crassa - XP_961106
Saccharomyces cerevisiae - NP_011687
Ashbya gossypii - NP_982409
Kluyveromyces lactis - XP_455753
Monosiga brevicollis - XP_001746065
Salpingoeca rosetta - XP_004995961
Capsaspora owczarzaki - XP_004349381
Caenorhabditis elegans - NP_001129797
Amphimedon queenslandica - PAC:15715058
Drosophila melanogaster - NP_650348
Anopheles gambiae - XP_312573
Xenopus tropicalis - XP_002940449
Danio rerio - XP_694935
Gallus gallus - XP_426247
Macaca mulatta - XP_001086799
Homo sapiens - NP_612404
Pan troglodytes - XP_516010
Canis lupus - XP_005640557
Bos taurus - NP_001092441
Mus musculus - NP_780648
Rattus norvegicus - XP_003750747
Rattus norvegicus - XP_003750749
Leptospira interrogans - Q8EZD6
Rhodopirellula baltica - Q7URP3
Francisella tularensis - Q5NFE7
Pseudomonas aeruginosa - Q9HYC7
Shewanella oneidensis - Q8EDX2
Yersinia pestis - Q8ZG01
Buchnera aphidicola - P57210
Escherichia coli - P00959
Haemophilus influenzae - P43828
Vibrio cholerae - Q9KT69
Vibrio fischeri - Q5E3Z7
Coxiella burnetii - Q83B24
Xanthomonas campestris - Q8PAY7
Neisseria meningitidis - Q9K1Q0
Bordetella pertussis - Q7W0F7
Burkholderia pseudomallei - Q63W90
Halobacterium salinarum - Q9HSA4
Methanospirillum hungatei - Q2FU77
Methanosarcina acetivorans - Q8TIU5
Archaeoglobus fulgidus - O28819
Methanothermobacter thermautotrophicus - O26687
Methanopyrus kandleri - Q8TX28
Methanocaldococcus jannaschii - Q58659
Pyrococcus furiosus - Q8U221
Thermococcus kodakaraensis - Q5JDZ7
Chlorobium tepidum - P59077
Salinibacter ruber - Q2S2A3
Bacteroides thetaiotaomicron - Q8A3M1
Flavobacterium psychrophilum - A6GVQ2
Caulobacter crescentus - Q9A884
Rhodobacter sphaeroides - Q3J0T3
Borrelia burgdorferi - Q44951
Magnaporthe oryzae - XP_003717896
Neurospora crassa - XP_964638
Schizosaccharomyces pombe - NP_595586
Kluyveromyces lactis - XP_451421
Ashbya gossypii - NP_986998
Saccharomyces cerevisiae - NP_011780
Oryza sativa - NP_001064541
Monosiga brevicollis - XP_001744032
Salpingoeca rosetta - XP_004991879
Capsaspora owczarzaki - XP_004365688
Drosophila melanogaster - NP_611382
Anopheles gambiae - XP_317601
Trichoplax adhaerens - XP_002116645
Danio rerio - NP_956370
Xenopus tropicalis - NP_001017000
Gallus gallus - NP_001244301
Mus musculus - NP_001165053
Rattus norvegicus - NP_001121131
Macaca mulatta - XP_001116063
Homo sapiens - NP_004981
Pan troglodytes - XP_509164
Bos taurus - NP_001033180
Canis lupus - XP_531646
Nematostella vectensis - XP_001626448
Amphimedon queenslandica - XP_003386164
Amphimedon queenslandica - PAC:15720605
Korarchaeum cryptofilum - B1L7J9
Hyperthermus butylicus - A2BLU2
Sulfolobus solfataricus - Q9UWW2
Pyrobaculum aerophilum - Q8ZU56
Thermofilum pendens - A1RXT9
Nanoarchaeum equitans - Q74MZ1
Chlamydia trachomatis - O84035
Thermoplasma acidophilum - Q9HJ12
Bacillus anthracis - Q81XF9
Bacillus cereus - Q815U6
Chloroflexus aurantiacus - A9WKM1
Bifidobacterium longum - P59076
Corynebacterium glutamicum - Q8NRZ4
0.3

M. AsnRS

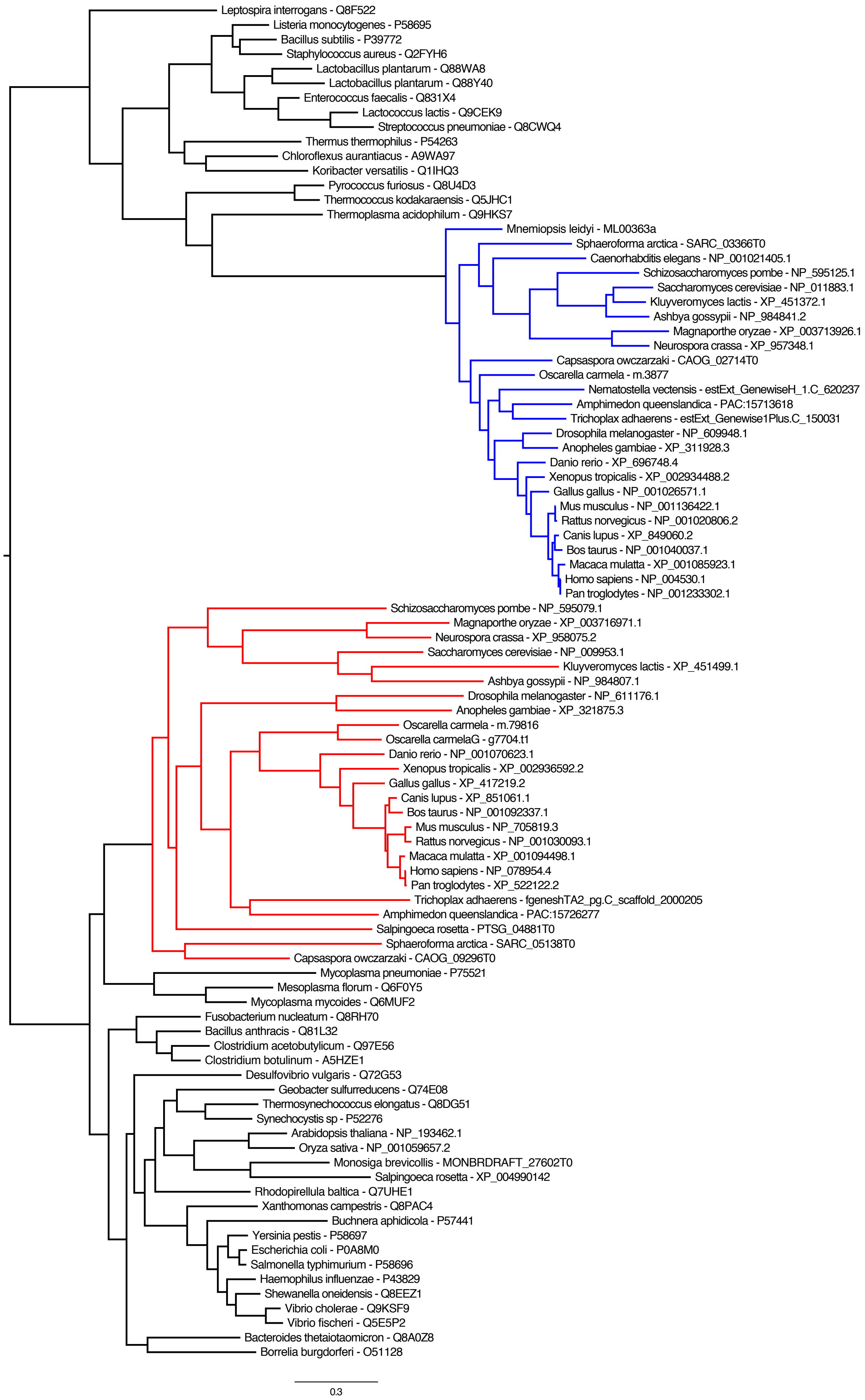

Leptospira interrogans - Q8F522
Listeria monocytogenes - P58695
Bacillus subtilis - P39772
Staphylococcus aureus - Q2FYH6
Lactobacillus plantarum - Q88WA8
Lactobacillus plantarum - Q88Y40
Enterococcus faecalis - Q831X4
Lactococcus lactis - Q9CEK9
Streptococcus pneumoniae - Q8CWQ4
Thermus thermophilus - P54263
Chloroflexus aurantiacus - A9WA97
Koribacter versatilis - Q1IHQ3
Pyrococcus furiosus - Q8U4D3
Thermococcus kodakaraensis - Q5JHC1
Thermoplasma acidophilum - Q9HKS7
Mnemiopsis leidyi - ML00363a
Sphaeroforma arctica - SARC_03366T0
Caenorhabditis elegans - NP_001021405.1
Schizosaccharomyces pombe - NP_595125.1
Saccharomyces cerevisiae - NP_011883.1
Kluyveromyces lactis - XP_451372.1
Ashbya gossypii - NP_984841.2
Magnaporthe oryzae - XP_003713926.1
Neurospora crassa - XP_957348.1
Capsaspora owczarzaki - CAOG_02714T0
Oscarella carmela - m.3877
Nematostella vectensis - estExt_GenewiseH_1.C_620237
Amphimedon queenslandica - PAC:15713618
Trichoplax adhaerens - estExt_Genewise1Plus.C_150031
Drosophila melanogaster - NP_609948.1
Anopheles gambiae - XP_311928.3
Danio rerio - XP_696748.4
Xenopus tropicalis - XP_002934488.2
Gallus gallus - NP_001026571.1
Mus musculus - NP_001136422.1
Rattus norvegicus - NP_001020806.2
Canis lupus - XP_849060.2
Bos taurus - NP_001040037.1
Macaca mulatta - XP_001085923.1
Homo sapiens - NP_004530.1
Pan troglodytes - NP_001233302.1
Schizosaccharomyces pombe - NP_595079.1
Magnaporthe oryzae - XP_003716971.1
Neurospora crassa - XP_958075.2
Saccharomyces cerevisiae - NP_009953.1
Kluyveromyces lactis - XP_451499.1
Ashbya gossypii - NP_984807.1
Drosophila melanogaster - NP_611176.1
Anopheles gambiae - XP_321875.3
Oscarella carmela - m.79816
Oscarella carmelaG - g7704.t1
Danio rerio - NP_001070623.1
Xenopus tropicalis - XP_002936592.2
Gallus gallus - XP_417219.2
Canis lupus - XP_851061.1
Bos taurus - NP_001092337.1
Mus musculus - NP_705819.3
Rattus norvegicus - NP_001030093.1
Macaca mulatta - XP_001094498.1
Homo sapiens - NP_078954.4
Pan troglodytes - XP_522122.2
Trichoplax adhaerens - fgeneshTA2_pg.C_scaffold_2000205
Amphimedon queenslandica - PAC:15726277
Salpingoeca rosetta - PTSG_04881T0
Sphaeroforma arctica - SARC_05138T0
Capsaspora owczarzaki - CAOG_09296T0
Mycoplasma pneumoniae - P75521
Mesoplasma florum - Q6F0Y5
Mycoplasma mycoides - Q6MUF2
Fusobacterium nucleatum - Q8RH70
Bacillus anthracis - Q81L32
Clostridium acetobutylicum - Q97E56
Clostridium botulinum - A5HZE1
Desulfovibrio vulgaris - Q72G53
Geobacter sulfurreducens - Q74E08
Thermosynechococcus elongatus - Q8DG51
Synechocystis sp - P52276
Arabidopsis thaliana - NP_193462.1
Oryza sativa - NP_001059657.2
Monosiga brevicollis - MONBRDRAFT_27602T0
Salpingoeca rosetta - XP_004990142
Rhodopirellula baltica - Q7UHE1
Xanthomonas campestris - Q8PAC4
Buchnera aphidicola - P57441
Yersinia pestis - P58697
Escherichia coli - P0A8M0
Salmonella typhimurium - P58696
Haemophilus influenzae - P43829
Shewanella oneidensis - Q8EEZ1
Vibrio cholerae - Q9KSF9
Vibrio fischeri - Q5E5P2
Bacteroides thetaiotaomicron - Q8A0Z8
Borrelia burgdorferi - O51128
0.3

N. Pro/GluProRS

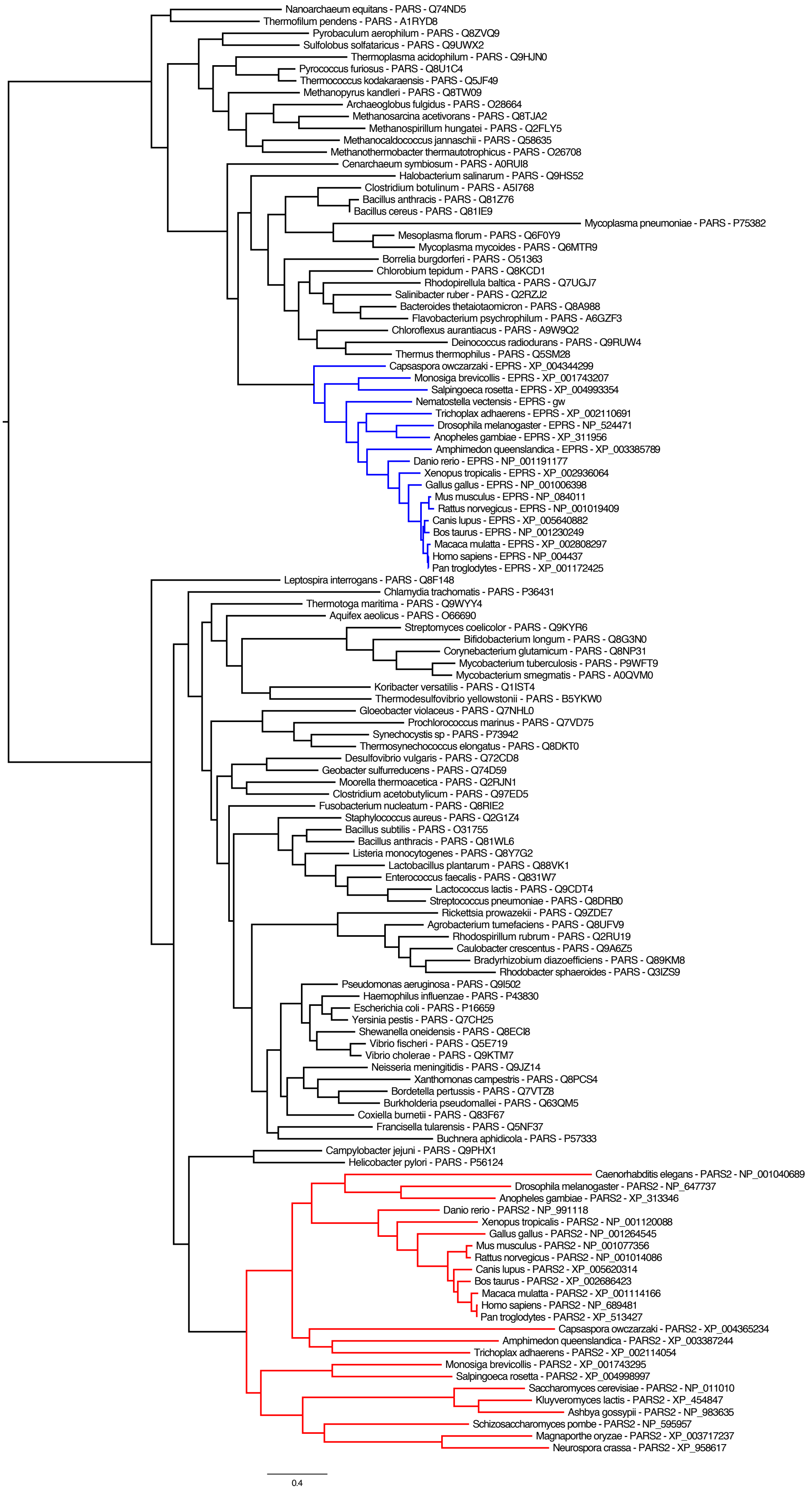

O. ArgRS

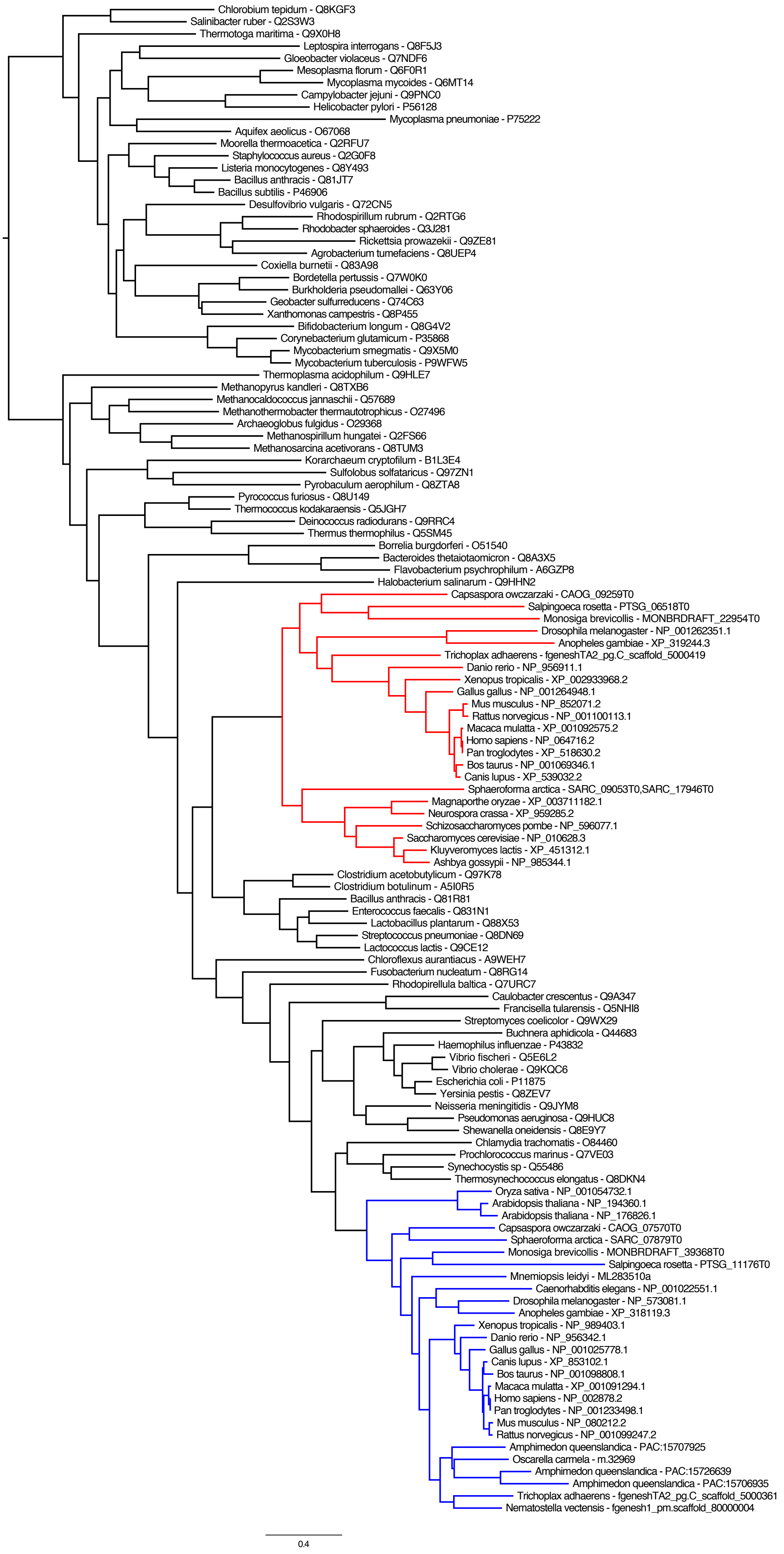
Chlorobium tepidum - Q8KGF3
Salinibacter ruber - Q2S3W3
Thermotoga maritima - Q9X0H8
Leptospira interrogans - Q8F5J3
Gloeobacter violaceus - Q7NDF6
Mesoplasma florum - Q6F0R1
Mycoplasma mycoides - Q6MT14
Campylobacter jejuni - Q9PNC0
Helicobacter pylori - P56128
Mycoplasma pneumoniae - P75222
Aquifex aeolicus - O67068
Moorella thermoacetica - Q2RFU7
Staphylococcus aureus - Q2G0F8
Listeria monocytogenes - Q8Y493
Bacillus anthracis - Q81JT7
Bacillus subtilis - P46906
Desulfovibrio vulgaris - Q72CN5
Rhodospirillum rubrum - Q2RTG6
Rhodobacter sphaeroides - Q3J281
Rickettsia prowazekii - Q9ZE81
Agrobacterium tumefaciens - Q8UEP4
Coxiella burnetii - Q83A98
Bordetella pertussis - Q7W0K0
Burkholderia pseudomallei - Q63Y06
Geobacter sulfurreducens - Q74C63
Xanthomonas campestris - Q8P455
Bifidobacterium longum - Q8G4V2
Corynebacterium glutamicum - P35868
Mycobacterium smegmatis - Q9X5M0
Mycobacterium tuberculosis - P9WFW5
Thermoplasma acidophilum - Q9HLE7
Methanopyrus kandleri - Q8TXB6
Methanocaldococcus jannaschii - Q57689
Methanothermobacter thermautotrophicus - O27496
Archaeoglobus fulgidus - O29368
Methanospirillum hungatei - Q2FS66
Methanosarcina acetivorans - Q8TUM3
Korarchaeum cryptofilum - B1L3E4
Sulfolobus solfataricus - Q97ZN1
Pyrobaculum aerophilum - Q8ZTA8
Pyrococcus furiosus - Q8U149
Thermococcus kodakaraensis - Q5JGH7
Deinococcus radiodurans - Q9RRC4
Thermus thermophilus - Q5SM45
Borrelia burgdorferi - O51540
Bacteroides thetaiotaomicron - Q8A3X5
Flavobacterium psychrophilum - A6GZP8
Halobacterium salinarum - Q9HHN2
Capsaspora owczarzaki - CAOG_09259T0
Salpingoeca rosetta - PTSG_06518T0
Monosiga brevicollis - MONBRDRAFT_22954T0
Drosophila melanogaster - NP_001262351.1
Anopheles gambiae - XP_319244.3
Trichoplax adhaerens - fgeneshTA2_pg.C_scaffold_5000419
Danio rerio - NP_956911.1
Xenopus tropicalis - XP_002933968.2
Gallus gallus - NP_001264948.1
Mus musculus - NP_852071.2
Rattus norvegicus - NP_001100113.1
Macaca mulatta - XP_001092575.2
Homo sapiens - NP_064716.2
Pan troglodytes - XP_518630.2
Bos taurus - NP_001069346.1
Canis lupus - XP_539032.2
Sphaeroforma arctica - SARC_09053T0,SARC_17946T0
Magnaporthe oryzae - XP_003711182.1
Neurospora crassa - XP_959285.2
Schizosaccharomyces pombe - NP_596077.1
Saccharomyces cerevisiae - NP_010628.3
Kluyveromyces lactis - XP_451312.1
Ashbya gossypii - NP_985344.1
Clostridium acetobutylicum - Q97K78
Clostridium botulinum - A5I0R5
Bacillus anthracis - Q81R81
Enterococcus faecalis - Q831N1
Lactobacillus plantarum - Q88X53
Streptococcus pneumoniae - Q8DN69
Lactococcus lactis - Q9CE12
Chloroflexus aurantiacus - A9WEH7
Fusobacterium nucleatum - Q8RG14
Rhodopirellula baltica - Q7URC7
Caulobacter crescentus - Q9A347
Francisella tularensis - Q5NHI8
Streptomyces coelicolor - Q9WX29
Buchnera aphidicola - Q44683
Haemophilus influenzae - P43832
Vibrio fischeri - Q5E6L2
Vibrio cholerae - Q9KQC6
Escherichia coli - P11875
Yersinia pestis - Q8ZEV7
Neisseria meningitidis - Q9JYM8
Pseudomonas aeruginosa - Q9HUC8
Shewanella oneidensis - Q8E9Y7
Chlamydia trachomatis - O84460
Prochlorococcus marinus - Q7VE03
Synechocystis sp - Q55486
Thermosynechococcus elongatus - Q8DKN4
Oryza sativa - NP_001054732.1
Arabidopsis thaliana - NP_194360.1
Arabidopsis thaliana - NP_176826.1
Capsaspora owczarzaki - CAOG_07570T0
Sphaeroforma arctica - SARC_07879T0
Monosiga brevicollis - MONBRDRAFT_39368T0
Salpingoeca rosetta - PTSG_11176T0
Mnemiopsis leidyi - ML283510a
Caenorhabditis elegans - NP_001022551.1
Drosophila melanogaster - NP_573081.1
Anopheles gambiae - XP_318119.3
Xenopus tropicalis - NP_989403.1
Danio rerio - NP_956342.1
Gallus gallus - NP_001025778.1
Canis lupus - XP_853102.1
Bos taurus - NP_001098808.1
Macaca mulatta - XP_001091294.1
Homo sapiens - NP_002878.2
Pan troglodytes - NP_001233498.1
Mus musculus - NP_080212.2
Rattus norvegicus - NP_001099247.2
Amphimedon queenslandica - PAC:15707925
Oscarella carmela - m.32969
Amphimedon queenslandica - PAC:15726639
Amphimedon queenslandica - PAC:15706935
Trichoplax adhaerens - fgeneshTA2_pg.C_scaffold_5000361
Nematostella vectensis - fgenesh1_pm.scaffold_80000004
0.4

P. SerRS

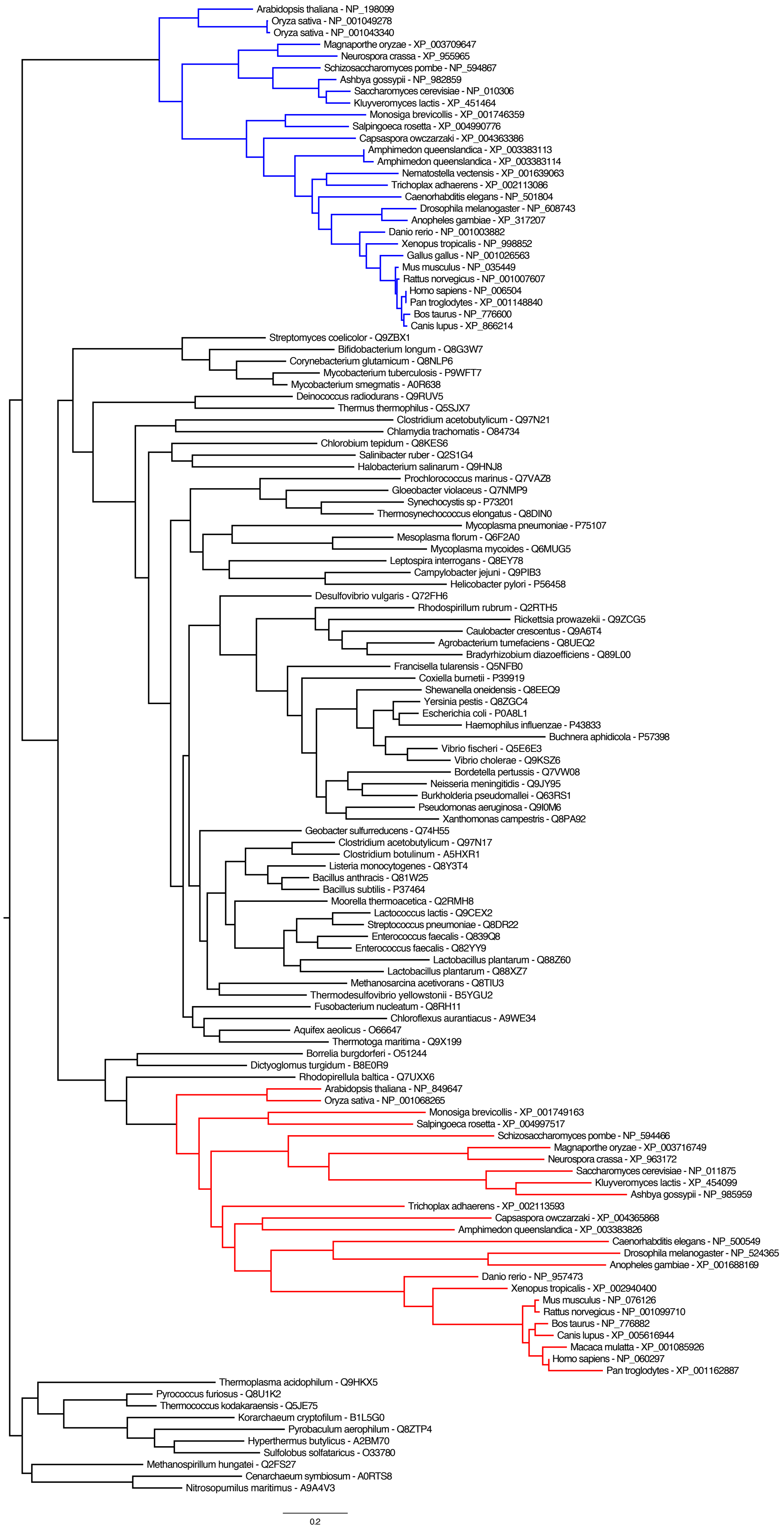

Arabidopsis thaliana - NP_198099
Oryza sativa - NP_001049278
Oryza sativa - NP_001043340
Magnaporthe oryzae - XP_003709647
Neurospora crassa - XP_955965
Schizosaccharomyces pombe - NP_594867
Ashbya gossypii - NP_982859
Saccharomyces cerevisiae - NP_010306
Kluyveromyces lactis - XP_451464
Monosiga brevicollis - XP_001746359
Salpingoeca rosetta - XP_004990776
Capsaspora owczarzaki - XP_004363386
Amphimedon queenslandica - XP_003383113
Amphimedon queenslandica - XP_003383114
Nematostella vectensis - XP_001639063
Trichoplax adhaerens - XP_002113086
Caenorhabditis elegans - NP_501804
Drosophila melanogaster - NP_608743
Anopheles gambiae - XP_317207
Danio rerio - NP_001003882
Xenopus tropicalis - NP_998852
Gallus gallus - NP_001026563
Mus musculus - NP_035449
Rattus norvegicus - NP_001007607
Homo sapiens - NP_006504
Pan troglodytes - XP_001148840
Bos taurus - NP_776600
Canis lupus - XP_866214
Streptomyces coelicolor - Q9ZBX1
Bifidobacterium longum - Q8G3W7
Corynebacterium glutamicum - Q8NLP6
Mycobacterium tuberculosis - P9WFT7
Mycobacterium smegmatis - A0R638
Deinococcus radiodurans - Q9RUV5
Thermus thermophilus - Q5SJX7
Clostridium acetobutylicum - Q97N21
Chlamydia trachomatis - O84734
Chlorobium tepidum - Q8KES6
Salinibacter ruber - Q2S1G4
Halobacterium salinarum - Q9HNJ8
Prochlorococcus marinus - Q7VAZ8
Gloeobacter violaceus - Q7NMP9
Synechocystis sp - P73201
Thermosynechococcus elongatus - Q8DIN0
Mycoplasma pneumoniae - P75107
Mesoplasma florum - Q6F2A0
Mycoplasma mycoides - Q6MUG5
Leptospira interrogans - Q8EY78
Campylobacter jejuni - Q9PIB3
Helicobacter pylori - P56458
Desulfovibrio vulgaris - Q72FH6
Rhodospirillum rubrum - Q2RTH5
Rickettsia prowazekii - Q9ZCG5
Caulobacter crescentus - Q9A6T4
Agrobacterium tumefaciens - Q8UEQ2
Bradyrhizobium diazoefficiens - Q89L00
Francisella tularensis - Q5NFB0
Coxiella burnetii - P39919
Shewanella oneidensis - Q8EEQ9
Yersinia pestis - Q8ZGC4
Escherichia coli - P0A8L1
Haemophilus influenzae - P43833
Buchnera aphidicola - P57398
Vibrio fischeri - Q5E6E3
Vibrio cholerae - Q9KSZ6
Bordetella pertussis - Q7VW08
Neisseria meningitidis - Q9JY95
Burkholderia pseudomallei - Q63RS1
Pseudomonas aeruginosa - Q9I0M6
Xanthomonas campestris - Q8PA92
Geobacter sulfurreducens - Q74H55
Clostridium acetobutylicum - Q97N17
Clostridium botulinum - A5HXR1
Listeria monocytogenes - Q8Y3T4
Bacillus anthracis - Q81W25
Bacillus subtilis - P37464
Moorella thermoacetica - Q2RMH8
Lactococcus lactis - Q9CEX2
Streptococcus pneumoniae - Q8DR22
Enterococcus faecalis - Q839Q8
Enterococcus faecalis - Q82YY9
Lactobacillus plantarum - Q88Z60
Lactobacillus plantarum - Q88XZ7
Methanosarcina acetivorans - Q8TIU3
Thermodesulfovibrio yellowstonii - B5YGU2
Fusobacterium nucleatum - Q8RH11
Chloroflexus aurantiacus - A9WE34
Aquifex aeolicus - O66647
Thermotoga maritima - Q9X199
Borrelia burgdorferi - O51244
Dictyoglomus turgidum - B8E0R9
Rhodopirellula baltica - Q7UXX6
Arabidopsis thaliana - NP_849647
Oryza sativa - NP_001068265
Monosiga brevicollis - XP_001749163
Salpingoeca rosetta - XP_004997517
Schizosaccharomyces pombe - NP_594466
Magnaporthe oryzae - XP_003716749
Neurospora crassa - XP_963172
Saccharomyces cerevisiae - NP_011875
Kluyveromyces lactis - XP_454099
Ashbya gossypii - NP_985959
Trichoplax adhaerens - XP_002113593
Capsaspora owczarzaki - XP_004365868
Amphimedon queenslandica - XP_003383826
Caenorhabditis elegans - NP_500549
Drosophila melanogaster - NP_524365
Anopheles gambiae - XP_001688169
Danio rerio - NP_957473
Xenopus tropicalis - XP_002940400
Mus musculus - NP_076126
Rattus norvegicus - NP_001099710
Bos taurus - NP_776882
Canis lupus - XP_005616944
Macaca mulatta - XP_001085926
Homo sapiens - NP_060297
Pan troglodytes - XP_001162887
Thermoplasma acidophilum - Q9HKX5
Pyrococcus furiosus - Q8U1K2
Thermococcus kodakaraensis - Q5JE75
Korarchaeum cryptofilum - B1L5G0
Pyrobaculum aerophilum - Q8ZTP4
Hyperthermus butylicus - A2BM70
Sulfolobus solfataricus - O33780
Methanospirillum hungatei - Q2FS27
Cenarchaeum symbiosum - A0RTS8
Nitrosopumilus maritimus - A9A4V3
0.2

Q. ThrRS

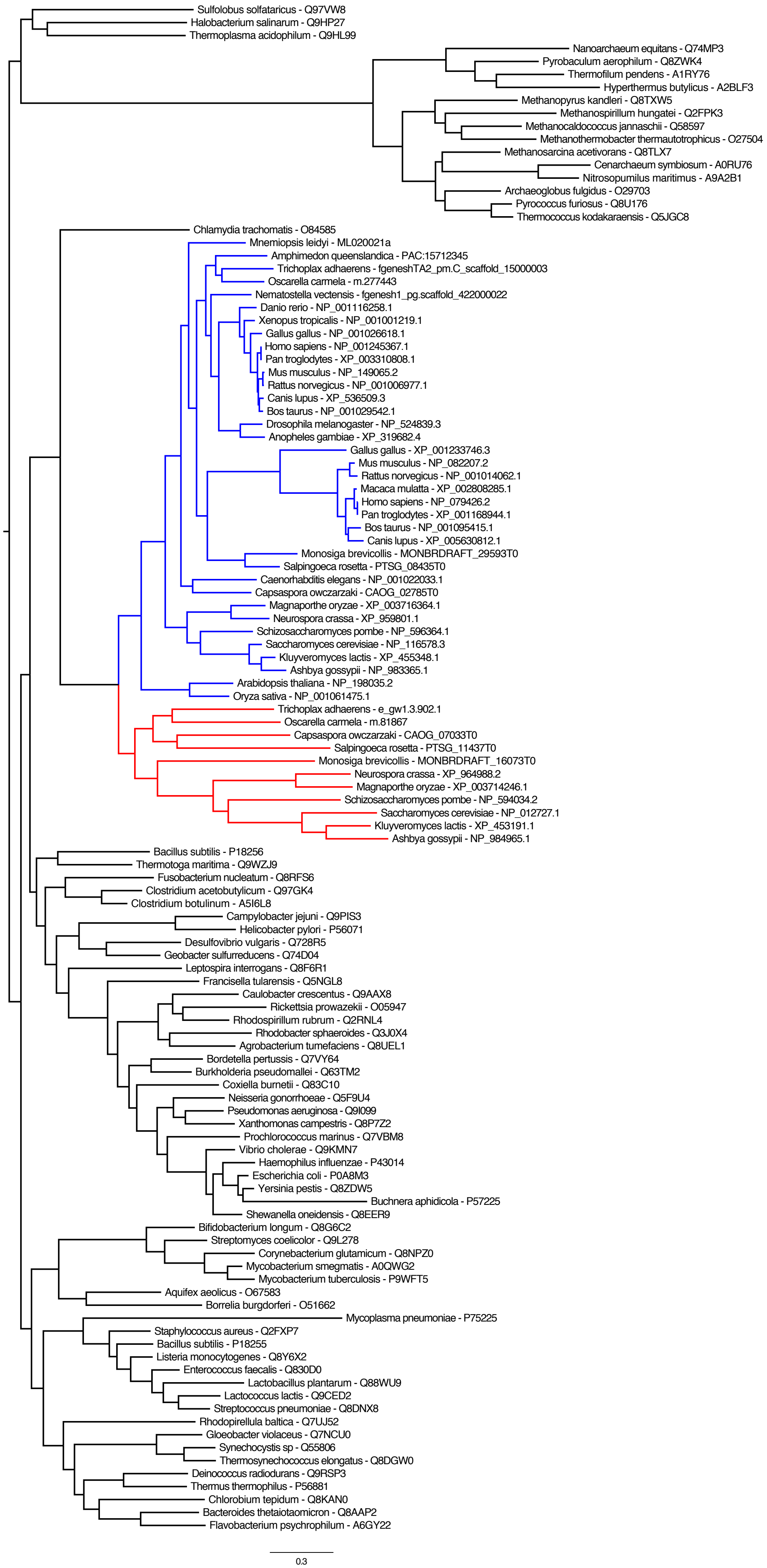
Sulfolobus solfataricus - Q97VW8
Halobacterium salinarum - Q9HP27
Thermoplasma acidophilum - Q9HL99
Nanoarchaeum equitans - Q74MP3
Pyrobaculum aerophilum - Q8ZWK4
Thermofilum pendens - A1RY76
Hyperthermus butylicus - A2BLF3
Methanopyrus kandleri - Q8TXW5
Methanospirillum hungatei - Q2FPK3
Methanocaldococcus jannaschii - Q58597
Methanothermobacter thermautotrophicus - O27504
Methanosarcina acetivorans - Q8TLX7
Cenarchaeum symbiosum - A0RU76
Nitrosopumilus maritimus - A9A2B1
Archaeoglobus fulgidus - O29703
Pyrococcus furiosus - Q8U176
Thermococcus kodakaraensis - Q5JGC8
Chlamydia trachomatis - O84585
Mnemiopsis leidyi - ML020021a
Amphimedon queenslandica - PAC:15712345
Trichoplax adhaerens - fgeneshTA2_pm.C_scaffold_15000003
Oscarella carmela - m.277443
Nematostella vectensis - fgenesh1_pg.scaffold_422000022
Danio rerio - NP_001116258.1
Xenopus tropicalis - NP_001001219.1
Gallus gallus - NP_001026618.1
Homo sapiens - NP_001245367.1
Pan troglodytes - XP_003310808.1
Mus musculus - NP_149065.2
Rattus norvegicus - NP_001006977.1
Canis lupus - XP_536509.3
Bos taurus - NP_001029542.1
Drosophila melanogaster - NP_524839.3
Anopheles gambiae - XP_319682.4
Gallus gallus - XP_001233746.3
Mus musculus - NP_082207.2
Rattus norvegicus - NP_001014062.1
Macaca mulatta - XP_002808285.1
Homo sapiens - NP_079426.2
Pan troglodytes - XP_001168944.1
Bos taurus - NP_001095415.1
Canis lupus - XP_005630812.1
Monosiga brevicollis - MONBRDRAFT_29593T0
Salpingoeca rosetta - PTSG_08435T0
Caenorhabditis elegans - NP_001022033.1
Capsaspora owczarzaki - CAOG_02785T0
Magnaporthe oryzae - XP_003716364.1
Neurospora crassa - XP_959801.1
Schizosaccharomyces pombe - NP_596364.1
Saccharomyces cerevisiae - NP_116578.3
Kluyveromyces lactis - XP_455348.1
Ashbya gossypii - NP_983365.1
Arabidopsis thaliana - NP_198035.2
Oryza sativa - NP_001061475.1
Trichoplax adhaerens - e_gw1.3.902.1
Oscarella carmela - m.81867
Capsaspora owczarzaki - CAOG_07033T0
Salpingoeca rosetta - PTSG_11437T0
Monosiga brevicollis - MONBRDRAFT_16073T0
Neurospora crassa - XP_964988.2
Magnaporthe oryzae - XP_003714246.1
Schizosaccharomyces pombe - NP_594034.2
Saccharomyces cerevisiae - NP_012727.1
Kluyveromyces lactis - XP_453191.1
Ashbya gossypii - NP_984965.1
Bacillus subtilis - P18256
Thermotoga maritima - Q9WZJ9
Fusobacterium nucleatum - Q8RFS6
Clostridium acetobutylicum - Q97GK4
Clostridium botulinum - A5I6L8
Campylobacter jejuni - Q9PIS3
Helicobacter pylori - P56071
Desulfovibrio vulgaris - Q728R5
Geobacter sulfurreducens - Q74D04
Leptospira interrogans - Q8F6R1
Francisella tularensis - Q5NGL8
Caulobacter crescentus - Q9AAX8
Rickettsia prowazekii - O05947
Rhodospirillum rubrum - Q2RNL4
Rhodobacter sphaeroides - Q3J0X4
Agrobacterium tumefaciens - Q8UEL1
Bordetella pertussis - Q7VY64
Burkholderia pseudomallei - Q63TM2
Coxiella burnetii - Q83C10
Neisseria gonorrhoeae - Q5F9U4
Pseudomonas aeruginosa - Q9I099
Xanthomonas campestris - Q8P7Z2
Prochlorococcus marinus - Q7VBM8
Vibrio cholerae - Q9KMN7
Haemophilus influenzae - P43014
Escherichia coli - P0A8M3
Yersinia pestis - Q8ZDW5
Buchnera aphidicola - P57225
Shewanella oneidensis - Q8EER9
Bifidobacterium longum - Q8G6C2
Streptomyces coelicolor - Q9L278
Corynebacterium glutamicum - Q8NPZ0
Mycobacterium smegmatis - A0QWG2
Mycobacterium tuberculosis - P9WFT5
Aquifex aeolicus - O67583
Borrelia burgdorferi - O51662
Mycoplasma pneumoniae - P75225
Staphylococcus aureus - Q2FXP7
Bacillus subtilis - P18255
Listeria monocytogenes - Q8Y6X2
Enterococcus faecalis - Q830D0
Lactobacillus plantarum - Q88WU9
Lactococcus lactis - Q9CED2
Streptococcus pneumoniae - Q8DNX8
Rhodopirellula baltica - Q7UJ52
Gloeobacter violaceus - Q7NCU0
Synechocystis sp - Q55806
Thermosynechococcus elongatus - Q8DGW0
Deinococcus radiodurans - Q9RSP3
Thermus thermophilus - P56881
Chlorobium tepidum - Q8KAN0
Bacteroides thetaiotaomicron - Q8AAP2
Flavobacterium psychrophilum - A6GY22
0.3

R. ValRS

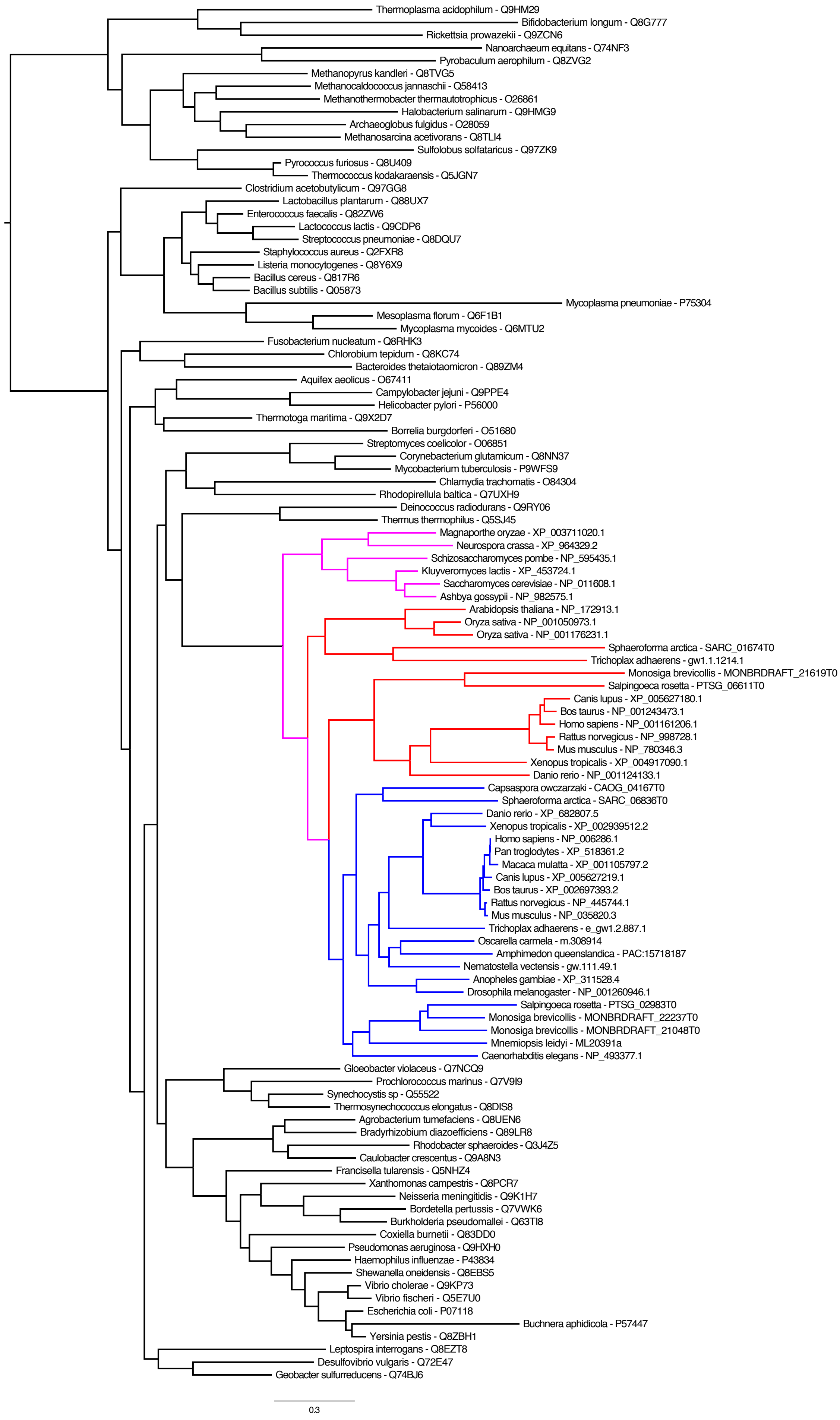
Thermoplasma acidophilum - Q9HM29
Bifidobacterium longum - Q8G777
Rickettsia prowazekii - Q9ZCN6
Nanoarchaeum equitans - Q74NF3
Pyrobaculum aerophilum - Q8ZVG2
Methanopyrus kandleri - Q8TVG5
Methanocaldococcus jannaschii - Q58413
Methanothermobacter thermautotrophicus - O26861
Halobacterium salinarum - Q9HMG9
Archaeoglobus fulgidus - O28059
Methanosarcina acetivorans - Q8TLI4
Sulfolobus solfataricus - Q97ZK9
Pyrococcus furiosus - Q8U409
Thermococcus kodakaraensis - Q5JGN7
Clostridium acetobutylicum - Q97GG8
Lactobacillus plantarum - Q88UX7
Enterococcus faecalis - Q82ZW6
Lactococcus lactis - Q9CDP6
Streptococcus pneumoniae - Q8DQU7
Staphylococcus aureus - Q2FXR8
Listeria monocytogenes - Q8Y6X9
Bacillus cereus - Q817R6
Bacillus subtilis - Q05873
Mycoplasma pneumoniae - P75304
Mesoplasma florum - Q6F1B1
Mycoplasma mycoides - Q6MTU2
Fusobacterium nucleatum - Q8RHK3
Chlorobium tepidum - Q8KC74
Bacteroides thetaiotaomicron - Q89ZM4
Aquifex aeolicus - O67411
Campylobacter jejuni - Q9PPE4
Helicobacter pylori - P56000
Thermotoga maritima - Q9X2D7
Borrelia burgdorferi - O51680
Streptomyces coelicolor - O06851
Corynebacterium glutamicum - Q8NN37
Mycobacterium tuberculosis - P9WFS9
Chlamydia trachomatis - O84304
Rhodopirellula baltica - Q7UXH9
Deinococcus radiodurans - Q9RY06
Thermus thermophilus - Q5SJ45
Magnaporthe oryzae - XP_003711020.1
Neurospora crassa - XP_964329.2
Schizosaccharomyces pombe - NP_595435.1
Kluyveromyces lactis - XP_453724.1
Saccharomyces cerevisiae - NP_011608.1
Ashbya gossypii - NP_982575.1
Arabidopsis thaliana - NP_172913.1
Oryza sativa - NP_001050973.1
Oryza sativa - NP_001176231.1
Sphaeroforma arctica - SARC_01674T0
Trichoplax adhaerens - gw1.1.1214.1
Monosiga brevicollis - MONBRDRAFT_21619T0
Salpingoeca rosetta - PTSG_06611T0
Canis lupus - XP_005627180.1
Bos taurus - NP_001243473.1
Homo sapiens - NP_001161206.1
Rattus norvegicus - NP_998728.1
Mus musculus - NP_780346.3
Xenopus tropicalis - XP_004917090.1
Danio rerio - NP_001124133.1
Capsaspora owczarzaki - CAOG_04167T0
Sphaeroforma arctica - SARC_06836T0
Danio rerio - XP_682807.5
Xenopus tropicalis - XP_002939512.2
Homo sapiens - NP_006286.1
Pan troglodytes - XP_518361.2
Macaca mulatta - XP_001105797.2
Canis lupus - XP_005627219.1
Bos taurus - XP_002697393.2
Rattus norvegicus - NP_445744.1
Mus musculus - NP_035820.3
Trichoplax adhaerens - e_gw1.2.887.1
Oscarella carmela - m.308914
Amphimedon queenslandica - PAC:15718187
Nematostella vectensis - gw.111.49.1
Anopheles gambiae - XP_311528.4
Drosophila melanogaster - NP_001260946.1
Salpingoeca rosetta - PTSG_02983T0
Monosiga brevicollis - MONBRDRAFT_22237T0
Monosiga brevicollis - MONBRDRAFT_21048T0
Mnemiopsis leidyi - ML20391a
Caenorhabditis elegans - NP_493377.1
Gloeobacter violaceus - Q7NCQ9
Prochlorococcus marinus - Q7V9I9
Synechocystis sp - Q55522
Thermosynechococcus elongatus - Q8DIS8
Agrobacterium tumefaciens - Q8UEN6
Bradyrhizobium diazoefficiens - Q89LR8
Rhodobacter sphaeroides - Q3J4Z5
Caulobacter crescentus - Q9A8N3
Francisella tularensis - Q5NHZ4
Xanthomonas campestris - Q8PCR7
Neisseria meningitidis - Q9K1H7
Bordetella pertussis - Q7VWK6
Burkholderia pseudomallei - Q63TI8
Coxiella burnetii - Q83DD0
Pseudomonas aeruginosa - Q9HXH0
Haemophilus influenzae - P43834
Shewanella oneidensis - Q8EBS5
Vibrio cholerae - Q9KP73
Vibrio fischeri - Q5E7U0
Escherichia coli - P07118
Buchnera aphidicola - P57447
Yersinia pestis - Q8ZBH1
Leptospira interrogans - Q8EZT8
Desulfovibrio vulgaris - Q72E47
Geobacter sulfurreducens - Q74BJ6
0.3

S. TrpRS

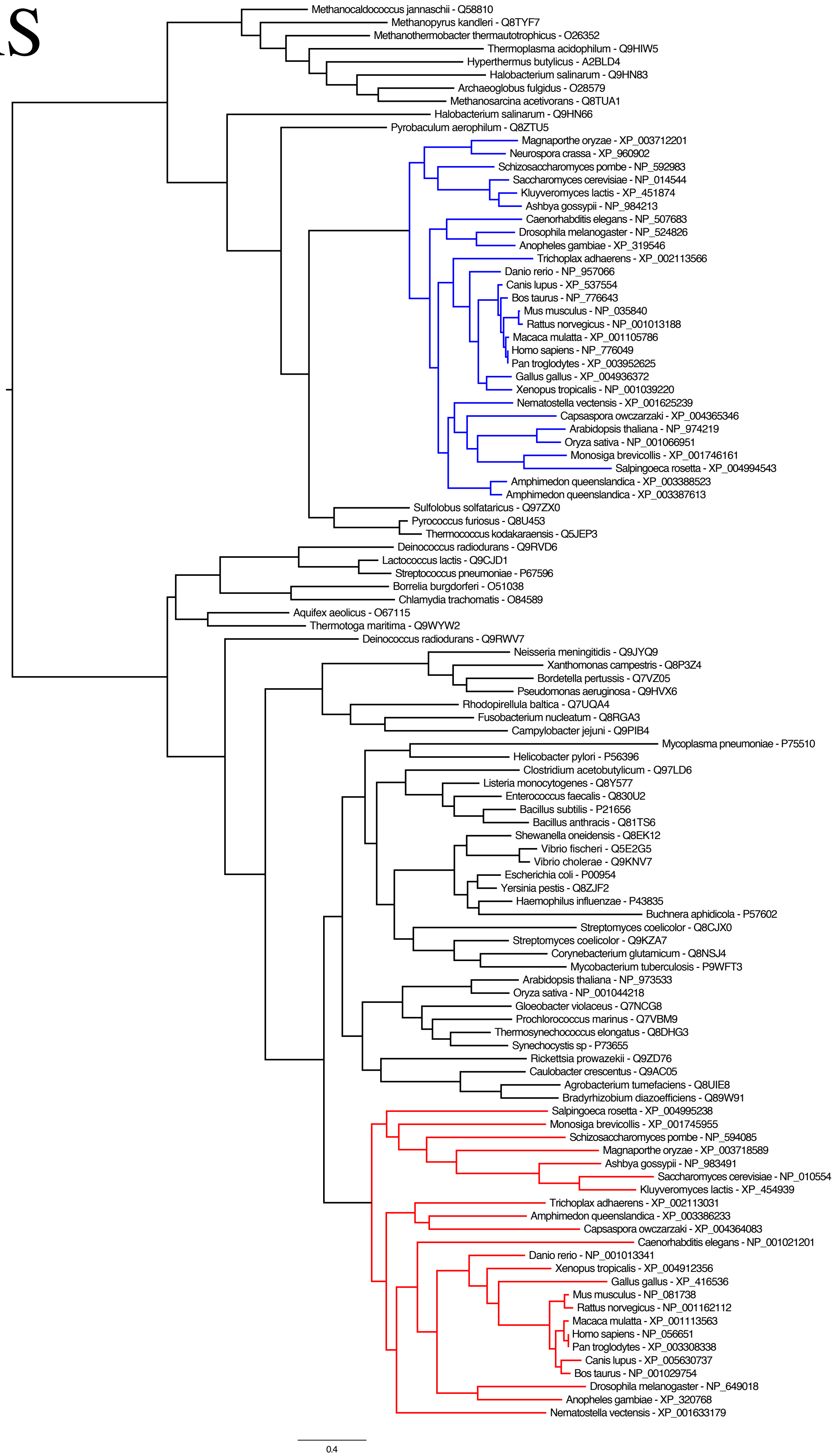

Methanocaldococcus jannaschii - Q58810
Methanopyrus kandleri - Q8TYF7
Methanothermobacter thermautotrophicus - O26352
Thermoplasma acidophilum - Q9HIW5
Hyperthermus butylicus - A2BLD4
Halobacterium salinarum - Q9HN83
Archaeoglobus fulgidus - O28579
Methanosarcina acetivorans - Q8TUA1
Halobacterium salinarum - Q9HN66
Pyrobaculum aerophilum - Q8ZTU5
Magnaporthe oryzae - XP_003712201
Neurospora crassa - XP_960902
Schizosaccharomyces pombe - NP_592983
Saccharomyces cerevisiae - NP_014544
Kluyveromyces lactis - XP_451874
Ashbya gossypii - NP_984213
Caenorhabditis elegans - NP_507683
Drosophila melanogaster - NP_524826
Anopheles gambiae - XP_319546
Trichoplax adhaerens - XP_002113566
Danio rerio - NP_957066
Canis lupus - XP_537554
Bos taurus - NP_776643
Mus musculus - NP_035840
Rattus norvegicus - NP_001013188
Macaca mulatta - XP_001105786
Homo sapiens - NP_776049
Pan troglodytes - XP_003952625
Gallus gallus - XP_004936372
Xenopus tropicalis - NP_001039220
Nematostella vectensis - XP_001625239
Capsaspora owczarzaki - XP_004365346
Arabidopsis thaliana - NP_974219
Oryza sativa - NP_001066951
Monosiga brevicollis - XP_001746161
Salpingoeca rosetta - XP_004994543
Amphimedon queenslandica - XP_003388523
Amphimedon queenslandica - XP_003387613
Sulfolobus solfataricus - Q97ZX0
Pyrococcus furiosus - Q8U453
Thermococcus kodakaraensis - Q5JEP3
Deinococcus radiodurans - Q9RVD6
Lactococcus lactis - Q9CJD1
Streptococcus pneumoniae - P67596
Borrelia burgdorferi - O51038
Chlamydia trachomatis - O84589
Aquifex aeolicus - O67115
Thermotoga maritima - Q9WYW2
Deinococcus radiodurans - Q9RWV7
Neisseria meningitidis - Q9JYQ9
Xanthomonas campestris - Q8P3Z4
Bordetella pertussis - Q7VZ05
Pseudomonas aeruginosa - Q9HVX6
Rhodopirellula baltica - Q7UQA4
Fusobacterium nucleatum - Q8RGA3
Campylobacter jejuni - Q9PIB4
Mycoplasma pneumoniae - P75510
Helicobacter pylori - P56396
Clostridium acetobutylicum - Q97LD6
Listeria monocytogenes - Q8Y577
Enterococcus faecalis - Q830U2
Bacillus subtilis - P21656
Bacillus anthracis - Q81TS6
Shewanella oneidensis - Q8EK12
Vibrio fischeri - Q5E2G5
Vibrio cholerae - Q9KNV7
Escherichia coli - P00954
Yersinia pestis - Q8ZJF2
Haemophilus influenzae - P43835
Buchnera aphidicola - P57602
Streptomyces coelicolor - Q8CJX0
Streptomyces coelicolor - Q9KZA7
Corynebacterium glutamicum - Q8NSJ4
Mycobacterium tuberculosis - P9WFT3
Arabidopsis thaliana - NP_973533
Oryza sativa - NP_001044218
Gloeobacter violaceus - Q7NCG8
Prochlorococcus marinus - Q7VBM9
Thermosynechococcus elongatus - Q8DHG3
Synechocystis sp - P73655
Rickettsia prowazekii - Q9ZD76
Caulobacter crescentus - Q9AC05
Agrobacterium tumefaciens - Q8UIE8
Bradyrhizobium diazoefficiens - Q89W91
Salpingoeca rosetta - XP_004995238
Monosiga brevicollis - XP_001745955
Schizosaccharomyces pombe - NP_594085
Magnaporthe oryzae - XP_003718589
Ashbya gossypii - NP_983491
Saccharomyces cerevisiae - NP_010554
Kluyveromyces lactis - XP_454939
Trichoplax adhaerens - XP_002113031
Amphimedon queenslandica - XP_003386233
Capsaspora owczarzaki - XP_004364083
Caenorhabditis elegans - NP_001021201
Danio rerio - NP_001013341
Xenopus tropicalis - XP_004912356
Gallus gallus - XP_416536
Mus musculus - NP_081738
Rattus norvegicus - NP_001162112
Macaca mulatta - XP_001113563
Homo sapiens - NP_056651
Pan troglodytes - XP_003308338
Canis lupus - XP_005630737
Bos taurus - NP_001029754
Drosophila melanogaster - NP_649018
Anopheles gambiae - XP_320768
Nematostella vectensis - XP_001633179
0.4

T. TyrRS

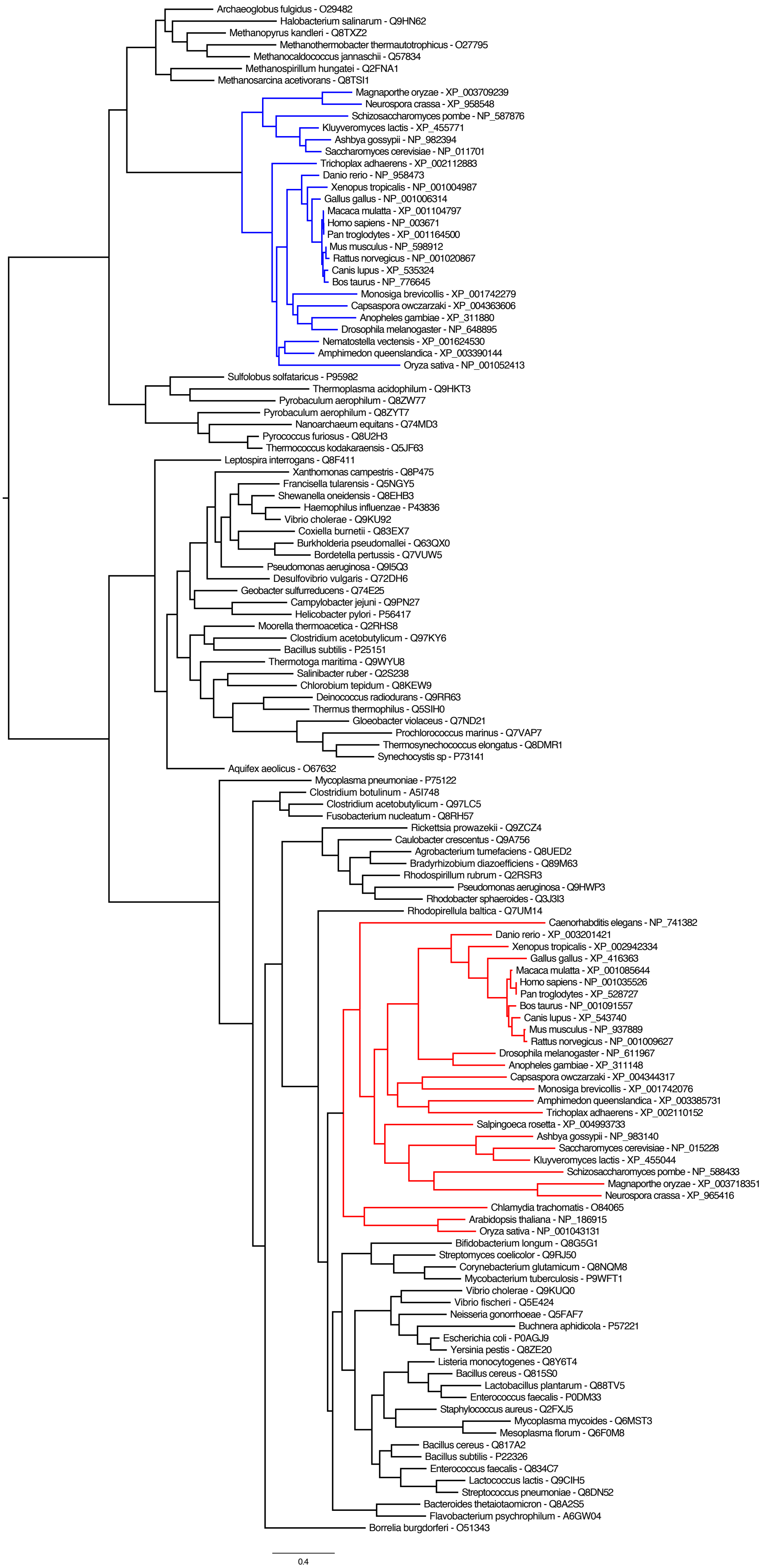
Archaeoglobus fulgidus - O29482
Halobacterium salinarum - Q9HN62
Methanopyrus kandleri - Q8TXZ2
Methanothermobacter thermautotrophicus - O27795
Methanocaldococcus jannaschii - Q57834
Methanospirillum hungatei - Q2FNA1
Methanosarcina acetivorans - Q8TSI1
Magnaporthe oryzae - XP_003709239
Neurospora crassa - XP_958548
Schizosaccharomyces pombe - NP_587876
Kluyveromyces lactis - XP_455771
Ashbya gossypii - NP_982394
Saccharomyces cerevisiae - NP_011701
Trichoplax adhaerens - XP_002112883
Danio rerio - NP_958473
Xenopus tropicalis - NP_001004987
Gallus gallus - NP_001006314
Macaca mulatta - XP_001104797
Homo sapiens - NP_003671
Pan troglodytes - XP_001164500
Mus musculus - NP_598912
Rattus norvegicus - NP_001020867
Canis lupus - XP_535324
Bos taurus - NP_776645
Monosiga brevicollis - XP_001742279
Capsaspora owczarzaki - XP_004363606
Anopheles gambiae - XP_311880
Drosophila melanogaster - NP_648895
Nematostella vectensis - XP_001624530
Amphimedon queenslandica - XP_003390144
Oryza sativa - NP_001052413
Sulfolobus solfataricus - P95982
Thermoplasma acidophilum - Q9HKT3
Pyrobaculum aerophilum - Q8ZW77
Pyrobaculum aerophilum - Q8ZYT7
Nanoarchaeum equitans - Q74MD3
Pyrococcus furiosus - Q8U2H3
Thermococcus kodakaraensis - Q5JF63
Leptospira interrogans - Q8F411
Xanthomonas campestris - Q8P475
Francisella tularensis - Q5NGY5
Shewanella oneidensis - Q8EHB3
Haemophilus influenzae - P43836
Vibrio cholerae - Q9KU92
Coxiella burnetii - Q83EX7
Burkholderia pseudomallei - Q63QX0
Bordetella pertussis - Q7VUW5
Pseudomonas aeruginosa - Q9I5Q3
Desulfovibrio vulgaris - Q72DH6
Geobacter sulfurreducens - Q74E25
Campylobacter jejuni - Q9PN27
Helicobacter pylori - P56417
Moorella thermoacetica - Q2RHS8
Clostridium acetobutylicum - Q97KY6
Bacillus subtilis - P25151
Thermotoga maritima - Q9WYU8
Salinibacter ruber - Q2S238
Chlorobium tepidum - Q8KEW9
Deinococcus radiodurans - Q9RR63
Thermus thermophilus - Q5SIH0
Gloeobacter violaceus - Q7ND21
Prochlorococcus marinus - Q7VAP7
Thermosynechococcus elongatus - Q8DMR1
Synechocystis sp - P73141
Aquifex aeolicus - O67632
Mycoplasma pneumoniae - P75122
Clostridium botulinum - A5I748
Clostridium acetobutylicum - Q97LC5
Fusobacterium nucleatum - Q8RH57
Rickettsia prowazekii - Q9ZCZ4
Caulobacter crescentus - Q9A756
Agrobacterium tumefaciens - Q8UED2
Bradyrhizobium diazoefficiens - Q89M63
Rhodospirillum rubrum - Q2RSR3
Pseudomonas aeruginosa - Q9HWP3
Rhodobacter sphaeroides - Q3J3I3
Rhodopirellula baltica - Q7UM14
Caenorhabditis elegans - NP_741382
Danio rerio - XP_003201421
Xenopus tropicalis - XP_002942334
Gallus gallus - XP_416363
Macaca mulatta - XP_001085644
Homo sapiens - NP_001035526
Pan troglodytes - XP_528727
Bos taurus - NP_001091557
Canis lupus - XP_543740
Mus musculus - NP_937889
Rattus norvegicus - NP_001009627
Drosophila melanogaster - NP_611967
Anopheles gambiae - XP_311148
Capsaspora owczarzaki - XP_004344317
Monosiga brevicollis - XP_001742076
Amphimedon queenslandica - XP_003385731
Trichoplax adhaerens - XP_002110152
Salpingoeca rosetta - XP_004993733
Ashbya gossypii - NP_983140
Saccharomyces cerevisiae - NP_015228
Kluyveromyces lactis - XP_455044
Schizosaccharomyces pombe - NP_588433
Magnaporthe oryzae - XP_003718351
Neurospora crassa - XP_965416
Chlamydia trachomatis - O84065
Arabidopsis thaliana - NP_186915
Oryza sativa - NP_001043131
Bifidobacterium longum - Q8G5G1
Streptomyces coelicolor - Q9RJ50
Corynebacterium glutamicum - Q8NQM8
Mycobacterium tuberculosis - P9WFT1
Vibrio cholerae - Q9KUQ0
Vibrio fischeri - Q5E424
Neisseria gonorrhoeae - Q5FAF7
Buchnera aphidicola - P57221
Escherichia coli - P0AGJ9
Yersinia pestis - Q8ZE20
Listeria monocytogenes - Q8Y6T4
Bacillus cereus - Q815S0
Lactobacillus plantarum - Q88TV5
Enterococcus faecalis - P0DM33
Staphylococcus aureus - Q2FXJ5
Mycoplasma mycoides - Q6MST3
Mesoplasma florum - Q6F0M8
Bacillus cereus - Q817A2
Bacillus subtilis - P22326
Enterococcus faecalis - Q834C7
Lactococcus lactis - Q9CIH5
Streptococcus pneumoniae - Q8DN52
Bacteroides thetaiotaomicron - Q8A2S5
Flavobacterium psychrophilum - A6GW04
Borrelia burgdorferi - O51343
0.4

U. GatA

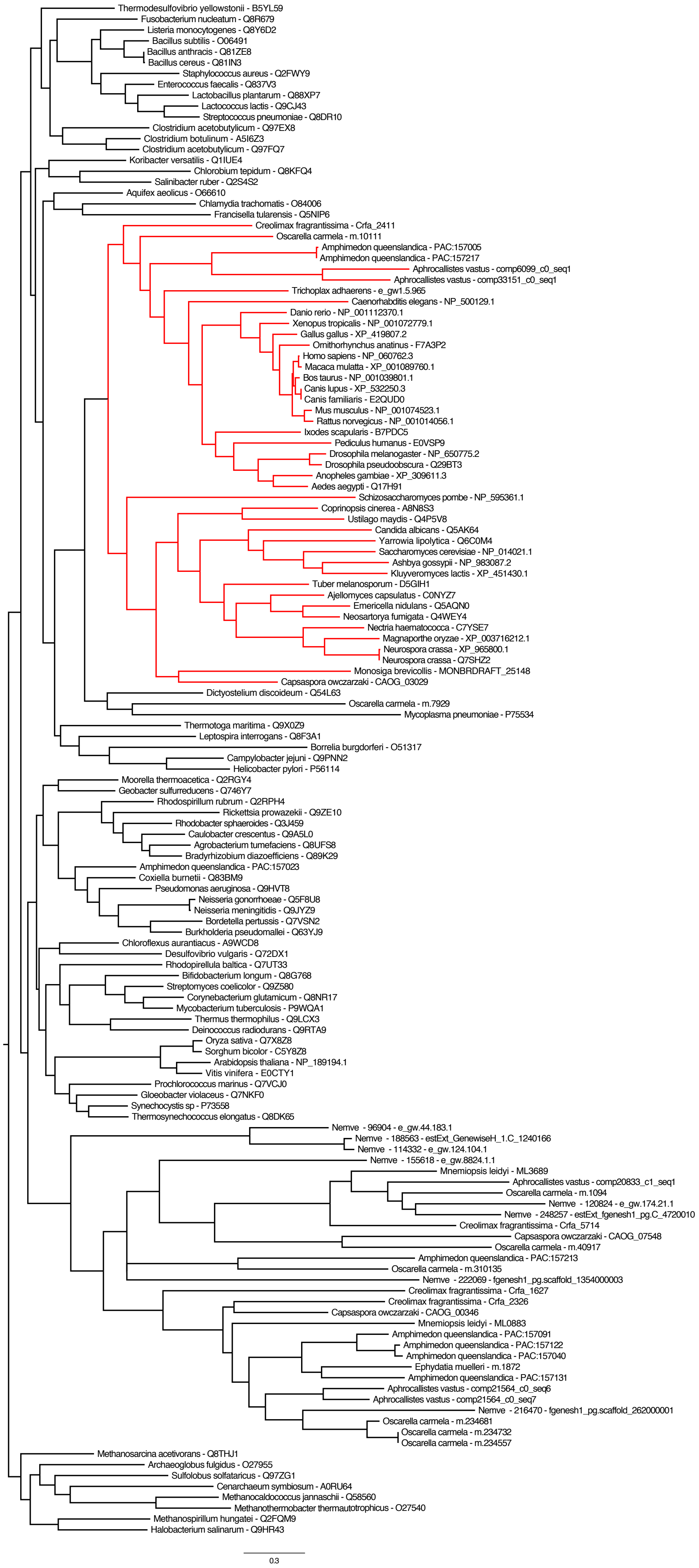
Thermodesulfovibrio yellowstonii - B5YL59
Fusobacterium nucleatum - Q8R679
Listeria monocytogenes - Q8Y6D2
Bacillus subtilis - O06491
Bacillus anthracis - Q81ZE8
Bacillus cereus - Q81IN3
Staphylococcus aureus - Q2FWY9
Enterococcus faecalis - Q837V3
Lactobacillus plantarum - Q88XP7
Lactococcus lactis - Q9CJ43
Streptococcus pneumoniae - Q8DR10
Clostridium acetobutylicum - Q97EX8
Clostridium botulinum - A5I6Z3
Clostridium acetobutylicum - Q97FQ7
Koribacter versatilis - Q1IUE4
Chlorobium tepidum - Q8KFQ4
Salinibacter ruber - Q2S4S2
Aquifex aeolicus - O66610
Chlamydia trachomatis - O84006
Francisella tularensis - Q5NIP6
Creolimax fragrantissima - Crfa_2411
Oscarella carmela - m.10111
Amphimedon queenslandica - PAC:157005
Amphimedon queenslandica - PAC:157217
Aphrocallistes vastus - comp6099_c0_seq1
Aphrocallistes vastus - comp33151_c0_seq1
Trichoplax adhaerens - e_gw1.5.965
Caenorhabditis elegans - NP_500129.1
Danio rerio - NP_001112370.1
Xenopus tropicalis - NP_001072779.1
Gallus gallus - XP_419807.2
Ornithorhynchus anatinus - F7A3P2
Homo sapiens - NP_060762.3
Macaca mulatta - XP_001089760.1
Bos taurus - NP_001039801.1
Canis lupus - XP_532250.3
Canis familiaris - E2QUD0
Mus musculus - NP_001074523.1
Rattus norvegicus - NP_001014056.1
Ixodes scapularis - B7PDC5
Pediculus humanus - E0VSP9
Drosophila melanogaster - NP_650775.2
Drosophila pseudoobscura - Q29BT3
Anopheles gambiae - XP_309611.3
Aedes aegypti - Q17H91
Schizosaccharomyces pombe - NP_595361.1
Coprinopsis cinerea - A8N8S3
Ustilago maydis - Q4P5V8
Candida albicans - Q5AK64
Yarrowia lipolytica - Q6C0M4
Saccharomyces cerevisiae - NP_014021.1
Ashbya gossypii - NP_983087.2
Kluyveromyces lactis - XP_451430.1
Tuber melanosporum - D5GIH1
Ajellomyces capsulatus - C0NYZ7
Emericella nidulans - Q5AQN0
Neosartorya fumigata - Q4WEY4
Nectria haematococca - C7YSE7
Magnaporthe oryzae - XP_003716212.1
Neurospora crassa - XP_965800.1
Neurospora crassa - Q7SHZ2
Monosiga brevicollis - MONBRDRAFT_25148
Capsaspora owczarzaki - CAOG_03029
Dictyostelium discoideum - Q54L63
Oscarella carmela - m.7929
Mycoplasma pneumoniae - P75534
Thermotoga maritima - Q9X0Z9
Leptospira interrogans - Q8F3A1
Borrelia burgdorferi - O51317
Campylobacter jejuni - Q9PNN2
Helicobacter pylori - P56114
Moorella thermoacetica - Q2RGY4
Geobacter sulfurreducens - Q746Y7
Rhodospirillum rubrum - Q2RPH4
Rickettsia prowazekii - Q9ZE10
Rhodobacter sphaeroides - Q3J459
Caulobacter crescentus - Q9A5L0
Agrobacterium tumefaciens - Q8UFS8
Bradyrhizobium diazoefficiens - Q89K29
Amphimedon queenslandica - PAC:157023
Coxiella burnetii - Q83BM9
Pseudomonas aeruginosa - Q9HVT8
Neisseria gonorrhoeae - Q5F8U8
Neisseria meningitidis - Q9JYZ9
Bordetella pertussis - Q7VSN2
Burkholderia pseudomallei - Q63YJ9
Chloroflexus aurantiacus - A9WCD8
Desulfovibrio vulgaris - Q72DX1
Rhodopirellula baltica - Q7UT33
Bifidobacterium longum - Q8G768
Streptomyces coelicolor - Q9Z580
Corynebacterium glutamicum - Q8NR17
Mycobacterium tuberculosis - P9WQA1
Thermus thermophilus - Q9LCX3
Deinococcus radiodurans - Q9RTA9
Oryza sativa - Q7X8Z8
Sorghum bicolor - C5Y8Z8
Arabidopsis thaliana - NP_189194.1
Vitis vinifera - E0CTY1
Prochlorococcus marinus - Q7VCJ0
Gloeobacter violaceus - Q7NKF0
Synechocystis sp - P73558
Thermosynechococcus elongatus - Q8DK65
Nemve - 96904 - e_gw.44.183.1
Nemve - 188563 - estExt_GenewiseH_1.C_1240166
Nemve - 114332 - e_gw.124.104.1
Nemve - 155618 - e_gw.8824.1.1
Mnemiopsis leidyi - ML3689
Aphrocallistes vastus - comp20833_c1_seq1
Oscarella carmela - m.1094
Nemve - 120824 - e_gw.174.21.1
Nemve - 248257 - estExt_fgenesh1_pg.C_4720010
Creolimax fragrantissima - Crfa_5714
Capsaspora owczarzaki - CAOG_07548
Oscarella carmela - m.40917
Amphimedon queenslandica - PAC:157213
Oscarella carmela - m.310135
Nemve - 222069 - fgenesh1_pg.scaffold_1354000003
Creolimax fragrantissima - Crfa_1627
Creolimax fragrantissima - Crfa_2326
Capsaspora owczarzaki - CAOG_00346
Mnemiopsis leidyi - ML0883
Amphimedon queenslandica - PAC:157091
Amphimedon queenslandica - PAC:157122
Amphimedon queenslandica - PAC:157040
Ephydatia muelleri - m.1872
Amphimedon queenslandica - PAC:157131
Aphrocallistes vastus - comp21564_c0_seq6
Aphrocallistes vastus - comp21564_c0_seq7
Nemve - 216470 - fgenesh1_pg.scaffold_262000001
Oscarella carmela - m.234681
Oscarella carmela - m.234732
Oscarella carmela - m.234557
Methanosarcina acetivorans - Q8THJ1
Archaeoglobus fulgidus - O27955
Sulfolobus solfataricus - Q97ZG1
Cenarchaeum symbiosum - A0RU64
Methanocaldococcus jannaschii - Q58560
Methanothermobacter thermautotrophicus - O27540
Methanospirillum hungatei - Q2FQM9
Halobacterium salinarum - Q9HR43
0.3

V. GatB

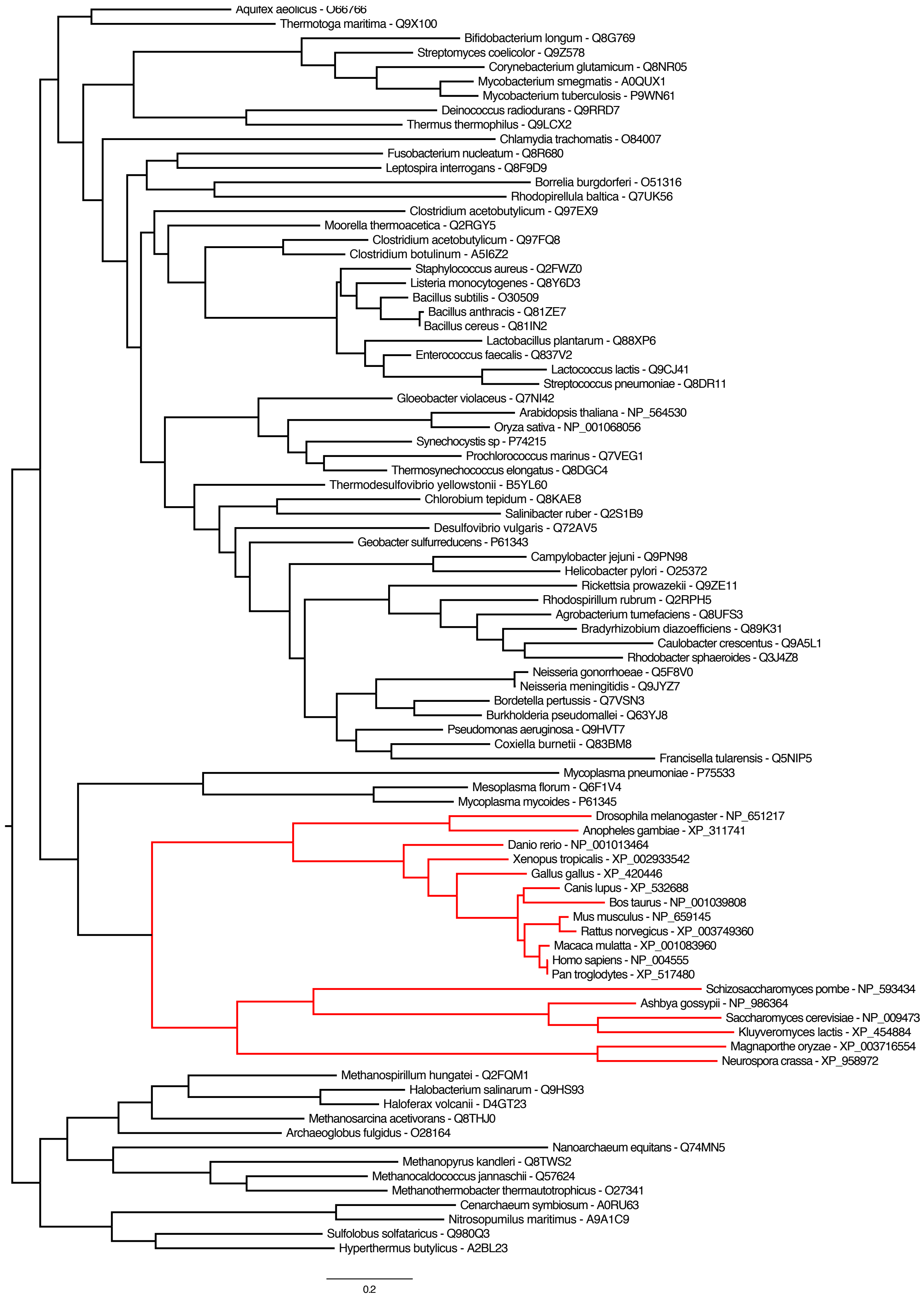
Aquifex aeolicus - O66766
Thermotoga maritima - Q9X100
Bifidobacterium longum - Q8G769
Streptomyces coelicolor - Q9Z578
Corynebacterium glutamicum - Q8NR05
Mycobacterium smegmatis - A0QUX1
Mycobacterium tuberculosis - P9WN61
Deinococcus radiodurans - Q9RRD7
Thermus thermophilus - Q9LCX2
Chlamydia trachomatis - O84007
Fusobacterium nucleatum - Q8R680
Leptospira interrogans - Q8F9D9
Borrelia burgdorferi - O51316
Rhodopirellula baltica - Q7UK56
Clostridium acetobutylicum - Q97EX9
Moorella thermoacetica - Q2RGY5
Clostridium acetobutylicum - Q97FQ8
Clostridium botulinum - A5I6Z2
Staphylococcus aureus - Q2FWZ0
Listeria monocytogenes - Q8Y6D3
Bacillus subtilis - O30509
Bacillus anthracis - Q81ZE7
Bacillus cereus - Q81IN2
Lactobacillus plantarum - Q88XP6
Enterococcus faecalis - Q837V2
Lactococcus lactis - Q9CJ41
Streptococcus pneumoniae - Q8DR11
Gloeobacter violaceus - Q7NI42
Arabidopsis thaliana - NP_564530
Oryza sativa - NP_001068056
Synechocystis sp - P74215
Prochlorococcus marinus - Q7VEG1
Thermosynechococcus elongatus - Q8DGC4
Thermodesulfovibrio yellowstonii - B5YL60
Chlorobium tepidum - Q8KAE8
Salinibacter ruber - Q2S1B9
Desulfovibrio vulgaris - Q72AV5
Geobacter sulfurreducens - P61343
Campylobacter jejuni - Q9PN98
Helicobacter pylori - O25372
Rickettsia prowazekii - Q9ZE11
Rhodospirillum rubrum - Q2RPH5
Agrobacterium tumefaciens - Q8UFS3
Bradyrhizobium diazoefficiens - Q89K31
Caulobacter crescentus - Q9A5L1
Rhodobacter sphaeroides - Q3J4Z8
Neisseria gonorrhoeae - Q5F8V0
Neisseria meningitidis - Q9JYZ7
Bordetella pertussis - Q7VSN3
Burkholderia pseudomallei - Q63YJ8
Pseudomonas aeruginosa - Q9HVT7
Coxiella burnetii - Q83BM8
Francisella tularensis - Q5NIP5
Mycoplasma pneumoniae - P75533
Mesoplasma florum - Q6F1V4
Mycoplasma mycoides - P61345
Drosophila melanogaster - NP_651217
Anopheles gambiae - XP_311741
Danio rerio - NP_001013464
Xenopus tropicalis - XP_002933542
Gallus gallus - XP_420446
Canis lupus - XP_532688
Bos taurus - NP_001039808
Mus musculus - NP_659145
Rattus norvegicus - XP_003749360
Macaca mulatta - XP_001083960
Homo sapiens - NP_004555
Pan troglodytes - XP_517480
Schizosaccharomyces pombe - NP_593434
Ashbya gossypii - NP_986364
Saccharomyces cerevisiae - NP_009473
Kluyveromyces lactis - XP_454884
Magnaporthe oryzae - XP_003716554
Neurospora crassa - XP_958972
Methanospirillum hungatei - Q2FQM1
Halobacterium salinarum - Q9HS93
Haloferax volcanii - D4GT23
Methanosarcina acetivorans - Q8THJ0
Archaeoglobus fulgidus - O28164
Nanoarchaeum equitans - Q74MN5
Methanopyrus kandleri - Q8TWS2
Methanocaldococcus jannaschii - Q57624
Methanothermobacter thermautotrophicus - O27341
Cenarchaeum symbiosum - A0RU63
Nitrosopumilus maritimus - A9A1C9
Sulfolobus solfataricus - Q980Q3
Hyperthermus butylicus - A2BL23
0.2

W. TilS

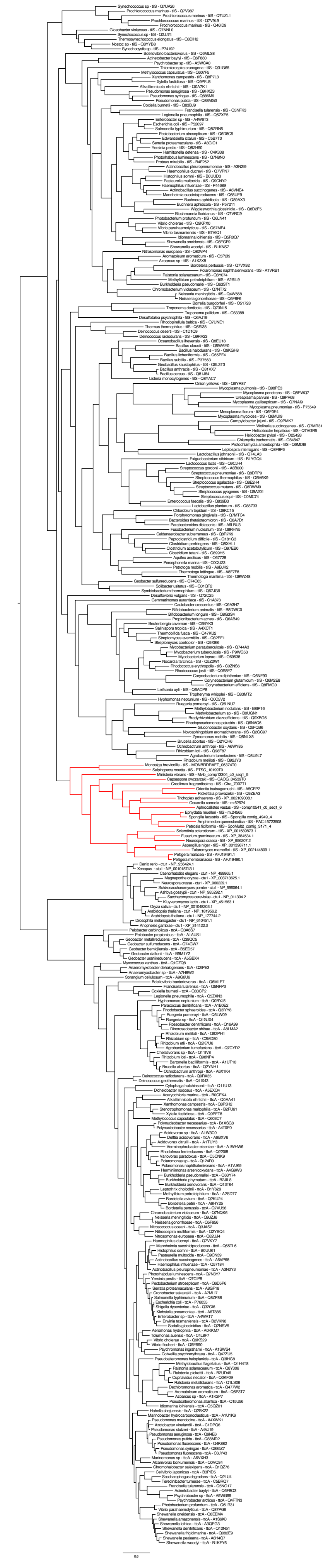

Supplementary Figure S2. Bayesian phylogenetic analysis of aaRS sequences from families with a mitochondrial lineage emerging after the divergence of eukaryotes. Trees were estimated jointly using the multigene version of PhyloBayes MPI with the CAT+WAG+Γ amino acid substitution model pooled across alignments. Two chains were run for 10,000 generations each. Convergence was assessed using the `bpcomp` program, ensuring that the `maxdiff` statistic was less than 0.3 for all alignments. **A.** CysRS, **B.** LysRS, **C.** ThrRS, **D.** ValRS

A. CysRS

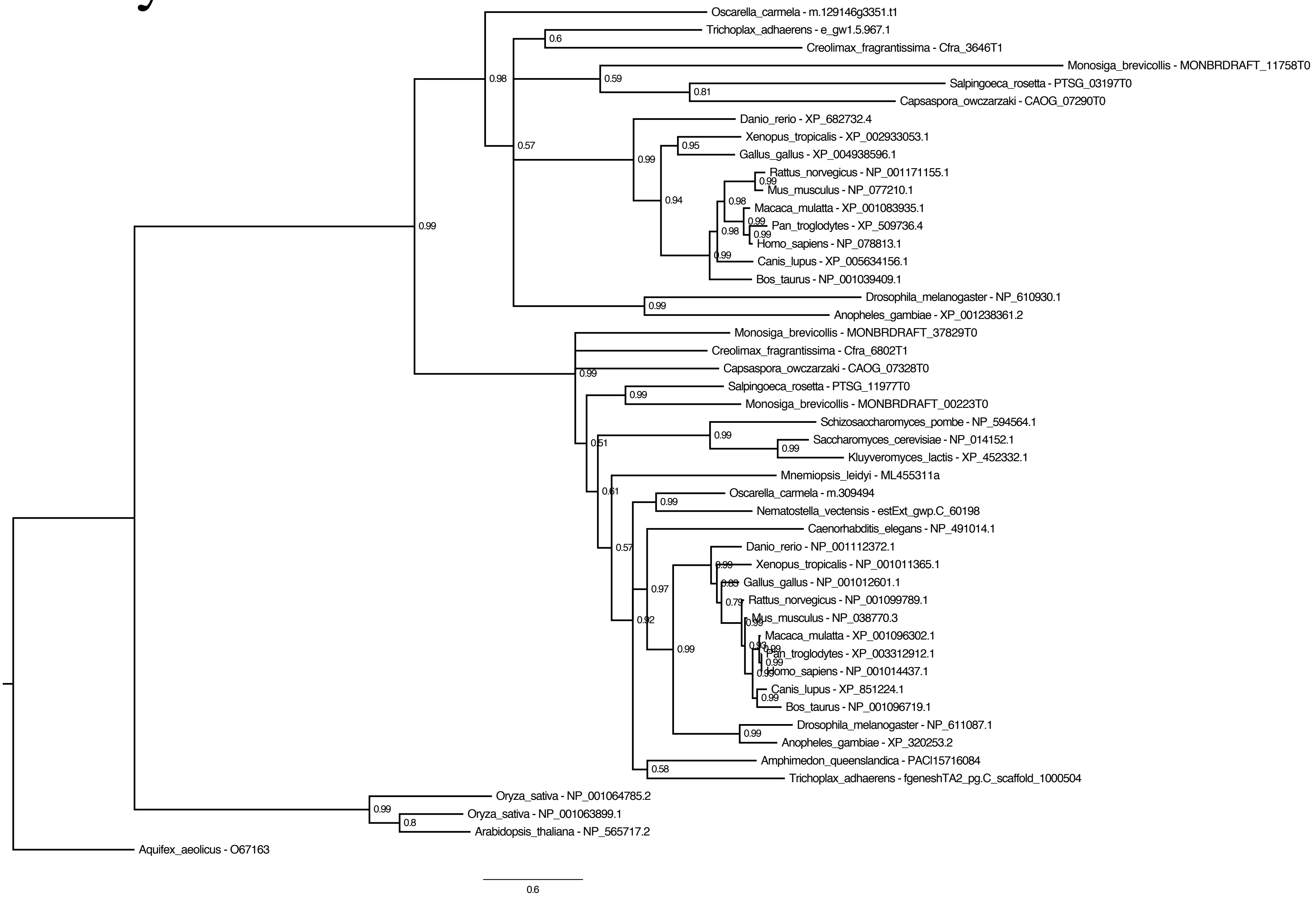

Oscarella_carmela - m.129146g3351.t1
Trichoplax_adhaerens - e_gw1.5.967.1
Creolimax_fragrantissima - Cfra_3646T1
Monosiga_brevicollis - MONBRDRAFT_11758T0
Salpingoeca_rosetta - PTSG_03197T0
Capsaspora_owczarzaki - CAOG_07290T0
Danio_rerio - XP_682732.4
Xenopus_tropicalis - XP_002933053.1
Gallus_gallus - XP_004938596.1
Rattus_norvegicus - NP_001171155.1
Mus_musculus - NP_077210.1
Macaca_mulatta - XP_001083935.1
Pan_troglodytes - XP_509736.4
Homo_sapiens - NP_078813.1
Canis_lupus - XP_005634156.1
Bos_taurus - NP_001039409.1
Drosophila_melanogaster - NP_610930.1
Anopheles_gambiae - XP_001238361.2
Monosiga_brevicollis - MONBRDRAFT_37829T0
Creolimax_fragrantissima - Cfra_6802T1
Capsaspora_owczarzaki - CAOG_07328T0
Salpingoeca_rosetta - PTSG_11977T0
Monosiga_brevicollis - MONBRDRAFT_00223T0
Schizosaccharomyces_pombe - NP_594564.1
Saccharomyces_cerevisiae - NP_014152.1
Kluyveromyces_lactis - XP_452332.1
Mnemiopsis_leidyi - ML455311a
Oscarella_carmela - m.309494
Nematostella_vectensis - estExt_gwp.C_60198
Caenorhabditis_elegans - NP_491014.1
Danio_rerio - NP_001112372.1
Xenopus_tropicalis - NP_001011365.1
Gallus_gallus - NP_001012601.1
Rattus_norvegicus - NP_001099789.1
Mus_musculus - NP_038770.3
Macaca_mulatta - XP_001096302.1
Pan_troglodytes - XP_003312912.1
Homo_sapiens - NP_001014437.1
Canis_lupus - XP_851224.1
Bos_taurus - NP_001096719.1
Drosophila_melanogaster - NP_611087.1
Anopheles_gambiae - XP_320253.2
Amphimedon_queenslandica - PACI15716084
Trichoplax_adhaerens - fgeneshTA2_pg.C_scaffold_1000504
Oryza_sativa - NP_001064785.2
Oryza_sativa - NP_001063899.1
Arabidopsis_thaliana - NP_565717.2
Aquifex_aeolicus - O67163
0.6

B. LysRS

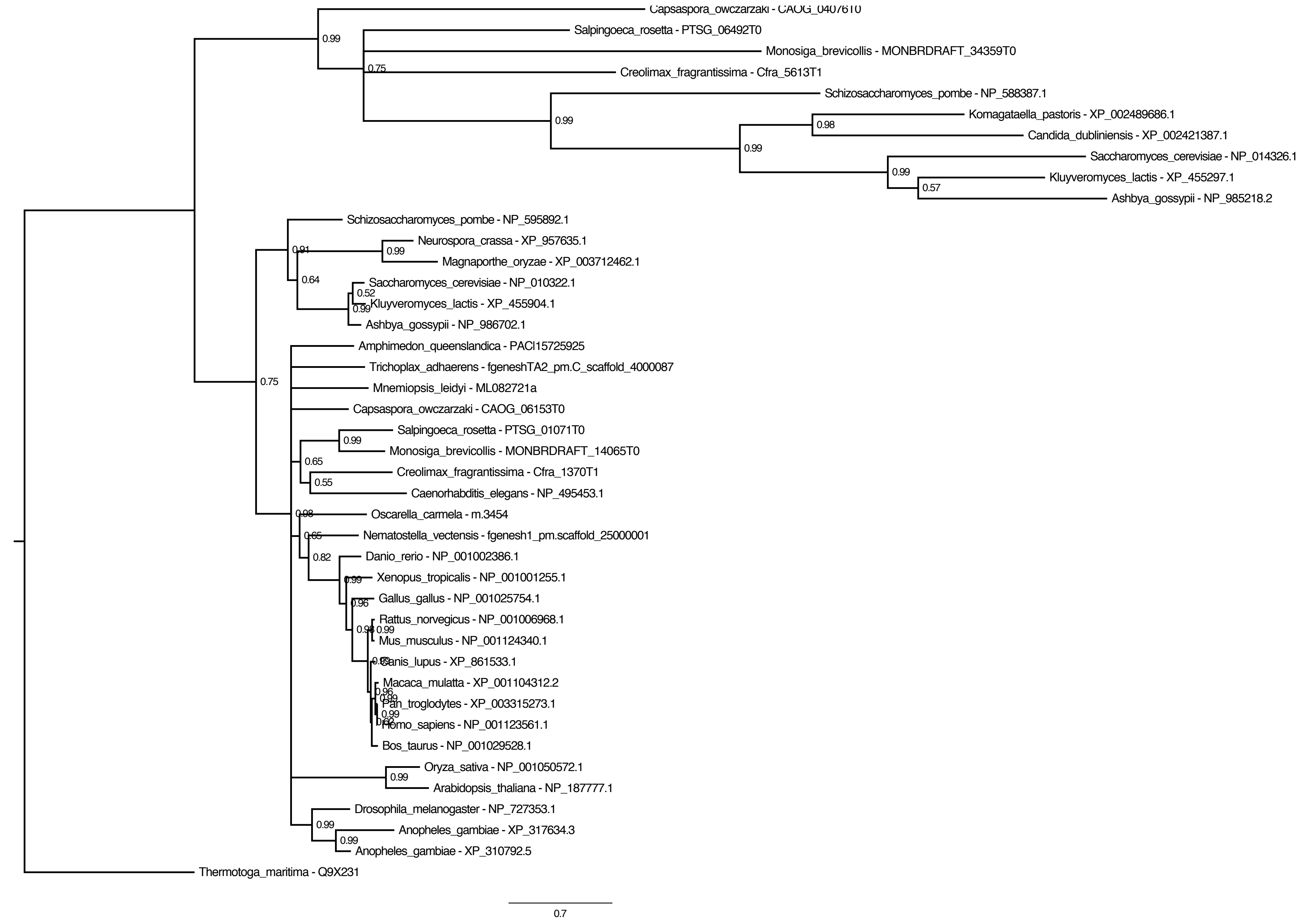

Capsaspora_owczarzaki - CAOG_04076T0
Salpingoeca_rosetta - PTSG_06492T0
Monosiga_brevicollis - MONBRDRAFT_34359T0
Creolimax_fragrantissima - Cfra_5613T1
Schizosaccharomyces_pombe - NP_588387.1
Komagataella_pastoris - XP_002489686.1
Candida_dubliniensis - XP_002421387.1
Saccharomyces_cerevisiae - NP_014326.1
Kluyveromyces_lactis - XP_455297.1
Ashbya_gossypii - NP_985218.2
Schizosaccharomyces_pombe - NP_595892.1
Neurospora_crassa - XP_957635.1
Magnaporthe_oryzae - XP_003712462.1
Saccharomyces_cerevisiae - NP_010322.1
Kluyveromyces_lactis - XP_455904.1
Ashbya_gossypii - NP_986702.1
Amphimedon_queenslandica - PACI15725925
Trichoplax_adhaerens - fgeneshTA2_pm.C_scaffold_4000087
Mnemiopsis_leidyi - ML082721a
Capsaspora_owczarzaki - CAOG_06153T0
Salpingoeca_rosetta - PTSG_01071T0
Monosiga_brevicollis - MONBRDRAFT_14065T0
Creolimax_fragrantissima - Cfra_1370T1
Caenorhabditis_elegans - NP_495453.1
Oscarella_carmela - m.3454
Nematostella_vectensis - fgenesh1_pm.scaffold_25000001
Danio_rerio - NP_001002386.1
Xenopus_tropicalis - NP_001001255.1
Gallus_gallus - NP_001025754.1
Rattus_norvegicus - NP_001006968.1
Mus_musculus - NP_001124340.1
Canis_lupus - XP_861533.1
Macaca_mulatta - XP_001104312.2
Pan_troglodytes - XP_003315273.1
Homo_sapiens - NP_001123561.1
Bos_taurus - NP_001029528.1
Oryza_sativa - NP_001050572.1
Arabidopsis_thaliana - NP_187777.1
Drosophila_melanogaster - NP_727353.1
Anopheles_gambiae - XP_317634.3
Anopheles_gambiae - XP_310792.5
Thermotoga_maritima - Q9X231
0.99
0.75
0.99
0.98
0.99
0.99
0.57
0.91
0.99
0.64
0.52
0.99
0.75
0.99
0.65
0.55
0.98
0.65
0.82
0.99
0.96
0.99
0.99
0.99
0.7

C. ThrRS

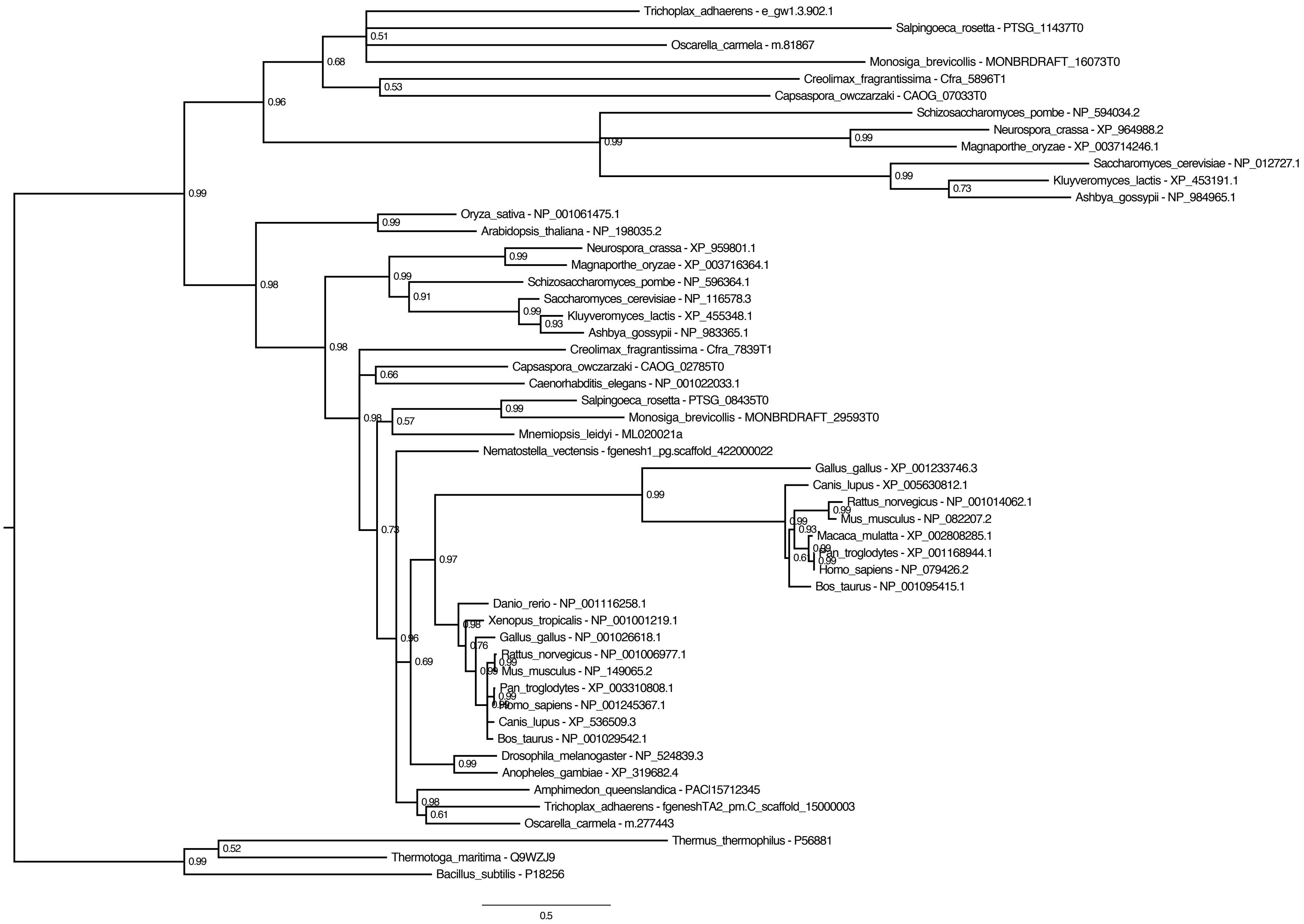
Trichoplax_adhaerens - e_gw1.3.902.1
Salpingoeca_rosetta - PTSG_11437T0
Oscarella_carmela - m.81867
Monosiga_brevicollis - MONBRDRAFT_16073T0
Creolimax_fragrantissima - Cfra_5896T1
Capsaspora_owczarzaki - CAOG_07033T0
Schizosaccharomyces_pombe - NP_594034.2
Neurospora_crassa - XP_964988.2
Magnaporthe_oryzae - XP_003714246.1
Saccharomyces_cerevisiae - NP_012727.1
Kluyveromyces_lactis - XP_453191.1
Ashbya_gossypii - NP_984965.1
Oryza_sativa - NP_001061475.1
Arabidopsis_thaliana - NP_198035.2
Neurospora_crassa - XP_959801.1
Magnaporthe_oryzae - XP_003716364.1
Schizosaccharomyces_pombe - NP_596364.1
Saccharomyces_cerevisiae - NP_116578.3
Kluyveromyces_lactis - XP_455348.1
Ashbya_gossypii - NP_983365.1
Creolimax_fragrantissima - Cfra_7839T1
Capsaspora_owczarzaki - CAOG_02785T0
Caenorhabditis_elegans - NP_001022033.1
Salpingoeca_rosetta - PTSG_08435T0
Monosiga_brevicollis - MONBRDRAFT_29593T0
Mnemiopsis_leidyi - ML020021a
Nematostella_vectensis - fgenesh1_pg.scaffold_422000022
Gallus_gallus - XP_001233746.3
Canis_lupus - XP_005630812.1
Rattus_norvegicus - NP_001014062.1
Mus_musculus - NP_082207.2
Macaca_mulatta - XP_002808285.1
Pan_troglodytes - XP_001168944.1
Homo_sapiens - NP_079426.2
Bos_taurus - NP_001095415.1
Danio_rerio - NP_001116258.1
Xenopus_tropicalis - NP_001001219.1
Gallus_gallus - NP_001026618.1
Rattus_norvegicus - NP_001006977.1
Mus_musculus - NP_149065.2
Pan_troglodytes - XP_003310808.1
Homo_sapiens - NP_001245367.1
Canis_lupus - XP_536509.3
Bos_taurus - NP_001029542.1
Drosophila_melanogaster - NP_524839.3
Anopheles_gambiae - XP_319682.4
Amphimedon_queenslandica - PACl15712345
Trichoplax_adhaerens - fgeneshTA2_pm.C_scaffold_15000003
Oscarella_carmela - m.277443
Thermus_thermophilus - P56881
Thermotoga_maritima - Q9WZJ9
Bacillus_subtilis - P18256
0.51
0.68
0.53
0.96
0.99
0.99
0.99
0.73
0.99
0.99
0.98
0.99
0.99
0.91
0.99
0.93
0.98
0.66
0.99
0.98
0.57
0.73
0.99
0.97
0.99
0.93
0.99
0.99
0.61
0.99
0.98
0.76
0.96
0.99
0.99
0.69
0.99
0.99
0.99
0.98
0.61
0.52
0.99
0.5

D. ValRS

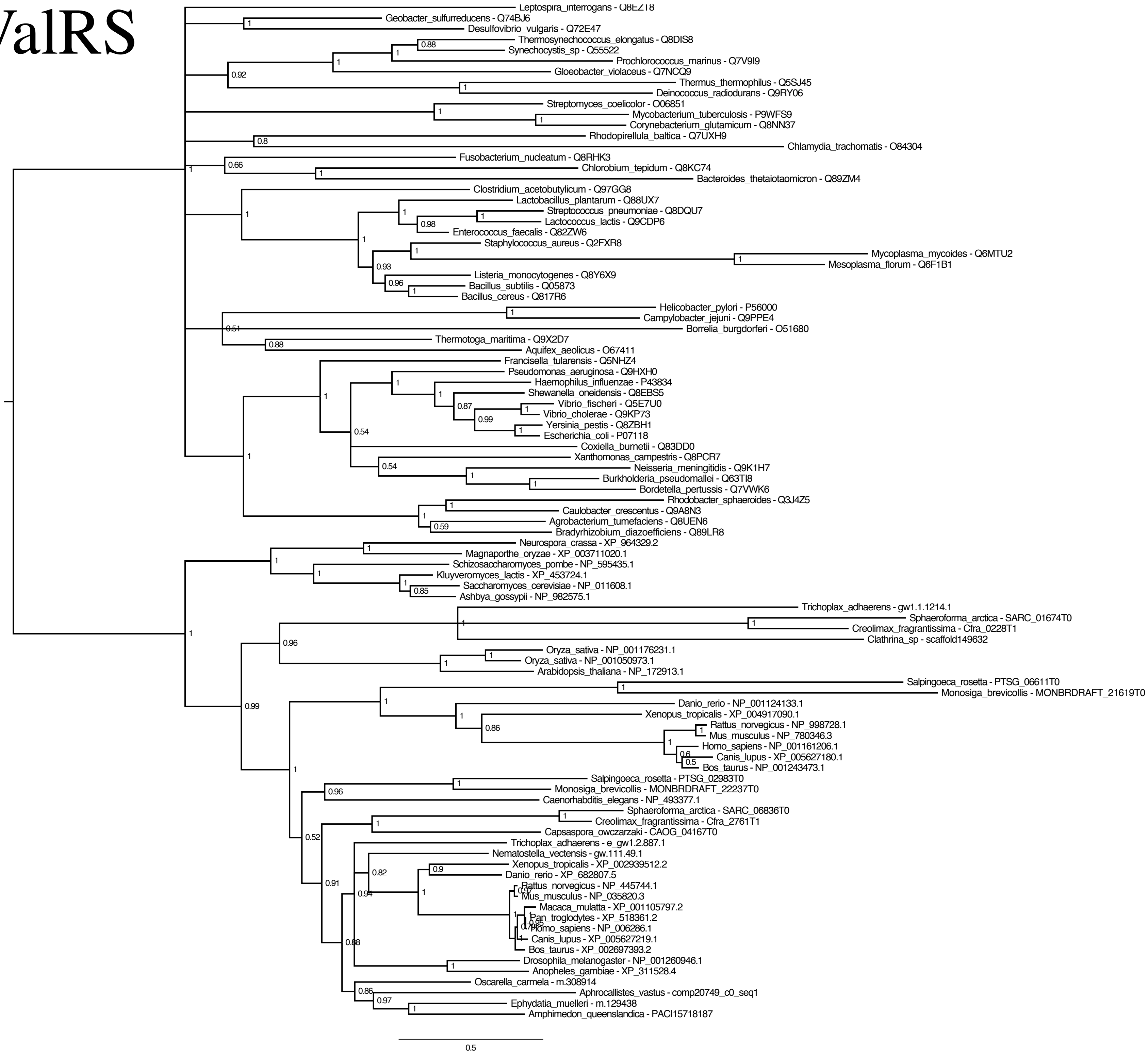

Leptospira_interrogans - Q8EZ18
Geobacter_sulfurreducens - Q74BJ6
Desulfovibrio_vulgaris - Q72E47
Thermosynechococcus_elongatus - Q8DIS8
Synechocystis_sp - Q55522
Prochlorococcus_marinus - Q7V9I9
Gloeobacter_violaceus - Q7NCQ9
Thermus_thermophilus - Q5SJ45
Deinococcus_radiodurans - Q9RY06
Streptomyces_coelicolor - O06851
Mycobacterium_tuberculosis - P9WFS9
Corynebacterium_glutamicum - Q8NN37
Rhodopirellula_baltica - Q7UXH9
Chlamydia_trachomatis - O84304
Fusobacterium_nucleatum - Q8RHK3
Chlorobium_tepidum - Q8KC74
Bacteroides_thetaiotaomicron - Q89ZM4
Clostridium_acetobutylicum - Q97GG8
Lactobacillus_plantarum - Q88UX7
Streptococcus_pneumoniae - Q8DQU7
Lactococcus_lactis - Q9CDP6
Enterococcus_faecalis - Q82ZW6
Staphylococcus_aureus - Q2FXR8
Mycoplasma_mycoides - Q6MTU2
Mesoplasma_florum - Q6F1B1
Listeria_monocytogenes - Q8Y6X9
Bacillus_subtilis - Q05873
Bacillus_cereus - Q817R6
Helicobacter_pylori - P56000
Campylobacter_jejuni - Q9PPE4
Borrelia_burgdorferi - O51680
Thermotoga_maritima - Q9X2D7
Aquifex_aeolicus - O67411
Francisella_tularensis - Q5NHZ4
Pseudomonas_aeruginosa - Q9HXH0
Haemophilus_influenzae - P43834
Shewanella_oneidensis - Q8EBS5
Vibrio_fischeri - Q5E7U0
Vibrio_cholerae - Q9KP73
Yersinia_pestis - Q8ZBH1
Escherichia_coli - P07118
Coxiella_burnetii - Q83DD0
Xanthomonas_campestris - Q8PCR7
Neisseria_meningitidis - Q9K1H7
Burkholderia_pseudomallei - Q63TI8
Bordetella_pertussis - Q7VWK6
Rhodobacter_sphaeroides - Q3J4Z5
Caulobacter_crescentus - Q9A8N3
Agrobacterium_tumefaciens - Q8UEN6
Bradyrhizobium_diazoefficiens - Q89LR8
Neurospora_crassa - XP_964329.2
Magnaporthe_oryzae - XP_003711020.1
Schizosaccharomyces_pombe - NP_595435.1
Kluyveromyces_lactis - XP_453724.1
Saccharomyces_cerevisiae - NP_011608.1
Ashbya_gossypii - NP_982575.1
Trichoplax_adhaerens - gw1.1.1214.1
Sphaeroforma_arctica - SARC_01674T0
Creolimax_fragrantissima - Cfra_0228T1
Clathrina_sp - scaffold149632
Oryza_sativa - NP_001176231.1
Oryza_sativa - NP_001050973.1
Arabidopsis_thaliana - NP_172913.1
Salpingoeca_rosetta - PTSG_06611T0
Monosiga_brevicollis - MONBRDRAFT_21619T0
Danio_rerio - NP_001124133.1
Xenopus_tropicalis - XP_004917090.1
Rattus_norvegicus - NP_998728.1
Mus_musculus - NP_780346.3
Homo_sapiens - NP_001161206.1
Canis_lupus - XP_005627180.1
Bos_taurus - NP_001243473.1
Salpingoeca_rosetta - PTSG_02983T0
Monosiga_brevicollis - MONBRDRAFT_22237T0
Caenorhabditis_elegans - NP_493377.1
Sphaeroforma_arctica - SARC_06836T0
Creolimax_fragrantissima - Cfra_2761T1
Capsaspora_owczarzaki - CAOG_04167T0
Trichoplax_adhaerens - e_gw1.2.887.1
Nematostella_vectensis - gw.111.49.1
Xenopus_tropicalis - XP_002939512.2
Danio_rerio - XP_682807.5
Rattus_norvegicus - NP_445744.1
Mus_musculus - NP_035820.3
Macaca_mulatta - XP_001105797.2
Pan_troglodytes - XP_518361.2
Homo_sapiens - NP_006286.1
Canis_lupus - XP_005627219.1
Bos_taurus - XP_002697393.2
Drosophila_melanogaster - NP_001260946.1
Anopheles_gambiae - XP_311528.4
Oscarella_carmela - m.308914
Aphrocallistes_vastus - comp20749_c0_seq1
Ephydatia_muelleri - m.129438
Amphimedon_queenslandica - PACI15718187
0.5

Supplementary Figure S3. Bayesian phylogenetic analysis of gat sequences. Trees were estimated using PhyloBayes MPI with the WAG+Γ amino acid substitution model. Two chains were run for 15,000 generations each. Convergence was assessed using the `bpcomp` program, ensuring that the `maxdiff` statistic was less than 0.3. **A.** GatA, **B.** GatB

A. GatA

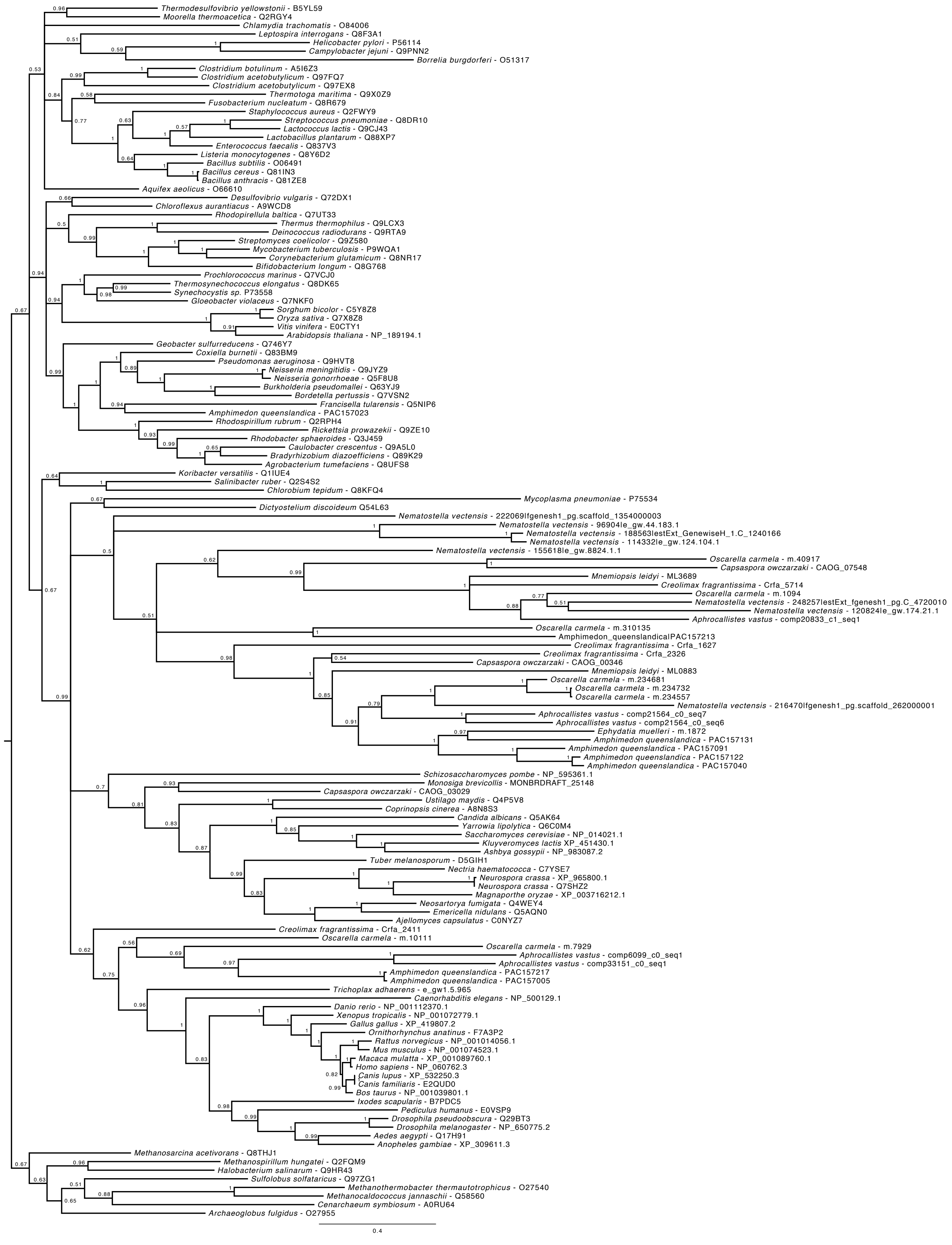

Thermodesulfovibrio yellowstonii - B5YL59
Moorella thermoacetica - Q2RGY4
Chlamydia trachomatis - O84006
Leptospira interrogans - Q8F3A1
Helicobacter pylori - P56114
Campylobacter jejuni - Q9PNN2
Borrelia burgdorferi - O51317
Clostridium botulinum - A5I6Z3
Clostridium acetobutylicum - Q97FQ7
Clostridium acetobutylicum - Q97EX8
Thermotoga maritima - Q9X0Z9
Fusobacterium nucleatum - Q8R679
Staphylococcus aureus - Q2FWY9
Streptococcus pneumoniae - Q8DR10
Lactococcus lactis - Q9CJ43
Lactobacillus plantarum - Q88XP7
Enterococcus faecalis - Q837V3
Listeria monocytogenes - Q8Y6D2
Bacillus subtilis - O06491
Bacillus cereus - Q81IN3
Bacillus anthracis - Q81ZE8
Aquifex aeolicus - O66610
Desulfovibrio vulgaris - Q72DX1
Chloroflexus aurantiacus - A9WCD8
Rhodopirellula baltica - Q7UT33
Thermus thermophilus - Q9LCX3
Deinococcus radiodurans - Q9RTA9
Streptomyces coelicolor - Q9Z580
Mycobacterium tuberculosis - P9WQA1
Corynebacterium glutamicum - Q8NR17
Bifidobacterium longum - Q8G768
Prochlorococcus marinus - Q7VCJ0
Thermosynechococcus elongatus - Q8DK65
Synechocystis sp. P73558
Gloeobacter violaceus - Q7NKF0
Sorghum bicolor - C5Y8Z8
Oryza sativa - Q7X8Z8
Vitis vinifera - E0CTY1
Arabidopsis thaliana - NP_189194.1
Geobacter sulfurreducens - Q746Y7
Coxiella burnetii - Q83BM9
Pseudomonas aeruginosa - Q9HVT8
Neisseria meningitidis - Q9JYZ9
Neisseria gonorrhoeae - Q5F8U8
Burkholderia pseudomallei - Q63YJ9
Bordetella pertussis - Q7VSN2
Francisella tularensis - Q5NIP6
Amphimedon queenslandica - PAC157023
Rhodospirillum rubrum - Q2RPH4
Rickettsia prowazekii - Q9ZE10
Rhodobacter sphaeroides - Q3J459
Caulobacter crescentus - Q9A5L0
Bradyrhizobium diazoefficiens - Q89K29
Agrobacterium tumefaciens - Q8UFS8
Koribacter versatilis - Q1IUE4
Salinibacter ruber - Q2S4S2
Chlorobium tepidum - Q8KFQ4
Mycoplasma pneumoniae - P75534
Dictyostelium discoideum Q54L63
Nematostella vectensis - 222069|fgenesh1_pg.scaffold_1354000003
Nematostella vectensis - 96904|e_gw.44.183.1
Nematostella vectensis - 188563|estExt_GenewiseH_1.C_1240166
Nematostella vectensis - 114332|e_gw.124.104.1
Nematostella vectensis - 155618|e_gw.8824.1.1
Oscarella carmela - m.40917
Capsaspora owczarzaki - CAOG_07548
Mnemiopsis leidyi - ML3689
Creolimax fragrantissima - Crfa_5714
Oscarella carmela - m.1094
Nematostella vectensis - 248257|estExt_fgenesh1_pg.C_4720010
Nematostella vectensis - 120824|e_gw.174.21.1
Aphrocallistes vastus - comp20833_c1_seq1
Oscarella carmela - m.310135
Amphimedon_queenslandica|PAC157213
Creolimax fragrantissima - Crfa_1627
Creolimax fragrantissima - Crfa_2326
Capsaspora owczarzaki - CAOG_00346
Mnemiopsis leidyi - ML0883
Oscarella carmela - m.234681
Oscarella carmela - m.234732
Oscarella carmela - m.234557
Nematostella vectensis - 216470|fgenesh1_pg.scaffold_262000001
Aphrocallistes vastus - comp21564_c0_seq7
Aphrocallistes vastus - comp21564_c0_seq6
Ephydatia muelleri - m.1872
Amphimedon queenslandica - PAC157131
Amphimedon queenslandica - PAC157091
Amphimedon queenslandica - PAC157122
Amphimedon queenslandica - PAC157040
Schizosaccharomyces pombe - NP_595361.1
Monosiga brevicollis - MONBRDRAFT_25148
Capsaspora owczarzaki - CAOG_03029
Ustilago maydis - Q4P5V8
Coprinopsis cinerea - A8N8S3
Candida albicans - Q5AK64
Yarrowia lipolytica - Q6C0M4
Saccharomyces cerevisiae - NP_014021.1
Kluyveromyces lactis XP_451430.1
Ashbya gossypii - NP_983087.2
Tuber melanosporum - D5GIH1
Nectria haematococca - C7YSE7
Neurospora crassa - XP_965800.1
Neurospora crassa - Q7SHZ2
Magnaporthe oryzae - XP_003716212.1
Neosartorya fumigata - Q4WEY4
Emericella nidulans - Q5AQN0
Ajellomyces capsulatus - C0NYZ7
Creolimax fragrantissima - Crfa_2411
Oscarella carmela - m.10111
Oscarella carmela - m.7929
Aphrocallistes vastus - comp6099_c0_seq1
Aphrocallistes vastus - comp33151_c0_seq1
Amphimedon queenslandica - PAC157217
Amphimedon queenslandica - PAC157005
Trichoplax adhaerens - e_gw1.5.965
Caenorhabditis elegans - NP_500129.1
Danio rerio - NP_001112370.1
Xenopus tropicalis - NP_001072779.1
Gallus gallus - XP_419807.2
Ornithorhynchus anatinus - F7A3P2
Rattus norvegicus - NP_001014056.1
Mus musculus - NP_001074523.1
Macaca mulatta - XP_001089760.1
Homo sapiens - NP_060762.3
Canis lupus - XP_532250.3
Canis familiaris - E2QUD0
Bos taurus - NP_001039801.1
Ixodes scapularis - B7PDC5
Pediculus humanus - E0VSP9
Drosophila pseudoobscura - Q29BT3
Drosophila melanogaster - NP_650775.2
Aedes aegypti - Q17H91
Anopheles gambiae - XP_309611.3
Methanosarcina acetivorans - Q8THJ1
Methanospirillum hungatei - Q2FQM9
Halobacterium salinarum - Q9HR43
Sulfolobus solfataricus - Q97ZG1
Methanothermobacter thermautotrophicus - O27540
Methanocaldococcus jannaschii - Q58560
Cenarchaeum symbiosum - A0RU64
Archaeoglobus fulgidus - O27955
0.4

B. GatB

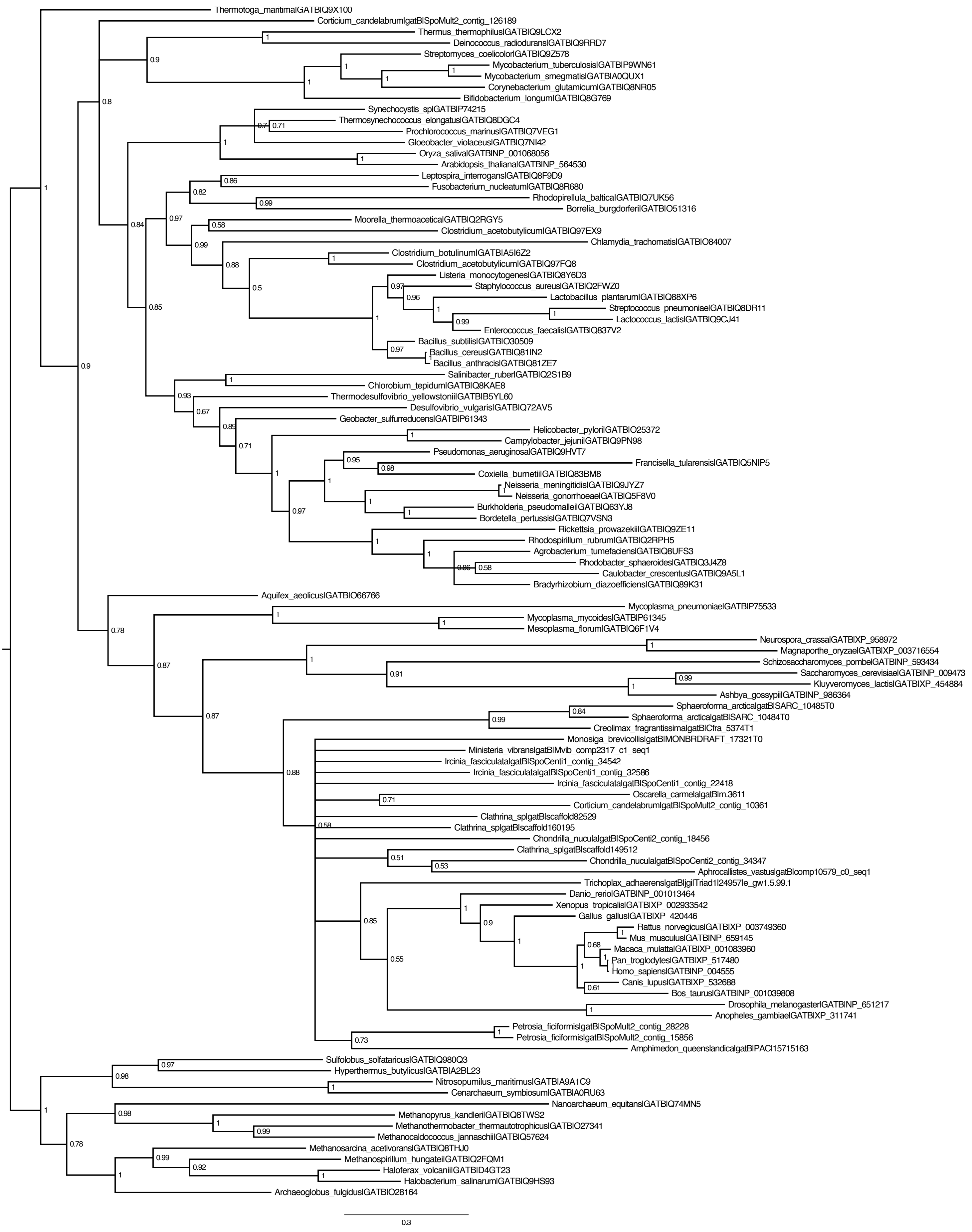

Thermotoga_maritima|GATB|Q9X100
Corticium_candelabrum|gatB|SpoMult2_contig_126189
Thermus_thermophilus|GATB|Q9LCX2
Deinococcus_radiodurans|GATB|Q9RRD7
Streptomyces_coelicolor|GATB|Q9Z578
Mycobacterium_tuberculosis|GATB|P9WN61
Mycobacterium_smegmatis|GATB|A0QUX1
Corynebacterium_glutamicum|GATB|Q8NR05
Bifidobacterium_longum|GATB|Q8G769
Synechocystis_sp|GATB|P74215
Thermosynechococcus_elongatus|GATB|Q8DGC4
Prochlorococcus_marinus|GATB|Q7VEG1
Gloeobacter_violaceus|GATB|Q7NI42
Oryza_sativa|GATB|NP_001068056
Arabidopsis_thaliana|GATB|NP_564530
Leptospira_interrogans|GATB|Q8F9D9
Fusobacterium_nucleatum|GATB|Q8R680
Rhodopirellula_baltica|GATB|Q7UK56
Borrelia_burgdorferi|GATB|O51316
Moorella_thermoacetica|GATB|Q2RGY5
Clostridium_acetobutylicum|GATB|Q97EX9
Chlamydia_trachomatis|GATB|O84007
Clostridium_botulinum|GATB|A5I6Z2
Clostridium_acetobutylicum|GATB|Q97FQ8
Listeria_monocytogenes|GATB|Q8Y6D3
Staphylococcus_aureus|GATB|Q2FWZ0
Lactobacillus_plantarum|GATB|Q88XP6
Streptococcus_pneumoniae|GATB|Q8DR11
Lactococcus_lactis|GATB|Q9CJ41
Enterococcus_faecalis|GATB|Q837V2
Bacillus_subtilis|GATB|O30509
Bacillus_cereus|GATB|Q81IN2
Bacillus_anthracis|GATB|Q81ZE7
Salinibacter_ruber|GATB|Q2S1B9
Chlorobium_tepidum|GATB|Q8KAE8
Thermodesulfovibrio_yellowstonii|GATB|B5YL60
Desulfovibrio_vulgaris|GATB|Q72AV5
Geobacter_sulfurreducens|GATB|P61343
Helicobacter_pylori|GATB|O25372
Campylobacter_jejuni|GATB|Q9PN98
Pseudomonas_aeruginosa|GATB|Q9HVT7
Francisella_tularensis|GATB|Q5NIP5
Coxiella_burnetii|GATB|Q83BM8
Neisseria_meningitidis|GATB|Q9JYZ7
Neisseria_gonorrhoeae|GATB|Q5F8V0
Burkholderia_pseudomallei|GATB|Q63YJ8
Bordetella_pertussis|GATB|Q7VSN3
Rickettsia_prowazekii|GATB|Q9ZE11
Rhodospirillum_rubrum|GATB|Q2RPH5
Agrobacterium_tumefaciens|GATB|Q8UFS3
Rhodobacter_sphaeroides|GATB|Q3J4Z8
Caulobacter_crescentus|GATB|Q9A5L1
Bradyrhizobium_diazoefficiens|GATB|Q89K31
Aquifex_aeolicus|GATB|O66766
Mycoplasma_pneumoniae|GATB|P75533
Mycoplasma_mycoides|GATB|P61345
Mesoplasma_florum|GATB|Q6F1V4
Neurospora_crassa|GATB|XP_958972
Magnaporthe_oryzae|GATB|XP_003716554
Schizosaccharomyces_pombe|GATB|NP_593434
Saccharomyces_cerevisiae|GATB|NP_009473
Kluyveromyces_lactis|GATB|XP_454884
Ashbya_gossypii|GATB|NP_986364
Sphaeroforma_arctica|gatB|SARC_10485T0
Sphaeroforma_arctica|gatB|SARC_10484T0
Creolimax_fragrantissima|gatB|Cfra_5374T1
Monosiga_brevicollis|gatB|MONBRDRAFT_17321T0
Ministeria_vibrans|gatB|Mvib_comp2317_c1_seq1
Ircinia_fasciculata|gatB|SpoCenti1_contig_34542
Ircinia_fasciculata|gatB|SpoCenti1_contig_32586
Ircinia_fasciculata|gatB|SpoCenti1_contig_22418
Oscarella_carmela|gatB|m.3611
Corticium_candelabrum|gatB|SpoMult2_contig_10361
Clathrina_sp|gatB|scaffold82529
Clathrina_sp|gatB|scaffold160195
Chondrilla_nucula|gatB|SpoCenti2_contig_18456
Clathrina_sp|gatB|scaffold149512
Chondrilla_nucula|gatB|SpoCenti2_contig_34347
Aphrocallistes_vastus|gatB|comp10579_c0_seq1
Trichoplax_adhaerens|gatB|jgi|Triad1|24957|e_gw1.5.99.1
Danio_rerio|GATB|NP_001013464
Xenopus_tropicalis|GATB|XP_002933542
Gallus_gallus|GATB|XP_420446
Rattus_norvegicus|GATB|XP_003749360
Mus_musculus|GATB|NP_659145
Macaca_mulatta|GATB|XP_001083960
Pan_troglodytes|GATB|XP_517480
Homo_sapiens|GATB|NP_004555
Canis_lupus|GATB|XP_532688
Bos_taurus|GATB|NP_001039808
Drosophila_melanogaster|GATB|NP_651217
Anopheles_gambiae|GATB|XP_311741
Petrosia_ficiformis|gatB|SpoMult2_contig_28228
Petrosia_ficiformis|gatB|SpoMult2_contig_15856
Amphimedon_queenslandica|gatB|PAC|15715163
Sulfolobus_solfataricus|GATB|980Q3
Hyperthermus_butylicus|GATB|A2BL23
Nitrosopumilus_maritimus|GATB|A9A1C9
Cenarchaeum_symbiosum|GATB|A0RU63
Nanoarchaeum_equitans|GATB|Q74MN5
Methanopyrus_kandleri|GATB|Q8TWS2
Methanothermobacter_thermautotrophicus|GATB|O27341
Methanocaldococcus_jannaschii|GATB|Q57624
Methanosarcina_acetivorans|GATB|Q8THJ0
Methanospirillum_hungatei|GATB|Q2FQM1
Haloferax_volcanii|GATB|D4GT23
Halobacterium_salinarum|GATB|Q9HS93
Archaeoglobus_fulgidus|GATB|O28164
0.3

Supplementary Figure S4. Bayesian phylogenetic analysis of methionyl- and isoleucyl- mt-tRNA sequences. Sequences were obtained from the complete mt-genome sequences of 18 sponges, including 6 glass sponges, as well as 2 unicellular relatives of animals (*Capsaspora owczarzaki* and *Monosiga brevicollis*). Trees were estimated using MrBayes 3.2 using a mixture of all nucleotide substitution models (implemented using Reversible Jump MCMC), plus gamma-distributed rates across sites discretized into 4 categories. Two runs of 4 chains each were run for 10,000,000 generations. Convergence was assessed using the `bpcomp` program in the PhyloBayes package, ensuring that the `maxdiff` statistic was less than 0.1. The tree was rooted using prolyl-mt-tRNA sequences as an outgroup.

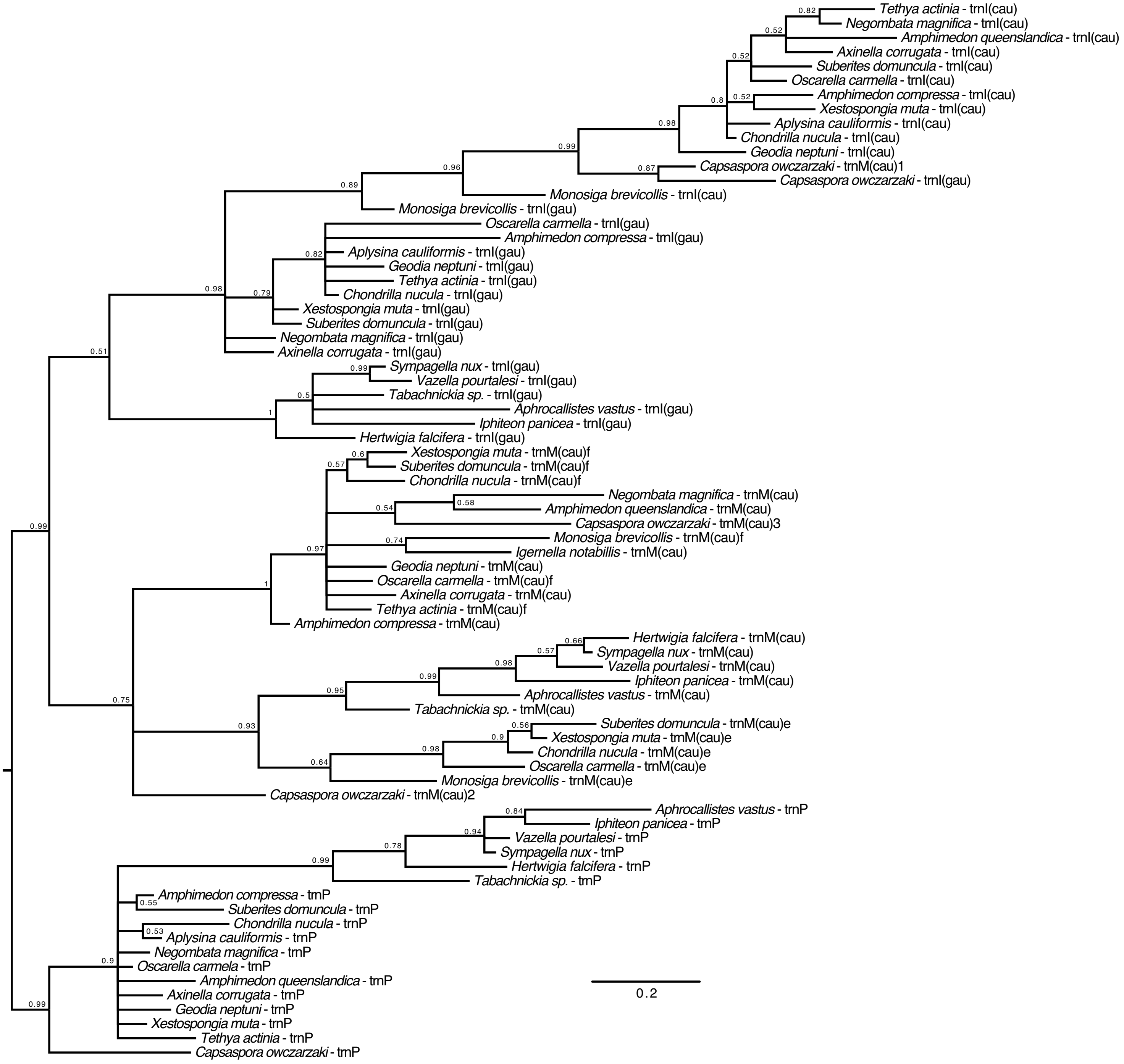

Supplement: Supplementary Data [file supp_evv124_suppl_data.zip › FiguresS1-4.pdf]
